# Origin and Evolution of Dishevelled

**DOI:** 10.1534/g3.112.005314

**Published:** 2013-02-01

**Authors:** Adler R. Dillman, Paul J. Minor, Paul W. Sternberg

**Affiliations:** Howard Hughes Medical Institute, Division of Biology, California Institute of Technology, Pasadena, California 91125

**Keywords:** dishevelled, protein evolution, Nematoda, Wnt, *C. elegans*

## Abstract

Dishevelled (Dsh or Dvl) is an important signaling protein, playing a key role in Wnt signaling and relaying cellular information for several developmental pathways. Dsh is highly conserved among metazoans and has expanded into a multigene family in most bilaterian lineages, including vertebrates, planarians, and nematodes. These orthologs, where explored, are known to have considerable overlap in function, but evidence for functional specialization continues to mount. We performed a comparative analysis of Dsh across animals to explore protein architecture and identify conserved and divergent features that could provide insight into functional specialization with an emphasis on invertebrates, especially nematodes. We find evidence of dynamic evolution of Dsh, particularly among nematodes, with taxa varying in ortholog number from one to three. We identify a new domain specific to some nematode lineages and find an unexpected nuclear localization signal conserved in many Dsh orthologs. Our findings raise questions of protein evolution in general and provide clues as to how animals have dealt with the complex intricacies of having a protein, such as Dsh, act as a central messenger hub connected to many different and vitally important pathways. We discuss our findings in the context of functional specialization and bring many testable hypotheses to light.

Dishevelled (Dsh or Dvl) is a multifunctional phosphoprotein originally discovered in *Drosophila* and named for its disruptions in hair and bristle polarity (Fahmy and Fahmy 1959; Klingensmith *et al.* 1994). Dsh plays a key role in Wnt signaling, thus affecting cell proliferation, migration, polarity, terminal differentiation, and the self-renewal of stem cells (Boutros and Mlodzik 1999; Gao and Chen 2010; Sugimura *et al.* 2012; Tauriello *et al.* 2012). Deregulation of pathway components is associated with multiple human diseases (Luo *et al.* 2007). Wnt signaling has evolved to act in multiple pathways, broadly divided into the canonical/β-catenin−dependent pathway and the noncanonical/β-catenin−independent pathway, with Dsh acting in a key role, relaying signals from receptors to downstream effectors (Gao and Chen 2010). Several components of the Wnt signaling pathway, including Frizzled, GSK3, and β-catenin, can be found in protozoans, but it is not until the emergence of Metazoa that a complete Wnt pathway can be found (Holstein 2012). The early branching metazoan lineage Porifera has only the major components of the canonical pathway, with critical noncanonical pathway components arising subsequently in eumetazoan lineages (Adamska *et al.* 2007, 2010; Kusserow *et al.* 2005). Thus, although Wnt signaling is conserved across Metazoa from sponges to humans, it seems that this pathway’s origin and the original role of Dsh lies in the canonical/β-catenin−dependent pathway, with noncanonical signaling developing later. Figure 1 summarizes the evolution of Dsh across animals. including the loss and gain of orthologs, paralogs, and protein domains.

**Figure 1:**
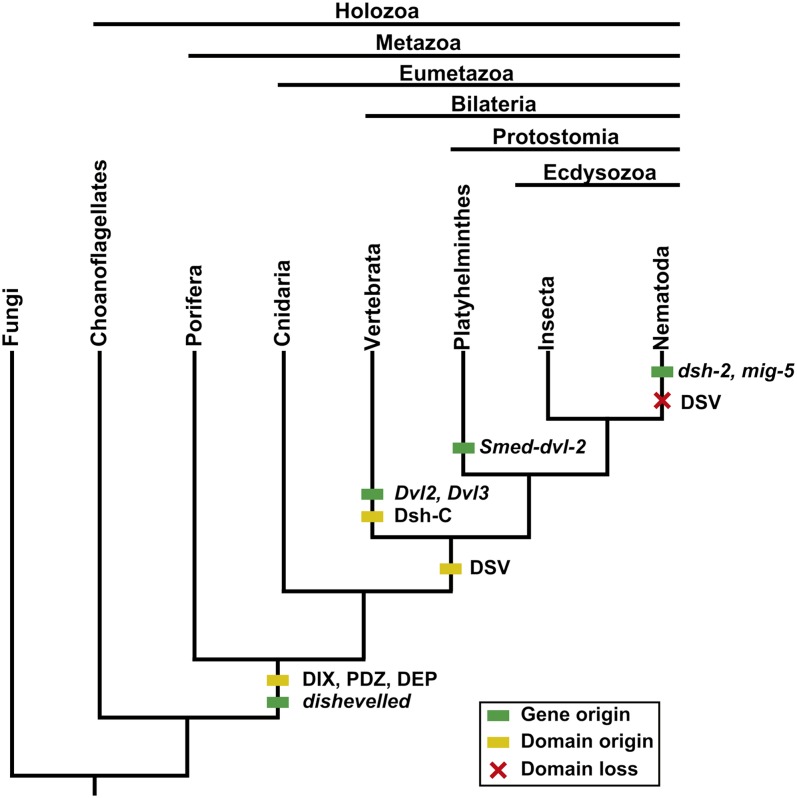
A cladogram showing key features during the evolution of Dsh among animals including the origin of Dsh and lineage-specific paralogs as well as the gain or loss of protein domains.

In the current model of the canonical pathway, Wnt signals are received by a Frizzled (Fz) and the LRP5/6 coreceptor complex, leading to the recruitment of Dsh and Axin to the cell membrane. This recruitment results in the inactivation and dissolution of the β-catenin destruction complex, allowing for the nuclear translocation of β-catenin, where it interacts with members of the TCF/Lef transcription family to regulate gene expression (Wharton 2003). Noncanonical signaling encompasses several different pathways that do not necessarily lead to the activation of β-catenin, of which the best understood is the planar cell polarity (PCP) pathway. PCP signaling is responsible for the polarization of cells along an epithelial sheet. The core components of this pathway include the transmembrane proteins Fz, Flamingo (Fmi), and Strabismus (Stbm), as well as the cytoplasmic proteins Diego (Dgo), Prickle (Pk), and Dsh (Seifert and Mlodzik 2007). In general PCP signaling relies on complex interactions between these core components that lead to their asymmetric enrichment and distribution within a cell. For example, during polarization in the *Drosophila* wing two distinct protein complexes antagonize each other and localize to opposite ends of the cell: a Fz-Dsh-Dgo complex becomes enriched at the distal end of each cell, whereas a Stbm-Pk complex concentrates proximally (Simons and Mlodzik 2008).

The literature establishes the archetypal Dsh protein to contain three conserved domains: an N-terminal DIX (Dishevelled and Axin) domain, a central PDZ (Post Synaptic Density-95, Discs Large, and Zonula occludens-1) domain, and a C-terminal DEP (Dishevelled, EGL-10, Pleckstrin) domain (Figure 2) (Gao and Chen 2010; Penton *et al*. 2002). In addition to these three conserved domains, Dsh is known to contain a basic region that precedes the N-terminus of the PDZ domain as well as a proline-rich region that includes an SH3 binding motif located between the PDZ and DEP domains (Figure 2). Both of these regions are thought to be conserved in most Dsh orthologs and have functional significance. There is a fourth domain reported to be conserved in Dsh, the DSV or Dishevelled domain, although its functional significance is rarely discussed (Figure 2). The Dsh protein contains approximately 15% serine and threonine residues, many of which are phosphorylated; however, the functional significance of these residues has been questioned (Yanfeng *et al.* 2011). Expansions of Dsh among metazoan lineages seem common and variable, with most vertebrates containing three Dsh homologs (although the chicken *Gallus gallus* has two), whereas insects have only one (Gao and Chen 2010; Gray *et al.* 2009; Klingensmith *et al.* 1994; Sweetman *et al.* 2008). The nematode *Caenorhabditis elegans* has three Dsh homologs, and the planarian *Schmidtea mediterranea* has two (Figure 1) (Gurley *et al.* 2008; Ruvkun and Hobert 1998). Table 1 lists the taxa and Dsh ortholog abbreviations used throughout the text.

**Figure 2:**
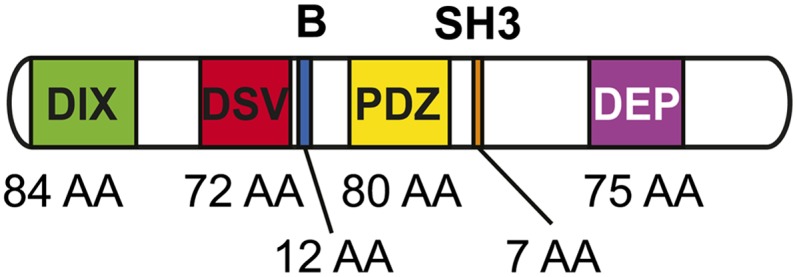
Diagram showing the archetypal Dsh protein with conserved domains and motifs. From left to right: the DIX domain, the DSV or dishevelled domain, the basic region, the PDZ domain, the SH3 binding motif, which is often referred to as the proline-rich region, and the DEP domain.

**Table 1:** Dsh abbreviations

Species	Abbreviation(s)
Nematoda	
*Meloidogyne incognita*	*Minc-dsh-1*, *Minc-mig-5*
*Meloidogyne hapla*	*Minc-dsh-1*, *Minc-mig-5*
*Panagrellus redivivus*	*Pred-dsh-1*, *Pred-mig-5*
*Bursaphelenchus xylophilus*	*Bxyl-dsh-1*, *Bxyl-mig-5*
*Steinernema carpocapsae*	*Scar-dsh-1*, *Scar-mig-5*
*Caenorhabditis briggsae*	*Cbri-dsh-1*, *Cbri-dsh-2*, *Cbri-mig-5*
*Caenorhabditis remanei*	*Crem-dsh-1*, *Crem-dsh-1*, *Crem-mig-5*
*Caenorhabditis brenneri*	*Cbre-dsh-1*, *Cbre-dsh-2*, *Cbre-mig-5*
*Caenorhabditis elegans*	*Cele-dsh-1*, *Cele-dsh-2*, *Cele-mig-5*
*Caenorhabditis japonica*	*Cjap-dsh-1*, *Cjap-dsh-2*, *Cjap-mig-5*
*Caenorhabditis angaria*	*Cang-dsh-1*, *Cang-mig-5*
*Pristionchus pacificus*	*Ppac-dsh-1*, *Ppac-mig-5*
*Brugia malayi*	*Bmal-dsh-1*, *Bmal-mig-5*
*Ascaris suum*	*Asuu-dsh-1*, *Asuu-mig-5*
*Trichinella spiralis*	*Tspi-dsh-1*
Arthropoda	
*Nasonia vitripennis* (parasitoid wasp)	*Nvit-dsh*
*Tribolium castaneum* (red flour beetle)	*Tcas-dsh*
*Drosophila melanogaster* (fruit fly)	*Dmel-dsh*
Platyhelminthes	
*Schmidtea mediterranea**^a^* (planaria)	*Smed-dsh-1*, *Smed-dvl-2*
Chordata	
*Xenopus tropicalis*	*Xtro-Dvl1*, *Xtro-Dvl2*, *Xtro-Dvl3*
*Mus musculus*	*Mmus-Dvl1*, *Mmus-Dvl2*, *Mmus-Dvl3*
*Homo sapiens*	*Hsap-Dvl1*, *Hsap-Dvl2*, *Hsap-Dvl3*
*Ciona intestinalis**^a^* (sea squirt)	*Cint-Dvl*
Cnidaria	
*Clytia hemisphaerica**^a^* (jellyfish)	*Chem-Dvl*
Porifera	
*Amphimedon queenslandica**^a^* (sponge)	*Aque-Dvl*

All taxa analyzed along with their taxonomic phylum and the abbreviations used to represent their Dsh orthologs.

a Dsh orthologs for these taxa were taken from Genbank rather than identified through whole genome searches.

With the ever-increasing amount of genomic data available for analysis, we leveraged the currently available data to study the evolution of Dsh across animals with an emphasis on nematodes. In addition to exploring the potential conservation of the three *C. elegans* Dsh homologs among nematodes, we were interested in identifying conserved or divergent protein features that correlate with the known functional divergence between Dsh orthologs observed in several animal taxa and that could provide hypotheses about the evolution of Dsh. For example, the planarian Dsh paralogs, *Smed-dvl-1* and *Smed-dvl-2*, appear to be functionally specialized such that only *Smed-dvl-2* is thought to be involved in β-catenin−dependent signal transduction, suggesting underlying physical differences in these proteins that have not yet been linked to their divergent function (Almuedo-Castillo *et al.* 2011). Similarly, the function of Dsh orthologs seems to have diverged among vertebrates, where *Dvl1* and *Dvl2*, but not *Dvl3*, are necessary to mediate the Wnt-dependent signals that control neural crest specification in *Xenopus*, but in murines it is thought that *Dvl2* and *Dvl3* function in neural crest development whereas *Dvl1* apparently does not (Etheridge *et al.* 2008; Gray *et al.* 2009; Hamblet *et al.* 2002; Lijam *et al.* 1997; Monsoro-Burq *et al.* 2005). It is still not known whether Dsh’s role in neural crest development is through the canonical or noncanonical pathways or both (Etheridge *et al.* 2008). In our study we found that Dishevelled is a highly conserved protein that has undergone dynamic evolution across metazoans and variation in protein architecture provides clues about its functional roles in β-catenin−dependent and −independent pathways.

## Materials and Methods

### Orthology analyses

To study the evolution of Dishevelled, we used the available predicted protein datasets from WormBase release WS225 (www.wormbase.org) for the following species: *Brugia malayi*, *Caenorhabditis elegans*, *Caenorhabditis angaria*, *Caenorhabditis japonica*, *Caenorhabditis brenneri*, *Caenorhabditis remanei*, *Caenorhabditis briggsae*, *Meloidogyne hapla*, *Pristionchus pacificus*, and *Trichinella spiralis*. We also included the *Ascaris suum*, *Bursaphelenchus xylophilus*, and *Meloidogyne incognita* predicted proteome data sets from WormBase release WS229. For outgroup and comparative analysis, we used the predicted protein datasets of *Arabidopsis thaliana* (vGNOMON 7/9/07), *Drosophila melanogaster* (v10/30/11), *Homo sapiens* (v9/7/11), *Mus musculus* (v3/4/11), *Nasonia vitripennis* (v1.2), *Saccharomyces cerevisiae* (v2/3/11), and *Tribolium castaneum* (vTcas 3.0) genome projects, obtained from the National Center for Biotechnology Information/National Institutes of Health repository (ftp://ftp.ncbi.nih.gov/genomes). Pre-released proteomes for *Panagrellus redivivus* and *Steinernema carpocapsae* also were used from manuscripts in preparation (A. R. Dillman, A. Mortazavi, M. Macchietto, C. F. Porter, and H. Goodrich-Blair, unpublished data; Srinivasan *et al.* 2013; Dillman *et al.* 2012) Dsh orthologs from the jellyfish *Clytia hemisphaerica* (AFI99114.1), the planarian *Schmidtea mediterranea* (Smed-DVL-1 ADZ58511.1 and Smed-DVL-2 ADZ58512.1), the frog *Xenopus tropicalis* (DVL1 NP_001116886.1, DVL2 NP_001072660.1, and DVL3 NP_01116929.1), the sponge *Amphimedon queenslandica* (XP_003384321), and the tunicate *Ciona intestinalis* (NP_001027754.1) were acquired from GenBank (http://blast.ncbi.nlm.nih.gov/).

Version 1.4 of the OrthoMCL pipeline was used to cluster proteins from the proteomes into families of orthologous genes (http://www.orthomcl.org) (Li *et al.* 2003). To identify orthologs of Dsh across animals, we ran OrthoMCL using the full proteomes of *C. elegans*, *P. redivivus*, *T. spiralis*, *N. vitripennis*, *D. melanogaster*, *T. castaneum*, *M. musculus*, *H. sapiens*, *S. cerevisiae*, and *A. thaliana*. To identify orthologs across Nematoda, we ran OrthoMCL using the full proteomes of *B. malayi*, *A. suum*, *P. pacificus*, *C. elegans*, *B. xylophilus*, *M. hapla*, *M. incognita*, *P. redivivus*, *S. carpocapsae*, *T. spiralis*, with *N. vitripennis* as an outgroup. To identify orthologs within *Caenorhabditis* we ran OrthoMCL using the full proteomes of *C. angaria*, *C. briggsae*, *C. brenneri*, *C. japonica*, *C. remanei*, and *C. elegans*. All orthology analyses were run using OrthoMCL version 1.4 with default settings and the BLAST parameters recommended in the OrthoMCL documentation (Li *et al.* 2003).

### Domain analysis

Each identified Dsh ortholog was analyzed for protein domains using the SMART protein domain analysis website (http://smart.embl-heidelberg.de), used in normal mode (Letunic *et al.* 2012). All additional options (outlier homologs, PFAM domain, signal peptides, internal repeats, and intrinsic protein disorder) were turned on for the analysis. The full protein sequences and identified domains are available in the Supporting Information, File S1.

### Sequence alignment, phylogenetics, and selection detection

Sequence alignments were made using all of the amino acid sequence from the beginning of the PDZ domain to the end of the DEP domain because these were the only identified domains conserved across all of the animal taxa we evaluated. Protein sequence alignments of this region were made using the online MUSCLE service (http://www.ebi.ac.uk/Tools/msa/muscle) (Edgar 2004). These protein alignments were then replaced with the appropriate nucleotide sequences using the RevTrans server (http://www.cbs.dtu.dk/services/RevTrans), which preserves the alignment obtained from the amino acid sequences but replaces each amino acid with the user-supplied protein coding nucleotides (Wernersson and Pedersen 2003). Coding sequence for the proteomes was downloaded along with the proteomes from the sites listed previously, although the sequences for most of the nematodes we used could also be acquired from WormBase (www.wormbase.org). Two separate alignments were made using this method, one that included the Dsh orthologs across animals, including *T. castaneum*, *N. vitripennis*, *D. melanogaster*, *M. musculus*, *H. sapiens*, *T. spiralis*, *A. suum*, *B. malayi*, *P. pacificus*, *C. elegans*, *S. carpocapsae*, *B. xylophilus*, *P. redivivus*, *M. hapla*, *M. incognita*, and the jellyfish *C. hemisphaerica*. The other alignment focused on Dsh orthologs within caenorhabditid nematodes, using genes from *C. elegans*, *C. angaria*, *C. japonica*, *C. brenneri*, *C. remanei*, *C. briggsae*, with the intracellular parasite *T. spiralis* and the parasitoid wasp *N. vitripennis* as outgroups. Alignments were then shaded to reflect sequence conservation using GeneDoc (http://www.nrbsc.org/gfx/genedoc) (Nicholas *et al.* 1997).

The nucleotide alignments were then evaluated for the best-fit model of evolution using jModelTest2 (http://code.google.com/p/jmodeltest2) (Darriba *et al.* 2012; Guindon and Gascuel 2003). For the alignment across animals, the corrected Akaike information criterion, the Bayesian inference criterion, and the decision theory criterion all selected the GTR+I+G model of evolution, with a p-invar = 0.0640 and a gamma shape parameter of 0.9840. The analysis of the *Caenorhabditis* alignment resulted in the GTR+G model being chosen by all criteria, with a gamma shape parameter of 0.5140.

Following model selection, maximum likelihood (ML) analyses with 1000 bootstraps were done using the RAxML BlackBox server (http://phylobench.vital-it.ch/raxml-bb) (Stamatakis *et al.* 2008). New technology parsimony analyses were done using TNT (http://www.cladistics.com/aboutTNT.html) (Goloboff 1999; Nixon 1999). Maxtrees was set to 10,000. A new technology, random driven search was performed using ratchet, drift, and tree fusing options. A bootstrap analysis of 1000 was performed by resampling.

Selection was detected using two methods. First, the alignment files of the protein-coding nucleotide sequences were uploaded into MEGA 5.05 (http://www.megasoftware.net) (Tamura *et al.* 2011). The selection analysis option in MEGA, which estimates selection for each codon using HyPhy, was used. Our ML analysis served as the guide tree, and the ML statistical method was chosen using the GTR model, as selected by jModelTest2. All sites were used in the analysis. Following the MEGA analysis of selection, we used the HyPhy package as implemented by the Datamonkey adaptive evolutionary server (http://www.datamonkey.org). Alignment files with the ML phylogenetic analysis written into them were uploaded using the codon data type and the universal genetic code. We used the recommended meme method in our analyses, setting the options to estimate the global dN/dS value and to average encountered ambiguities in the consensus sequence (Murrell *et al.* 2012). We set the level of significance at *P* = 0.1.

## Results

### Dishevelled conservation and expansion among animals

We evaluated the conservation and potential expansion of Dsh by using cluster analysis of seventeen whole proteomes, including vertebrates, insects, nematodes, and a fungal and plant proteome as outgroups (see *Materials and Methods*). We found no evidence of Dsh or Dsh-like genes outside Metazoa. It was previously known that *D. melanogaster* and potentially all insects have one Dsh (*Dmel-dsh*), the model nematode *C. elegans* has three Dsh homologs (*Cele-dsh-1*, *Cele-dsh-2*, and *Cele-mig-5*), the planarian *Schmidtea mediterranea* has two (*Smed-dvl-1* and *Smed-dvl-2*), and most vertebrates have three (*Dvl1*, *Dvl2*, and *Dvl3*; Figure 1). We found three distinct clusters of Dsh genes, the largest included all of the insect orthologs (*Dmel-dsh*, *Nvit-dsh*, *Tcas-dsh*; one copy in each insect proteome), all nematode *dsh-1* orthologs, and the vertebrate orthologs of *Dvl1* and *Dvl3* (*Mmus-Dvl1*, *Mmus-Dvl-3*, *Hsap-Dvl1*, and *Hsap-Dvl3*). A second cluster included exclusively nematode *mig-5* genes, whereas the vertebrate *Dvl2* orthologs (*Mmus-Dvl2* and *Hsap-Dvl2*) formed their own cluster, apparently having no orthologs outside vertebrates. *Cele-dsh-2* remained an unclustered orphan in this broad analysis.

**Figure 3:**
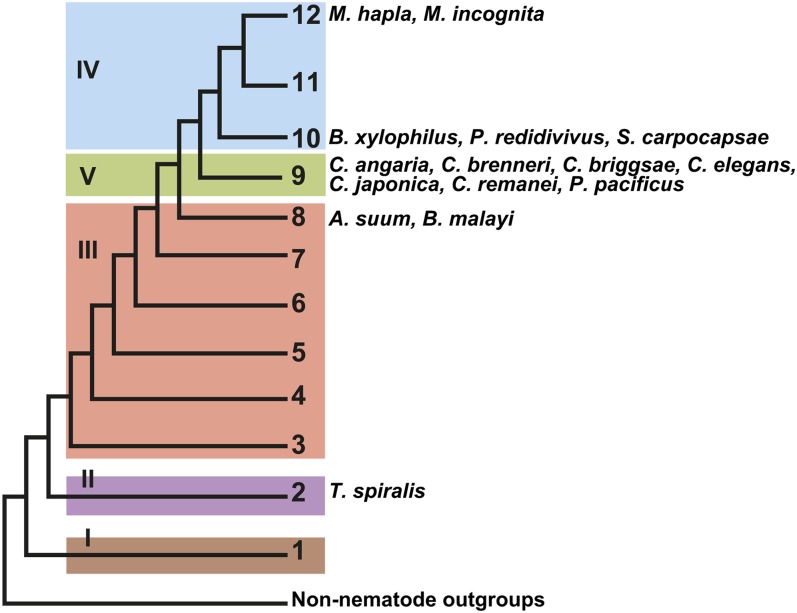
A schematic representation of the division of the phylum Nematoda into clades, with the 12-clade designation after Holterman *et al.* 2006 (Holterman *et al.* 2006) and the five-clade designation after Blaxter *et al.* 1998 (Blaxter *et al.* 1998) in Roman numerals. Blaxter clades are encompassed in colored boxes.

Using the three *C. elegans* Dsh homologs (*Cele-dsh-1*, *Cele-dsh-2*, and *Cele-mig-5*) as queries, we found that only *Cele-dsh-1* has orthologs outside of Nematoda, being highly conserved across metazoans, with all insects and nematodes having only one strict ortholog, and vertebrates having two, *Dvl1* and *Dvl3*. Among the nematode genera in this analysis, *C. elegans* is unique in having three Dsh homologs. In addition to having an ortholog of *dsh-1*, most nematodes also have an ortholog of *mig-5*, but none of the nematodes in this analysis have orthologs of *Cele-dsh-2*. Unlike the rest of the nematodes we studied, *T. spiralis*, which is in the basal clade 2 of Nematoda, retains only a single Dsh ortholog (*Tspi-dsh-1*) (Figure 3) (Holterman *et al.* 2006). This result shows that Dsh has experienced dynamic evolution within Nematoda, with extant taxa possessing one, two, or three Dsh homologs.

**Figure 4:**
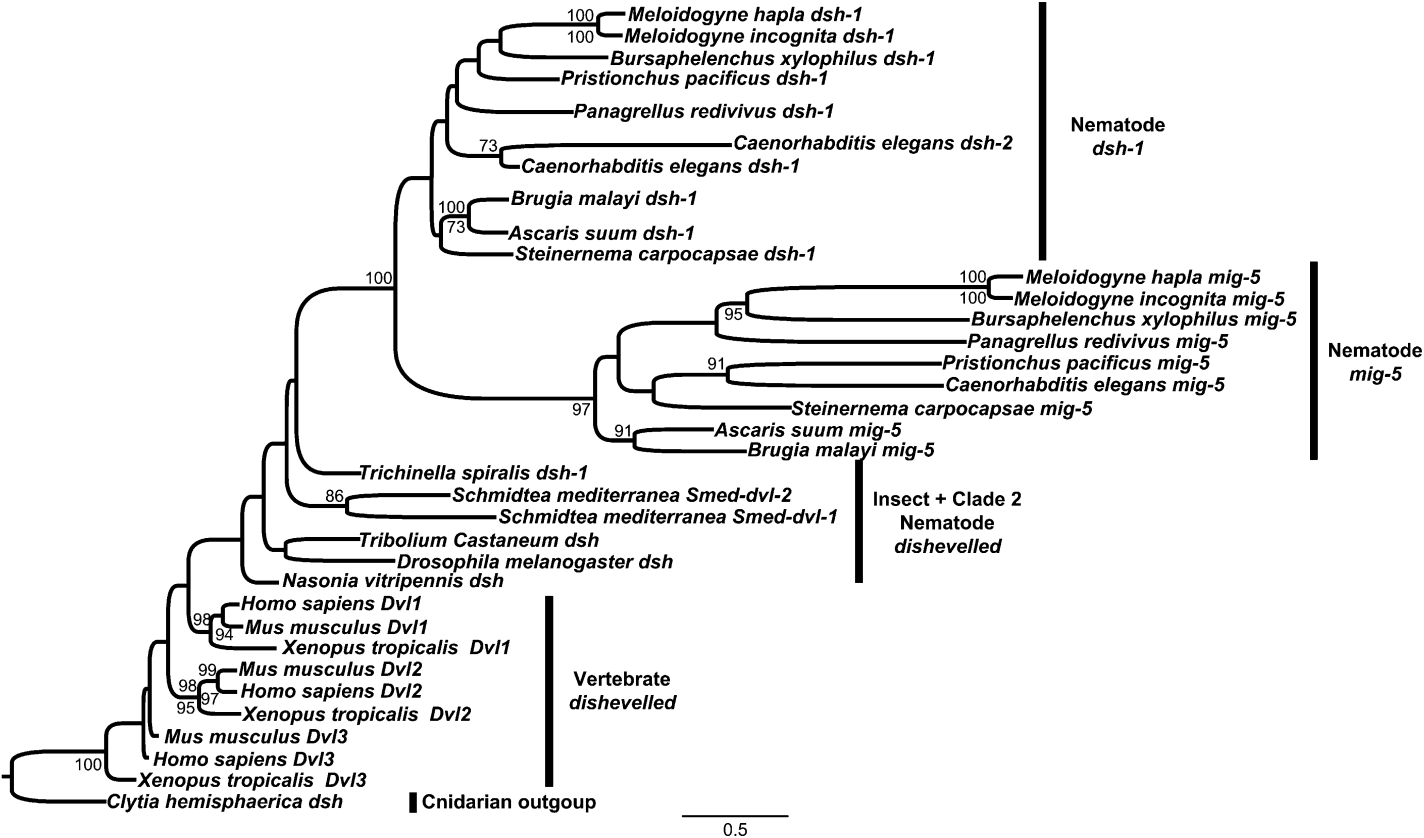
Phylogenetic analysis of Dsh orthologs across animals based on the protein coding nucleotide alignment from the N-terminus of the PDZ domain through the C-terminus of the DEP domain. The ML tree (rooted with the outgroup *C. hemisphaerica*) is shown. For each node, ML bootstrap support values (1000 replicates) are above the nodes whereas parsimony bootstrap values (1000 replicates) are written below. Support values ≤70 are not shown.

In evaluating the relationships among Dsh genes across animals, we included known Dsh homologs from organisms for which we did not perform whole-genome analyses (*C. hemisphaerica*, *S. mediterranea*, and *X. tropicalis*). We found that each of the three vertebrate Dsh orthologs shares ancestry and that the planarian *S. mediterranea* and the nematode *T. spiralis* Dshs (*Smed-dvl-1*, *Smed-dvl-2*, and *Tspi-dsh-1*) are not very similar to other Dsh homologs (Figure 4). The rest of the nematode Dsh orthologs in this analysis formed two distinct clades, with the *mig-5* orthologs forming one clade, and the *dsh-1* orthologs forming the other (Figure 4). Although we did not find any proteins with significant similarity to *Cele-dsh-2* in our broad clustering analysis, the phylogenetic analyses place it in the clade containing all nematode *dsh-1* orthologs (Figure 4). These *C. elegans* paralogs, *Cele-dsh-1* and *Cele-dsh-2*, are approximately 130 kb apart on chromosome II, have the same orientation, and form a conserved gene cluster with a recombination frequency of 0.61%. Together with the phylogenetic analyses, this suggests that *Cele-dsh-2* is a diverging duplication of *Cele-dsh-1* and is specific to *C. elegans* or perhaps the *Caenorhabditis* lineage.

To evaluate the apparent expansion of Dsh among caenorhabditids and determine the origin of *dsh-2*, we performed a cluster analysis of whole caenorhabditid proteomes, including *C. angaria*, *C. japonica*, *C. elegans*, *C. brenneri*, *C. remanei*, and *C. briggsae*. The relationships among *Caenorhabditis* nematodes are becoming increasingly refined as more species are described (Kiontke *et al.* 2011). Our analysis includes members of both the *Elegans* and *Drosophilae* supergroups within the *Caenorhabditis* genus, and resulted in three unsurprising clusters of Dsh genes: *dsh-1*, *dsh-2*, and *mig-5*. We found that all caenorhabditids have *dsh-1* and *mig-5* orthologs but that *C. angaria* lacks a *dsh-2* ortholog, suggesting that only members of the *Elegans* supergroup (*C. japonica*, *C. elegans*, *C. brenneri*, *C. remanei*, and *C. briggsae*) have *dsh-2* orthologs. Furthermore, this clustering analysis revealed the possibility of species-specific expansions. For example, *C. angaria* appeared to have two potential *dsh-1* orthologs, *C. japonica* appeared to have two *dsh-1* orthologs and two *dsh-2* orthologs, and *C. brenneri* appeared to have two orthologs each of *dsh-1*, *dsh-2*, and *mig-5*. Detailed protein analyses of this kind rely on the quality of the assemblies and gene predictions of the proteomes used, and the results can often improve the annotations. Despite valiant efforts to inbreed these nematodes before genomic sequencing, the current assemblies of *C. brenneri*, *C. remanei*, and *C. japonica* (WormBase release WS225) are known to have considerable heterozygosity, with some genes being represented by allelic variants (Barrière *et al.* 2009). Furthermore, the genome assembly for *C. angaria* is still quite fragmented (Mortazavi *et al.* 2010), although additional sequencing is ongoing. We explore these genes in more detail in the following section.

The gene relationships among the *Caenorhabditis* Dsh paralogs is consistent with those recovered using a broader sampling of animal taxa (Figure 4 and Supporting Information, Figure S1): we recapitulate three clades, one for each of the three paralogs with *dsh-1* and *dsh-2* being more closely related, supporting the notion that *dsh-2* is the result of a fairly recent duplication event and has subsequently diverged from *dsh-1*. This duplication could have occurred after the split between the *Elegans* and *Drosophilae* supergroups, or may have occurred earlier and been subsequently lost in *C. angaria*. More could be inferred about the evolution of Dsh among caenorhabditids from sequencing additional taxa from this genus.

### Conservation and diversification of Dsh domain architecture

**Figure 5:**
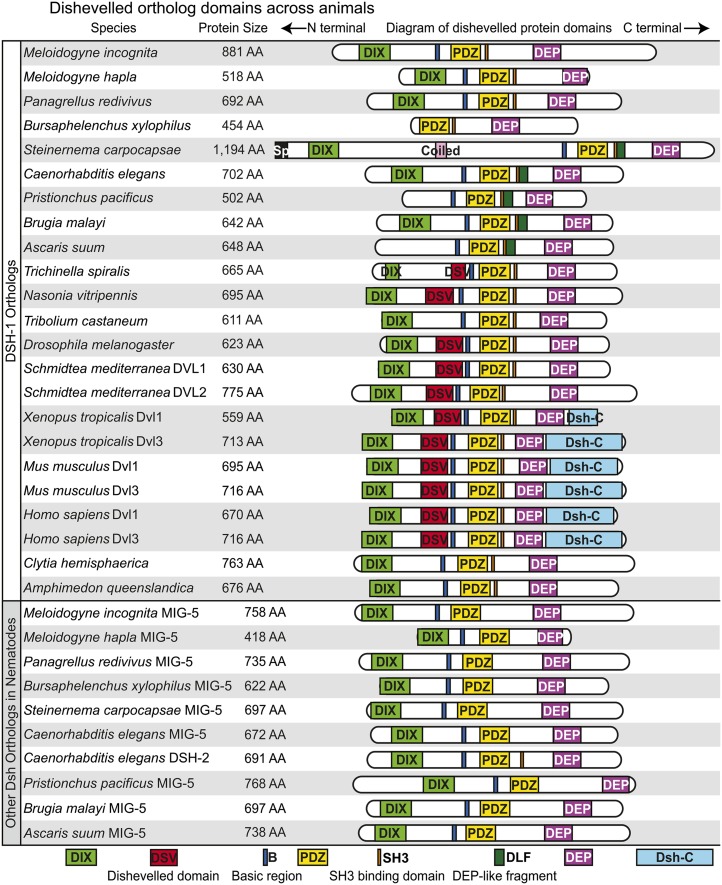
Schematic diagram of Dsh orthologs in selected animal species. Proteins and their domains are drawn in proportion to the number of amino acids they contain.

**Figure 6:**
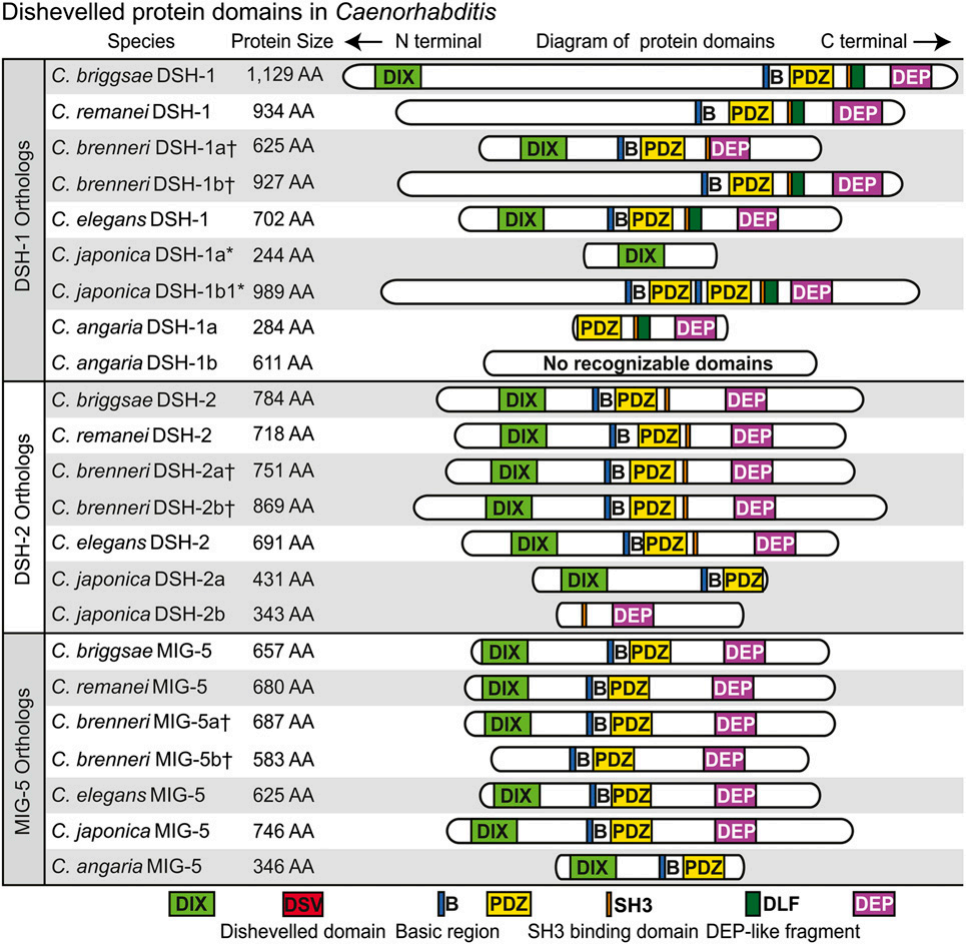
Schematic diagram of Dsh orthologs in sequenced *Caenorhabditis* species. Proteins and their domains are drawn in proportion to the number of amino acids they contain. †These *C. brenneri* proteins are thought to be splice isoforms or perhaps allelic variants and not paralogous duplicates. *Cjap-DSH-1a and Cjap-DSH-1b although presently annotated as separate genes, we suggest they are fragments of the same protein rather than two different orthologs of DSH-1. This is not the case with Cjap-DSH-2a and Cjap-DSH-2b, which likely are separate proteins.

Next, we wanted to assess the protein domains in Dsh and evaluate the conservation of domain structure across animal evolution among orthologs and paralogs. The SMART database recognizes the DIX, DSV, PDZ, and DEP domains as being approximately 80, 72, 80, and 75 amino acids, respectively, with some variation between species, particularly in the DIX and DSV domains (Figures 2, 5, and 6). The basic region located between DIX and PDZ, as well as the proline-rich region containing an SH3 binding domain located between PDZ and DEP are not recognized by SMART, but they were identifiable by sequence alignment similarity with known sequences (Penton *et al.* 2002).

We found the PDZ and DEP domains to be the most highly conserved structural components of Dsh across taxa, being present in all taxa from the sponge *A. queenslandica* to mammals (Figure 5 and File S1). The basic region, just anterior to the PDZ domain, is also highly conserved and only absent from the *Bxyl-dsh-1* ortholog, which is truncated. The proline-rich region extends over an approximately 20 amino acid window and contains a class I core SH3 binding motif RxEPV/IR/QP (where x stands for any amino acid), with ligand preference varying around the PxxP core (Penton *et al.* 2002). Although the proline-rich region is not always conserved, the SH3 binding domain is conserved in the *dsh-1* orthologs of all taxa, but is absent in nematode *mig-5* orthologs (Figure 5, Figure 6, Figure S2, and Figure S3). We refer to this region as the SH3 binding motif rather than the proline-rich region due to the conservation of the motif across taxa although the area surrounding the motif is not necessarily proline-rich in some nematode taxa. The DIX domain is conserved in nearly all Dsh orthologs but is conspicuously missing from two nematode *dsh-1* orthologs; *Ppac-dsh-1* and *Asuu-dsh-1* (Figure 5). The understudied DSV domain appears to have experienced dynamic evolution, being absent from both sponge and jellyfish taxa and arising in bilaterian taxa (Figure 1, Figure 5, and File S1). The DSV domain is conserved in planaria, vertebrates, and two of the three insect taxa we investigated (*Dmel-dsh* and *Nvit-dsh*) but is missing from *Tcas-dsh* and is absent from all nematode Dsh homologs except *Tspi-dsh-1*, the only Dsh homolog in the most basal nematode lineage included in our analysis (Figure 3) (Holterman *et al.* 2006). The Dsh-C domain is vertebrate specific but appears to be truncated in *Xtrop-Dvl1* (Figure 5). We found a previously unreported DEP-like fragment (DLF) domain, recognized by the SMART database, and is present and conserved in several nematode species from clades 8, 9, and at least one species, *S. carpocapsae*, from nematode clade 10 (Figures 3 and 5). The amino acid sequence conservation and codon variation that we detect both in the DSV and DLF domains suggest that these are functionally relevant, despite the current lack of functional data (Figure S2, Figure S3, and File S1). The absence of a recognizable DSV domain in early branching lineages (*i.e.*, *A. queenslandica* and *C. hemisphaerica*), and its apparent loss in *T. castaneum* and all evaluated nematode lineages branching after clade 2 suggest that its conservation among some insects, planarians, and vertebrates has functional significance and should be tested. Similarly, the conservation of DLF among clades 8, 9, and at least one clade 10 nematode (*S. carpocapsae*), suggest that it too has functional significance.

The domain architecture of Dsh orthologs within the *Caenorhabditis* genus is more dynamic than that observed across a broader sampling of animals, likely facilitated by the presence of three Dsh orthologs (Figures 5 and 6). The PDZ and DEP domains are the most highly conserved across caenorhabditid orthologs, with both only being absent from *Cjap-dsh-1a* and *Cang-dsh-1b*. The DEP domain is missing from *Cang-mig-5*, and PDZ and DEP are separated between the *Cjap-dsh-2a* and *Cjap-dsh-2b* (Figure 6 and Figure S3). The basic region is also highly conserved and is present in all orthologs with protein sequence N-terminal to the PDZ domain (Figure 6). The SH3 binding motif is conserved in *dsh-1* and *dsh-2* orthologs that contain protein sequence C-terminal to the PDZ and/or N-terminal to the DEP domain, but is entirely absent from all *mig-5* orthologs (Figure 6 and Figure S3). The newly discovered DLF domain, where present, is between the PDZ and DEP domains, just C-terminal to the SH3 binding motif. The DLF domain is only present in nematode *dsh-1* orthologs, and is present in all nematode *dsh-1* orthologs that have PDZ and DEP domains except *Cbre-dsh-1a*, where it is conspicuously missing.

**Figure 7:**
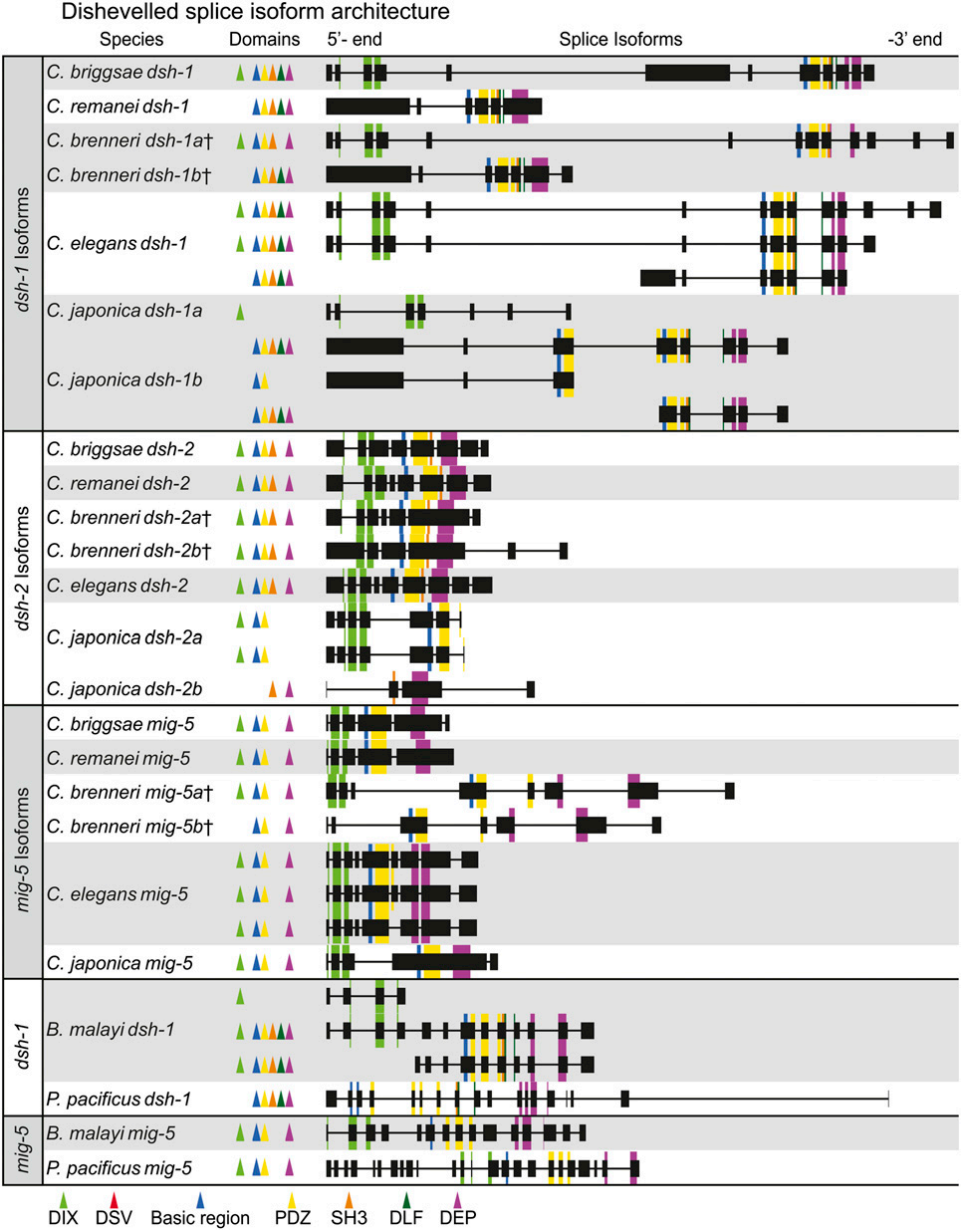
The known Dsh splice isoform architecture for *dsh-1*, *dsh-2*, and *mig-5* among caenorhabditids. All features (exons, introns, and domains) are drawn in proportion to the number of nucleotides they contain. All isoforms are shown in the same orientation, regardless of their actual orientation in their respective genomes. The known isoforms of *Bmal-dsh-1*, *Bmal-mig-5*, *Ppac-dsh-1*, and *Ppac-mig-5* are included at the bottom for outgroup comparison.

We investigated the splice isoforms of all three Dsh paralogs within *Caenorhabditis* and used *B. malayi* and *P. pacificus* for outgroup comparison (Figure 7). There is conserved isoform architecture among these species for all three paralogs, although no species has been as thoroughly studied as *C. elegans*, which has three isoforms of *Cele-dsh-1* as well as *Cele-mig-5* (Figure 7). For example, all *dsh-1* isoforms that have a DIX domain have it split across three exons, whereas the PDZ domain appears to be split across two exons in all caenorabditid taxa except *C. japonica*, where it might be split across three. In addition to partitioning domains among proteins, as *P. pacificus* seems to have done with the DIX domain being present in *Ppac-dsh-1* and absent from *Ppac-mig-5*, other taxa can produce isoforms with and without certain domains (*e.g.*, *Cele-dsh-1* and *Bmal-dsh-1*; Figure 7). Too little is known about splice isoforms in the other species to draw strong conclusions from these data, but interesting features of conservation and divergence are apparent. Additionally, this analysis sheds light on the potential paralogs identified within *C. japonica* and *C. brenneri*. *Cjap-dsh-1a* and *Cjap-dsh-1b* are tandem in the same orientation, with *Cjap-dsh-1a* being <3 kb upstream from *Cjap-dsh-1b*, suggesting that these are fragments of the same gene (Figures 6 and 7). However, *Cjap-dsh-2a* and *Cjap-dsh-2b*, although still in the same orientation, are >10 kb apart with *Cjap-dsh-2b*, which has the DEP domain, being upstream of *Cjap-dsh-2b*, inverting the traditional order of DIX, PDZ, and then DEP, suggesting that these might actually be separate genes, representing a physical partitioning of DIX, PDZ, and the basic region on one protein and the SH3 binding motif and DEP on the other (Figures 6 and 7). The potential paralogs within *C. brenneri* (*Cbre-dsh-1a*, *Cbre-dsh-1b*, *Cbre-dsh-2a*, *Cbre-dsh-2b*, *Cbre-mig-5a*, and *Cbre-mig-5b*) were each on separate contigs, offering no potential clarification. However, a nucleotide alignment of the PDZ and DEP domains revealed that each paralogous pair has identical nucleotide sequence, suggesting that these are likely allelic variants or splice isoforms (Barrière *et al.* 2009). Splice isoforms seem particularly likely in cases in which a paralogous pair differ in domain content (*e.g.*, *Cbre-dsh-1a*, *Cbre-dsh-1b*, and *Cbre-mig-5a*, and *Cbre-mig-5b*).

The amino acid sequence alignments of Dsh across animals and across caenorhabditids show clear regions of high conservation and other regions with considerable divergence. Across animals, we detect at least two codons that are experiencing strong negative selection (codons 318 and 335) and at least 10 codons that are experiencing diversifying selection (codons 6, 221, 222, 234, 235, 238, 239, 252, 284, and 354; Figure S2). Focusing on caenorhabditids, we detect at least three codons experiencing negative selection (codons 113, 252, and 261) and at least four codons that are experiencing diversifying selection (codons 63, 85, 155, and 193; Figure S3). It is not surprising that areas of functional significance are highly conserved across species, whereas those regions that show considerable divergence or are experiencing diversifying selection may play important roles in the acquisition of novel functions but remain to be functionally tested.

### Nuclear transport

In addition to the conserved elements of Dsh shown in Figure 2, there are other motifs, structural components, and phosphorylation sites that affect the function of Dsh. For example, the presence of a nuclear export signal (NES) and a nuclear localization signal (NLS) affect the subcellular distribution of Dsh. A conserved NES has been identified as M/LxxLxL, where mutations in the leucines lead to nuclear localization of Dsh in *Xenopus* (Itoh *et al.* 2005). We found this NES to have patchy conservation, being present in *Aque-Dvl*, *Cint-Dvl*, and all vertebrate Dsh orthologs, except *Xtrop-Dvl1* (Figure S2). It was not present in *Chem-Dvl* or any insect or nematode Dsh orthologs (Figure S2); however, it was present in *Smed-dvl-2* but absent from *Smed-dvl-1*. Previous studies indicate that Dsh translocates to the nucleus and is actively exported into the cytoplasm, presumably via NLS and NES signals and that blocking the nuclear export by mutating the NES or chemically inhibiting nuclear export leads to nuclear localization of Dsh in vertebrates (Itoh *et al.* 2005; Torres and Nelson 2000). A NLS sequence was previously identified in vertebrates, flies, and *Hydra*, and identified as IxLT/VAK (Itoh *et al.* 2005). We found this NLS to be highly conserved across the taxa in our analyses, being present in the Dsh orthologs of most taxa we examined, but absent in all nematode *mig-5* orthologs, and identifiable yet slightly altered in *dsh-2* orthologs (Figure S2 and Figure S3). This NLS has been shown to be necessary and sufficient for nuclear translocation of Dsh in vertebrates, although this has not been pursued in invertebrate taxa (Itoh *et al.* 2005; Torres and Nelson 2000).

### Phosphorylation of tyrosine 473

The phosphorylation of *Dmel-dsh* tyrosine 473 (Y473), located in the DEP domain, is essential for PCP signaling (Yanfeng *et al.* 2011). The substitution of *Dmel-dsh* Y473 to phenylalanine (Dsh^Y473F^) leads to strong PCP specific defects in *Drosophila* but has no effect on canonical Wnt signaling. It is believed that this site in the DEP domain is phosphorylated by an Abelson family tyrosine kinase (Abl), which is also required for PCP signaling but not canonical Wnt signaling (Singh *et al.* 2010). We found that Y473 is conserved across all evaluated Dsh orthologs except *Mhap-mig-5* and *Minc-mig-5* as well as *Smed-dvl-1* (Figure S2 and Figure S3). All evaluated organisms have at least one Dsh with a conserved Y473, implying an ancient and essential function across Metazoa, and suggesting another potential mechanism for partitioning the function of Dsh paralogs and/or splice isoforms.

## Discussion

**Figure 8:**
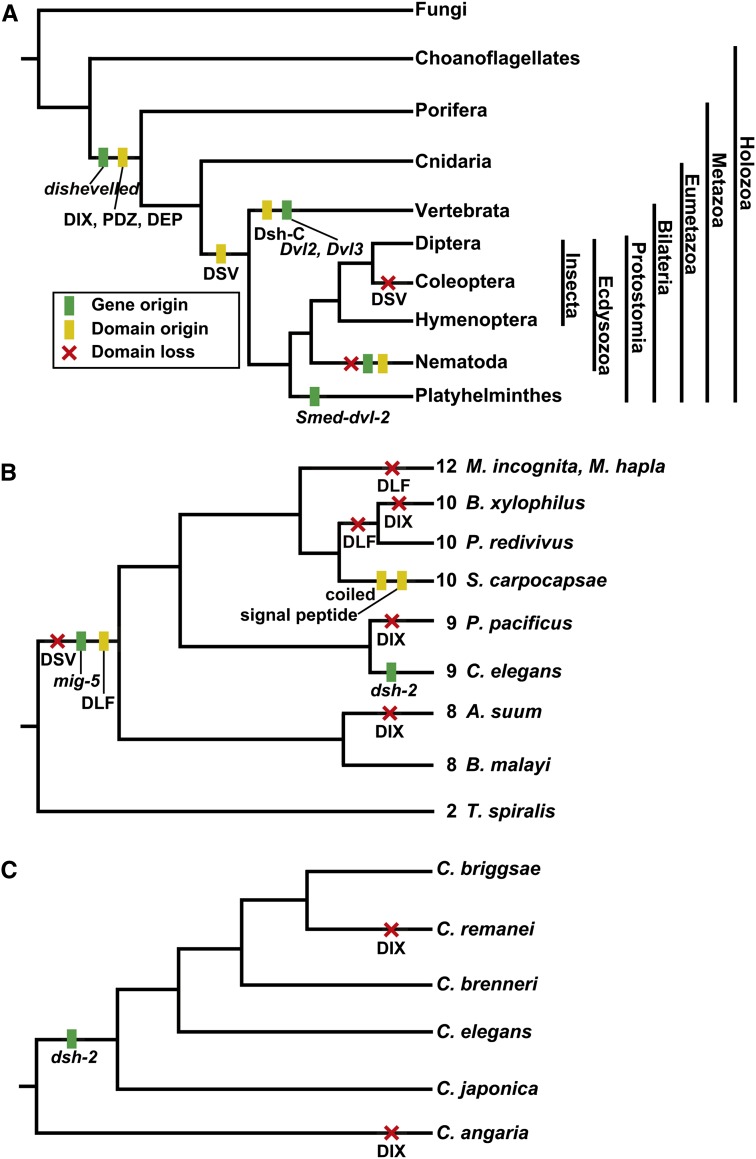
Graphical summary of events during the evolution of Dsh mapped onto cladograms. (A) A cladogram of animal evolution with important features of Dsh evolution mapped onto it. (B) Cladogram of nematodes with identified features of Dsh evolution mapped onto it. (C) Cladogram of caenorhabditids with identified features of Dsh evolution mapped onto it.

The origin of Dsh lies in the common ancestor of Metazoa and likely had the three major functional domains DIX, PDZ, and DEP (Figure 8A). Dsh has experienced dynamic evolution across animal evolution, acquiring new domains and experiencing duplications in several animal lineages. The DSV domain seems to have evolved prior to the bilaterian split and been subsequently lost in some nematode and insect taxa. In no phylum where multiple taxa were examined did we find complete conservation of both domain architecture and number of Dsh orthologs. We have identified many structural features that are conserved and others that are divergent or lineage-specific. These features suggest potential mechanisms for partitioning the various functions of Dsh among isoforms and/or paralogs. We discuss these findings in the context of known functional specializations among invertebrates.

### Dishevelled across nematodes

Nematoda is an ancient animal lineage, originating during the Precambrian or Cambrian explosion more than 500 million years ago (Ayala *et al.* 1998; Rodriguez-Trelles *et al.* 2002). With this abundance of evolutionary time, nematodes have evolved to inhabit virtually every habitat known and nearly every ecological niche. The model nematode *C. elegans* was the first metazoan to have its genome sequenced and is among the most studied and best understood animals on earth (*C. elegans* Sequencing Consortium 1998). Often what is learned about *C. elegans* is assumed to be conserved among nematodes, and although this may be largely true for some features, *e.g.*, neuroanatomy and CO_2_ detection and response (Bumbarger *et al.* 2007, 2009; Hallem *et al.* 2011a,b; Hallem and Sternberg 2008; Ragsdale *et al.* 2009), *C. elegans* is a derived nematode with many unique features (Blaxter 1998, 2011). We have shown that the number of Dsh homologs varies across nematodes, at least from one to three, but many taxa remain unstudied, especially within the basal clades of the phylum (Figure 8B). Most genera in our study have two Dsh homologs, *dsh-1* and *mig-5*. The acquisition of *mig-5* is ancient, occurring sometime after the split of clade 2 and before the split of clade 8, although additional taxon sampling would improve this estimate (Figure 8B). The *C. elegans* genome encodes three Dsh genes, *Cele-dsh-1*, *Cele-dsh-2*, and *Cele-mig-5*. We have shown that *dsh-2* is likely a paralog of *dsh-1* and a derived character among *Caenorhabditis* species, perhaps only among members of the *Elegans* supergroup (Figure 8C). We identified only one Dsh ortholog in *T. spiralis*, *Tspi-dsh-1*, and find that among nematodes, it has unique similarity to insect Dsh as it is the only nematode Dsh known to have a DSV domain (Figures 5 and 6).

The domain architecture among nematode Dshs is variable and suggests potential mechanisms of functional divergence. We have discovered a novel Dep-like fragment domain that is present and highly conserved in half of the 10 nematode taxa we examined (Figures 5, 6, and 8B). The domain architecture of *mig-5* is conserved, having the same structural features (DIX, PDZ, DEP, and the basic region) in all taxa (except *Cang-mig-5*, which is missing DEP), while *dsh-1* orthologs are more diverse (Figures 5 and 6). *Asuu-dsh-1* and *Ppac-dsh-1* lack the DIX domain, *Bxyl-dsh-1* lacks the DIX domain and the basic region while *Scar-dsh-1* seems to have acquired a signal peptide and a coiled domain that is unknown in any other Dsh homologs. Finally, we detected a conserved NLS in all nematode *dsh-1* orthologs (and both *Cbre-dsh-2* orthologs), suggesting that these proteins may be translocated to the nucleus, as has been shown in vertebrates. It is worth noting that the presence of an NLS and a basic region, features that are broadly conserved in Dsh orthologs across animals, are hallmarks of transcription factors, although this possibility has not been experimentally explored (Grove *et al.* 2009).

Although there are many examples in *C. elegans* of the functional overlap of Dsh paralogs, there are also known specializations for each. For example, B-cell polarity in males is controlled by Wnt signaling, where *Cele-mig-5* defective males have altered B-cell daughter size (Herman *et al.* 1995; Sawa *et al.* 1996; Wu and Herman 2006). Neither *Cele-dsh-1* nor *Cele-dsh-2* affects the polarity of the B cell as single mutants and neither enhances the phenotype of the *Cele-mig-5* mutant, showing specialization of *Cele-mig-5* in this pathway (Wu and Herman 2006). The divergence of Dsh function in *C*. elegans can also be seen in the outgrowth of neurites from RME head motor neurons. In this pathway, Cele-DSH-1 physically interacts with Ror/CAM-1 to transmit the Wnt/CWN-2 signal to downstream components enabling neurite outgrowth (Song *et al.* 2010). The binding activity of Cele-DSH-1 to Ror/CAM-1 lies in its PDZ and DEP domains, whereas the DIX domain is not required for binding. Furthermore, only *Cele-dsh-1b*, the isoform that lacks the DIX domain (Figure 7; *Cele-dsh-1b* is the *Cele-dsh-1* ‘b’ isoform from WormBase), was shown to express in the RME cells, and Cele-DSH-1b is sufficient to rescue the *dsh-1* null phenotype, suggesting that alternative splicing of Dsh can lead to functional specialization within *C. elegans* (Song *et al.* 2010). An example of domain specialization within a Dsh homolog can be seen in the asymmetric cell division of the *C. elegans* ABpl/rpppa neuroblast via a β-catenin independent pathway (Hingwing *et al.* 2009). Domain analysis has shown that the DIX domain is not required for ABpl/rpppa asymmetric division but that the DEP domain is essential. Hingwing *et al.* (Hingwing *et al.* 2009) go on to show that *Cele-dsh-2* is involved in the asymmetric divisions of SGP cells along the proximal-distal axis of the developing gonad, which leads to the formation of distal tip cells from distal daughters and an anchor or ventral uterine cell from the proximal daughters. Loss of *Cele-dsh-2* results in two proximal daughters. Unlike the asymmetric division of the ABpl/rpppa neuroblast, both the DIX and DEP domains are essential for proper SGP cell division, thus demonstrating the divergent functional roles of domains in a single Dsh ortholog (Hingwing *et al.* 2009).

We have identified and shown the conservation of a Dep-like fragment domain across all *Caenorhabditis dsh-1* orthologs along with *Asuu-dsh-1*, *Bmal-dsh-1*, *Ppac-dsh-1*, and *Scar-dsh-1*. Furthermore, we have shown that the basic region, DIX, PDZ, DEP, SH3 binding motif, and the NLS are conserved across nematode *dsh-1* orthologs (with a few exceptions lacking the DIX domain and the absence of the basic region in *Bxyl-dsh-1*). We have shown the extreme structural conservation of all *mig-5* orthologs, and that these uniformly lack the SH3 binding motif as well as the NLS. The functional relevance of these features and what role, if any, they play in the partitioning of Dsh function would be interesting to explore. These results suggest, for example, that *Ppac-dsh-1* and *Ppac-mig-5* might have evolved to function in separate pathways and perform at least some non-overlapping functions (Figure 5). The apparent lack of an NES in any nematode or insect Dsh is also striking, especially considering the presence of an NLS among most *dsh-1* orthologs. Perhaps nematodes and insects have an alternative and as yet unidentified NES, since these proteins are not reported to be nuclear-specific.

### Dishevelled in other invertebrates

*S. mediterranea* has two orthologs of Dsh, *Smed-dvl-1* and *Smed-dvl-2* (Gurley *et al.* 2008). Initial studies in this flatworm have investigated functional specialization of these paralogs. Only *Smed-dvl-2* appears to be involved in β-catenin-dependent signaling: phenotypes described after silencing canonical Wnt ligands are reproduced upon the silencing of *Smed-dvl-2* (Almuedo-Castillo *et al.* 2011). Conversely, both *Smed-dvl-1* and *Smed-dvl-2* transduce the noncanonical signals that control neural connectivity as well as mediolateral patterning of the central nervous system, neither of which involves components of the PCP pathway. Components of the PCP pathway, including Van Gogh and Diversin, have been implicated in the apical positioning of the basal body in epithelial cells. Interestingly, only *Smed-dvl-2* has been shown to function alongside these core PCP components. Our domain analysis of planarian Dsh supports these experimental results. Only *Smed-dvl-2* contains the NLS and NES sequences, implying its role in β-catenin−dependent signaling. Because *Smed-dvl-1* lacks both sequences, we would suggest that it cannot function in a β-catenin−dependent pathway, and this hypothesis is supported experimentally (Almuedo-Castillo *et al.* 2011). Furthermore, it has been shown that tyrosine473 is essential for PCP signaling. This amino acid is present in *Smed-dvl-2*, but not *Smed-dvl-1*, supporting the experimental finding that only *Smed-dvl-2* can function in the PCP pathway (Almuedo-Castillo *et al.* 2011).

Insects have only one copy of Dsh, at least the taxa that have been investigated so far. Significant effort has gone into understanding how *Drosophila*, with one Dsh ortholog, channels a Wnt signal into distinct pathways. It has been shown that specificity is achieved by the presence or absence of binding partners as well as the subcellular localization of Dsh (Wallingford and Habas 2005). Other work has shown that qualitatively different Fz-Dsh interactions underlie PCP and canonical Wnt signaling (Strutt *et al.* 2012).

The insect Dshs, *Dmel-dsh*, *Tcas-dsh*, and *Nvit-dsh*, are very similar in architecture. All have a DIX, PDZ, and DEP domain as well as the basic region and SH3 binding motif. *Tcas-dsh* is the only one that lacks a DSV domain, but this suggests that additional taxon sampling could reveal a broader trend. Interestingly, all insect Dsh proteins have a NLS, but none have the known NES that has been shown in *Xenopus*. It is currently not known whether invertebrate Dsh translocates to the nucleus, but if Dsh does, it must employ a different export signal than the one found in *Xenopus*. More work must be done to better understand its localization and transport in invertebrates.

We have discussed the origin and evolution of Dsh in a variety of metazoan lineages, emphasizing a recurring theme of Dsh duplication and expansion in many phyla. The data we have evaluated suggest that Dsh arose in the most recent common ancestor of Metazoa and possessed many of the structural features that have come to characterize Dsh (Figure 2). Most basal lineages within explored phyla appear to have only a single Dsh ortholog, leading us to conclude that the ancestral state of Wnt signaling pathways was built using a single Dsh protein acting as the hub, and has then experienced lineage-specific expansions in many phyla. The deuterostome taxa wherein Dsh has been explored reveal that early branching deuterostome phyla (*e.g.*, Echinodermata and Hemichordata) have only one Dsh ortholog, which is also true of basal chordate lineages like lancelets and sea squirts (Cephalochordata and Urochordata respectively) (Gray *et al.* 2009). It is noteworthy that as more taxa in a particular phylum are explored, the derived lineages seem to have convergently evolved multiple Dsh orthologs, although there may be exceptions such as insects, where even the more recent lineages seem to use the ancestral strategy of partitioning Dsh function in ways other than protein duplication and subsequent divergence.

As the hub of Wnt signaling, Dsh plays an essential role in animal development and homeostasis. We have shown that Dsh has experienced dynamic evolution across Metazoa, including the acquisition and loss of domains as well as gene duplication in many lineages. Our findings on the divergent and varied architecture of Dsh across taxa provide testable hypotheses about the means of these specializations. The dynamic evolution of Dsh among nematodes both by paralogous duplication and the formation of lineage-specific splice isoforms raises questions of protein evolution and provides clues as to how these organisms have dealt with the complex intricacies of having a protein, like Dsh, act as a central messenger hub connected to so many different and vitally important pathways.

## Supplementary Material

Supporting Information

## References

[bib1] Adamska M. Degnan S. M. Green K. M. Adamski M. Craigie A., 2007 Wnt and TGF-beta expression in the sponge Amphimedon queenslandica and the origin of metazoan embryonic patterning. PLoS ONE 2: e1031 17925879 10.1371/journal.pone.0001031 PMC2000352

[bib2] Adamska M. Larroux C. Adamski M. Green K. Lovas E., 2010 Structure and expression of conserved Wnt pathway components in the demosponge Amphimedon queenslandica. Evol. Dev. 12: 494–518 20883218 10.1111/j.1525-142X.2010.00435.x

[bib3] Almuedo-Castillo M. Salo E. Adell T., 2011 Dishevelled is essential for neural connectivity and planar cell polarity in planarians. Proc. Natl. Acad. Sci. USA 108: 2813–2818 21282632 10.1073/pnas.1012090108 PMC3041082

[bib4] Ayala F. J. Rzhetsky A. Ayala F. J., 1998 Origin of the metazoan phyla: molecular clocks confirm paleontological estimates. Proc. Natl. Acad. Sci. USA 95: 606–611 9435239 10.1073/pnas.95.2.606 PMC18467

[bib5] Barrière A. Yang S.-P. Pekarek E. Thomas C. G. Haag E. S., 2009 Detecting heterozygosity in shotgun genome assemblies: Lessons from obligately outcrossing nematodes. Genome Res. 19: 470–480 19204328 10.1101/gr.081851.108 PMC2661809

[bib6] Blaxter M., 1998 *Caenorhabditis elegans* is a nematode. Science 282: 2041–2046 9851921 10.1126/science.282.5396.2041

[bib7] Blaxter M., 2011 Nematodes: The worm and its relatives. PLoS Biol. 9: 1–9 10.1371/journal.pbio.1001050 PMC3079589 21526226

[bib8] Blaxter M. L. De Ley P. Garey J. R. Liu L. X. Scheldeman P., 1998 A molecular evolutionary framework for the phylum Nematoda. Nature 392: 71–75 9510248 10.1038/32160

[bib9] Boutros M. Mlodzik M., 1999 Dishevelled: at the crossroads of divergent intracellular signaling pathways. Mech. Dev. 83: 27–37 10507837 10.1016/s0925-4773(99)00046-5

[bib10] Bumbarger D. J. Crum J. Ellisman M. H. Baldwin J. G., 2007 Three-dimensional fine structural reconstruction of the nose sensory structures of Acrobeles complexus compared to Caenorhabditis elegans (Nematoda: Rhabditida). J. Morphol. 268: 649–663 17514723 10.1002/jmor.10535

[bib11] Bumbarger D. J. Wijeratne S. Carter C. Crum J. Ellisman M. H., 2009 Three-dimensional reconstruction of the amphid sensilla in the microbial feeding nematode, Acrobeles complexus (Nematoda: Rhabditida). J. Comp. Neurol. 512: 271–281 19003904 10.1002/cne.21882 PMC2750866

[bib12] *C. elegans* Sequencing Consortium, 1998 Genome sequence of the nematode C. elegans: A platform for investigating biology. Science 282: 2012–2018 9851916 10.1126/science.282.5396.2012

[bib13] Darriba D. Taboada G. L. Doallo R. Posada D., 2012 jModelTest 2: more models, new heuristics and parallel computing. Nat. Methods 9: 772 22847109 10.1038/nmeth.2109 PMC4594756

[bib14] Dillman A. R. Mortazavi A. Sternberg P. W., 2012 Incorporating genomics into the toolkit of nematology. J. Nematol. 44: 191–205 23482088 PMC3578471

[bib15] Edgar R. C., 2004 MUSCLE: a multiple sequence alignment method with reduced time and space complexity. BMC Bioinformatics 5: 113 15318951 10.1186/1471-2105-5-113 PMC517706

[bib16] Etheridge S. L. Ray S. Li S. Hamblet N. S. Lijam N., 2008 Murine dishevelled 3 functions in redundant pathways with dishevelled 1 and 2 in normal cardiac outflow tract, cochlea, and neural tube development. PLoS Genet. 4: e1000259 19008950 10.1371/journal.pgen.1000259 PMC2576453

[bib17] Fahmy O. G. Fahmy M., 1959 New mutants report. Dros. Inf. Serv. 33: 82–94

[bib18] Gao C. Chen Y. G., 2010 Dishevelled: The hub of Wnt signaling. Cell. Signal. 22: 717–727 20006983 10.1016/j.cellsig.2009.11.021

[bib19] Goloboff P. A., 1999 Analyzing large data sets in reasonable times: solutions for composite optima. Cladistics-the International Journal of the Willi Hennig Society 15: 415–428 10.1111/j.1096-0031.1999.tb00278.x 34902941

[bib20] Gray R. S. Bayly R. D. Green S. A. Agarwala S. Lowe C. J., 2009 Diversification of the expression patterns and developmental functions of the dishevelled gene family during chordate evolution. Dev. Dyn. 238: 2044–2057 19618470 10.1002/dvdy.22028 PMC2782374

[bib21] Grove C. A. De Masi F. Barrasa M. I. Newburger D. E. Alkema M. J., 2009 A multiparameter network reveals extensive divergence between *C. elegans* bHLH transcription factors. Cell 138: 314–327 19632181 10.1016/j.cell.2009.04.058 PMC2774807

[bib22] Guindon S. Gascuel O., 2003 A simple, fast, and accurate algorithm to estimate large phylogenies by maximum likelihood. Syst. Biol. 52: 696–704 14530136 10.1080/10635150390235520

[bib23] Gurley K. A. Rink J. C. Sanchez Alvarado A., 2008 Beta-catenin defines head *vs.* tail identity during planarian regeneration and homeostasis. Science 319: 323–327 18063757 10.1126/science.1150029 PMC2755502

[bib24] Hallem E. A. Sternberg P. W., 2008 Acute carbon dioxide avoidance in *Caenorhabditis elegans*. Proc. Natl. Acad. Sci. USA 105: 8038–8043 18524955 10.1073/pnas.0707469105 PMC2430355

[bib25] Hallem E. A. Dillman A. R. Hong A. V. Zhang Y. Yano J. M., 2011 a A sensory code for host seeking in parasitic nematodes. Curr. Biol. 21: 377–383 21353558 10.1016/j.cub.2011.01.048 PMC3152378

[bib26] Hallem E. A. Spencer W. C. McWhirter R. D. Zeller G. Henz S. R., 2011 b Receptor-type guanylate cyclase is required for carbon dioxide sensation by *Caenorhabditis elegans*. Proc. Natl. Acad. Sci. USA 108: 254–259 21173231 10.1073/pnas.1017354108 PMC3017194

[bib27] Hamblet N. S. Lijam N. Ruiz-Lozano P. Wang J. B. Yang Y. S., 2002 Dishevelled 2 is essential for cardiac outflow tract development, somite segmentation and neural tube closure. Development 129: 5827–5838 12421720 10.1242/dev.00164

[bib28] Herman M. A. Vassilieva L. L. Horvitz H. R. Shaw J. E. Herman R. K., 1995 The *C. elegans* gene lin-44, which controls the polarity of certain asymmetric cell divisions, encodes a Wnt protein and acts cell nonautonomously. Cell 83: 101–110 7553861 10.1016/0092-8674(95)90238-4

[bib29] Hingwing K. Lee S. Nykilchuk L. Walston T. Hardin J., 2009 CWN-1 functions with DSH-2 to regulate *C. elegans* asymmetric neuroblast division in a beta-catenin independent Wnt pathway. Dev. Biol. 328: 245–256 19389360 10.1016/j.ydbio.2009.01.025

[bib30] Holstein T. W., 2012 The evolution of the Wnt pathway. Cold Spring Harb. Perspect. Biol. 4: a007922 22751150 10.1101/cshperspect.a007922 PMC3385961

[bib31] Holterman M. van der Wurff A. van den Elsen S. van Megen H. Bongers T., 2006 Phylum-wide analysis of SSU rDNA reveals deep phylogenetic relationships among nematodes and accelerated evolution toward crown clades. Mol. Biol. Evol. 23: 1792–1800 16790472 10.1093/molbev/msl044

[bib32] Itoh K. Brott B. K. Bae G. U. Ratcliffe M. J. Sokol S. Y., 2005 Nuclear localization is required for Dishevelled function in Wnt/beta-catenin signaling. J. Biol. 4: 3 15720724 10.1186/jbiol20 PMC551520

[bib33] Kiontke K. C. Felix M. A. Ailion M. Rockman M. V. Braendle C., 2011 A phylogeny and molecular barcodes for Caenorhabditis, with numerous new species from rotting fruits. BMC Evol. Biol. 11: 339 22103856 10.1186/1471-2148-11-339 PMC3277298

[bib34] Klingensmith J. Nusse R. Perrimon N., 1994 The Drosophila segment polarity gene dishevelled encodes a novel protein required for response to the wingless signal. Genes Dev. 8: 118–130 8288125 10.1101/gad.8.1.118

[bib35] Kusserow A. Pang K. Sturm C. Hrouda M. Lentfer J., 2005 Unexpected complexity of the Wnt gene family in a sea anemone. Nature 433: 156–160 15650739 10.1038/nature03158

[bib36] Letunic I. Doerks T. Bork P., 2012 SMART 7: recent updates to the protein domain annotation resource. Nucleic Acids Res. 40: D302–D305 22053084 10.1093/nar/gkr931 PMC3245027

[bib37] Li L. Stoeckert C. J. Roos D. S., 2003 OrthoMCL: identification of ortholog groups for eukaryotic genomes. Genome Res. 13: 2178–2189 12952885 10.1101/gr.1224503 PMC403725

[bib38] Lijam N. Paylor R. McDonald M. P. Crawley J. N. Deng C. X., 1997 Social interaction and sensorimotor gating abnormalities in mice lacking Dvl1. Cell 90: 895–905 9298901 10.1016/s0092-8674(00)80354-2

[bib39] Luo J. Chen J. Deng Z. L. Luo X. Song W. X., 2007 Wnt signaling and human diseases: what are the therapeutic implications? Lab. Invest. 87: 97–103 17211410 10.1038/labinvest.3700509

[bib40] Monsoro-Burq A. H. Wang E. Harland R., 2005 Msx1 and Pax3 cooperate to mediate FGF8 and WNT signals during Xenopus neural crest induction. Dev. Cell 8: 167–178 15691759 10.1016/j.devcel.2004.12.017

[bib41] Mortazavi A. Schwarz E. M. Williams B. A. Schaeffer L. Antoshechkin I., 2010 Scaffolding a *Caenorhabditis* nematode genome with RNA-seq. Genome Res. 20: 1740–1747 20980554 10.1101/gr.111021.110 PMC2990000

[bib42] Murrell B. Wertheim J. O. Moola S. Weighill T. Scheffler K., 2012 Detecting individual sites subject to episodic diversifying selection. PLoS Genet. 8: e1002764 22807683 10.1371/journal.pgen.1002764 PMC3395634

[bib43] Nicholas K. B. Nicholas H. B. Deerfield D. W., 1997 GeneDoc: analysis and visualization of genetic variation. EMBnet.news 4: 1–4

[bib44] Nixon K. C., 1999 The Parsimony Ratchet, a new method for rapid parsimony analysis. Cladistics 15: 407–414 10.1111/j.1096-0031.1999.tb00277.x 34902938

[bib45] Penton A. Wodarz A. Nusse R., 2002 A mutational analysis of dishevelled in Drosophila defines novel domains in the dishevelled protein as well as novel suppressing alleles of axin. Genetics 161: 747–762 12072470 10.1093/genetics/161.2.747 PMC1462152

[bib46] Ragsdale E. J. Ngo P. T. Crum J. Ellisman M. H. Baldwin J. G., 2009 Comparative, three-dimensional anterior sensory reconstruction of *Aphelenchus avenae* (nematoda: Tylenchomorpha). J. Comp. Neurol. 517: 616–632 19824103 10.1002/cne.22170

[bib47] Rodriguez-Trelles F. Tarrio R. Ayala F. J., 2002 A methodological bias toward overestimation of molecular evolutionary time scales. Proc. Natl. Acad. Sci. USA 99: 8112–8115 12060757 10.1073/pnas.122231299 PMC123029

[bib48] Ruvkun G. Hobert O., 1998 The taxonomy of developmental control in *Caenorhabditis elegans*. Science 282: 2033–2041 9851920 10.1126/science.282.5396.2033

[bib49] Sawa H. Lobel L. Horvitz H. R., 1996 The *Caenorhabditis elegans* gene lin-17, which is required for certain asymmetric cell divisions, encodes a putative seven-transmembrane protein similar to the Drosophila frizzled protein. Genes Dev. 10: 2189–2197 8804313 10.1101/gad.10.17.2189

[bib50] Seifert J. R. Mlodzik M., 2007 Frizzled/PCP signalling: a conserved mechanism regulating cell polarity and directed motility. Nat. Rev. Genet. 8: 126–138 17230199 10.1038/nrg2042

[bib51] Simons M. Mlodzik M., 2008 Planar cell polarity signaling: from fly development to human disease. Annu. Rev. Genet. 42: 517–540 18710302 10.1146/annurev.genet.42.110807.091432 PMC2814158

[bib52] Singh J. Yanfeng W. A. Grumolato L. Aaronson S. A. Mlodzik M., 2010 Abelson family kinases regulate Frizzled planar cell polarity signaling via Dsh phosphorylation. Genes Dev. 24: 2157–2168 20837657 10.1101/gad.1961010 PMC2947768

[bib53] Song S. Zhang B. Sun H. Li X. Xiang Y., 2010 A Wnt-Frz/Ror-Dsh pathway regulates neurite outgrowth in Caenorhabditis elegans. PLoS Genet. 12: 6 10.1371/journal.pgen.1001056 PMC2920835 20711352

[bib66] Srinivasan J. Dillman A. R. Macchietto M. G. Heikkinen L. Lakso M., 2013 The draft genome and transcriptome of *Panagrellus redivivus* are shaped by the harsh demands of a free-living lifestyle. Genetics (in press) 10.1534/genetics.112.148809 PMC3606103 23410827

[bib54] Stamatakis A. Hoover P. Rougemont J., 2008 A rapid bootstrap algorithm for the RAxML Web servers. Syst. Biol. 57: 758–771 18853362 10.1080/10635150802429642

[bib55] Strutt D. Madder D. Chaudhary V. Artymiuk P. J., 2012 Structure-function dissection of the frizzled receptor in *Drosophila melanogaster* suggests different mechanisms of action in planar polarity and canonical Wnt signaling. Genetics. 10.1534/genetics.112.144592 PMC3512140 23023003

[bib56] Sugimura R. He X. C. Venkatraman A. Arai F. Box A., 2012 Noncanonical Wnt signaling maintains hematopoietic stem cells in the niche. Cell 150: 351–365 22817897 10.1016/j.cell.2012.05.041 PMC4492542

[bib57] Sweetman D. Wagstaff L. Cooper O. Weijer C. Munsterberg A., 2008 The migration of paraxial and lateral plate mesoderm cells emerging from the late primitive streak is controlled by different Wnt signals. BMC Dev. Biol. 8: 63 18541012 10.1186/1471-213X-8-63 PMC2435575

[bib58] Tamura K. Peterson D. Peterson N. Stecher G. Nei M., 2011 MEGA5: molecular evolutionary genetics analysis using maximum likelihood, evolutionary distance, and maximum parsimony methods. Mol. Biol. Evol. 28: 2731–2739 21546353 10.1093/molbev/msr121 PMC3203626

[bib59] Torres M. A. Nelson W. J., 2000 Colocalization and redistribution of dishevelled and actin during Wnt-induced mesenchymal morphogenesis. J. Cell Biol. 149: 1433–1442 10871283 10.1083/jcb.149.7.1433 PMC2175133

[bib60] Tauriello D. V. Jordens I. Kirchner K. Slootstra J. W. Kruitwagen T., 2012 Wnt/beta-catenin signaling requires interaction of the Dishevelled DEP domain and C terminus with a discontinuous motif in Frizzled. Proc. Natl. Acad. Sci. USA 109: E812–E820 22411803 10.1073/pnas.1114802109 PMC3325702

[bib61] Wallingford J. B. Habas R., 2005 The developmental biology of Dishevelled: an enigmatic protein governing cell fate and cell polarity. Development 132: 4421–4436 16192308 10.1242/dev.02068

[bib62] Wernersson R. Pedersen A. G., 2003 RevTrans: multiple alignment of coding DNA from aligned amino acid sequences. Nucleic Acids Res. 31: 3537–3539 12824361 10.1093/nar/gkg609 PMC169015

[bib63] Wharton K. A. Jr, 2003 Runnin’ with the Dvl: proteins that associate with Dsh/Dvl and their significance to Wnt signal transduction. Dev. Biol. 253: 1–17 12490194 10.1006/dbio.2002.0869

[bib64] Wu M. Herman M. A., 2006 A novel noncanonical Wnt pathway is involved in the regulation of the asymmetric B cell division in C. elegans. Dev. Biol. 293: 316–329 16631156 10.1016/j.ydbio.2005.12.024

[bib65] Yanfeng W. A. Berhane H. Mola M. Singh J. Jenny A., 2011 Functional dissection of phosphorylation of Disheveled in Drosophila. Dev. Biol. 360: 132–142 21963539 10.1016/j.ydbio.2011.09.017 PMC3221411

