# Supplementary material for: Origin and Evolution of Dishevelled
Source: G3 (Bethesda). 2013 Feb 1;3(2):251–62. doi: 10.1534/g3.112.005314 (PMC3564985; doi:10.1534/g3.112.005314)
Supplement: Supporting Information [file supp_3_2_251__index.html]

Supporting Information 

# Origin and Evolution of Dishevelled

## Supporting Information for Dillman *et al.*, 2013

**Files in this Data Supplement:**

- Supporting Information - Figures S1-S3 and File S1 (PDF, 658 KB)
- Figure S1 - Phylogenetic analysis of Dsh orthologs across caenorhabditids based on the protein coding nucleotide alignment from the N-terminus of the PDZ domain through the C-terminus of the DEP domain (PDF, 152 KB)
- Figure S2 - Protein alignment from the beginning of the PDZ domain through the end of the DEP domain across animals (PDF, 372 KB)
- Figure S3 - Protein alignment from the beginning of the PDZ domain through the end of the DEP domain from all caenorhabditids in this analysis, plus *N. vitripennis* and *T. spiralis* as outgoups (PDF, 234 KB)
- File S1 - All Dsh proteins identified and used in this analysis (PDF, 160 KB)
