# Supplementary material for: Origin and Evolution of Dishevelled
Source: G3 (Bethesda). 2013 Feb 1;3(2):251–62. doi: 10.1534/g3.112.005314 (PMC3564985; doi:10.1534/g3.112.005314)
Supplement: Supporting Information [file supp_3.2.251_005314SI.pdf]

G3 Genes | Genomes | Genetics

Origin and Evolution of Dishevelled

Adler R. Dillman[†], Paul J. Minor[†], and Paul W. Sternberg[1]

[*]Howard Hughes Medical Institute, Division of Biology, California Institute of Technology, Pasadena, CA 91125, USA

[†]These authors contributed equally to this work

[1]Corresponding author

Paul W. Sternberg
Mail Code 156-29
California Institute of Technology
1200 E. California Blvd.
Pasadena, CA 91125
(626) 395-3990
pws@caltech.edu

DOI: 10.1534/g3.112.005314

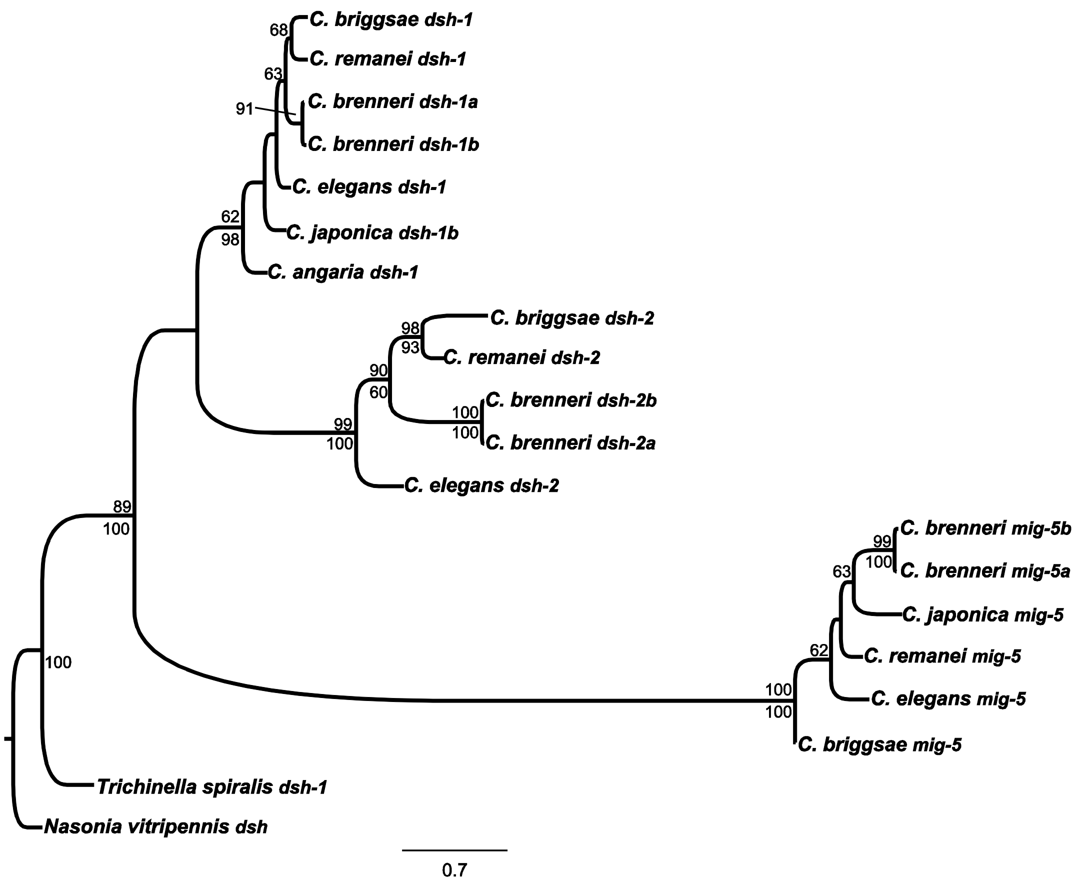

Figure S1 Phylogenetic analysis of Dsh orthologs across caenorhabditids based on the protein coding nucleotide alignment from the N-terminus of the PDZ domain through the C-terminus of the DEP domain. The ML tree (rooted with the outgroup taxon *N. vitripennis*) is shown. For each node, ML bootstrap support values (1,000 replicates) are above the nodes while parsimony bootstrap values (1,000 replicates) are written below. Support values <u><</u>70 are not shown.

Figure S2A

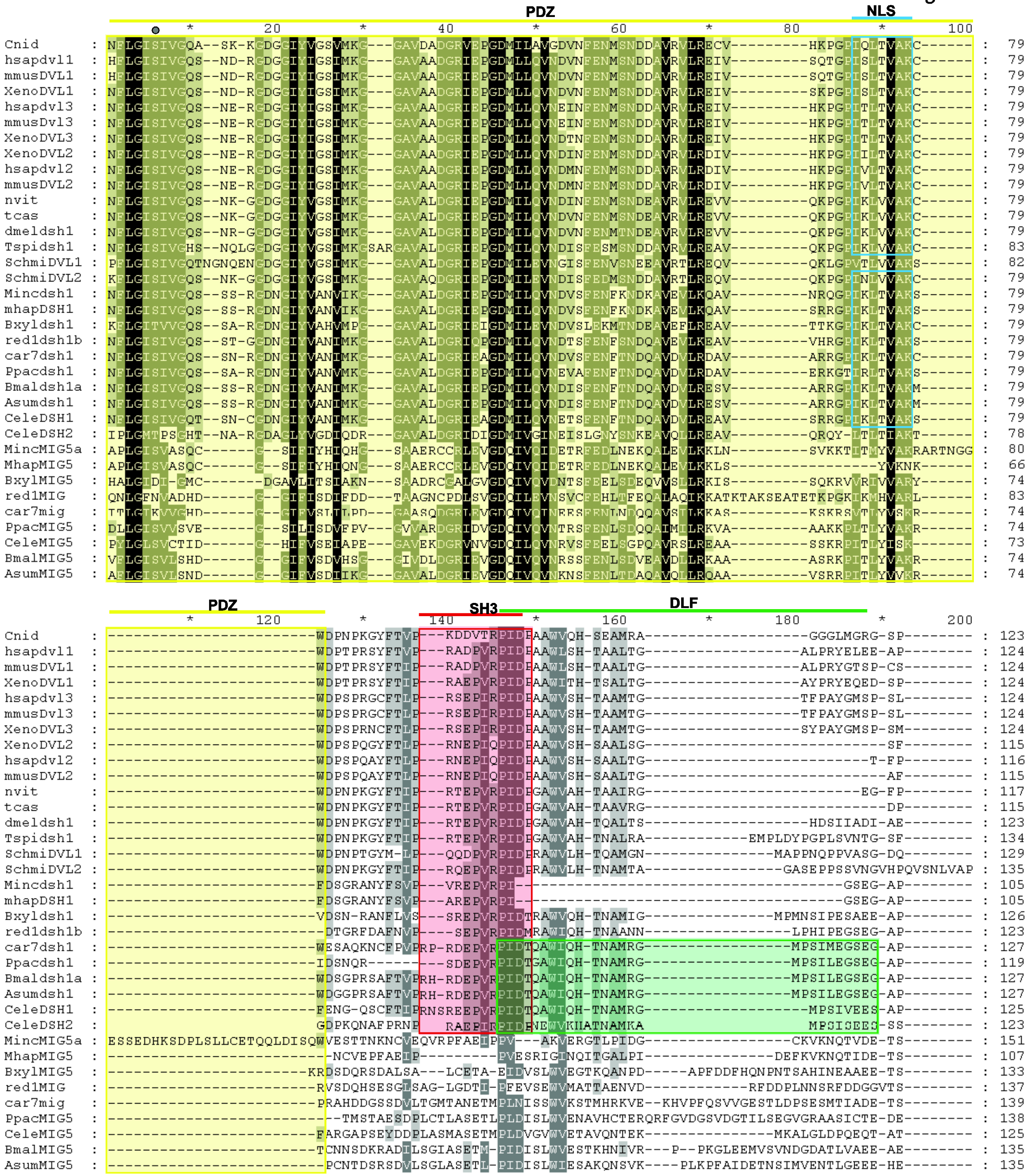
PDZ
NLS
SH3
DLF
Cnid
hsapdvl1
mmusDVL1
XenoDVL1
hsapdvl3
mmusDvl3
XenoDVL3
XenoDVL2
hsapdvl2
mmusDVL2
nvit
tcas
dmeldsh1
Tspidsh1
SchmiDVL1
SchmiDVL2
Mincdsh1
mhapDSH1
Bxyldsh1
red1dsh1b
car7dsh1
Ppacdsh1
Bmaldsh1a
Asumdsh1
CeleDSH1
CeleDSH2
MincMIG5a
MhapMIG5
BxylMIG5
red1MIG
car7mig
PpacMIG5
CeleMIG5
BmalMIG5
AsumMIG5

Figure S2B

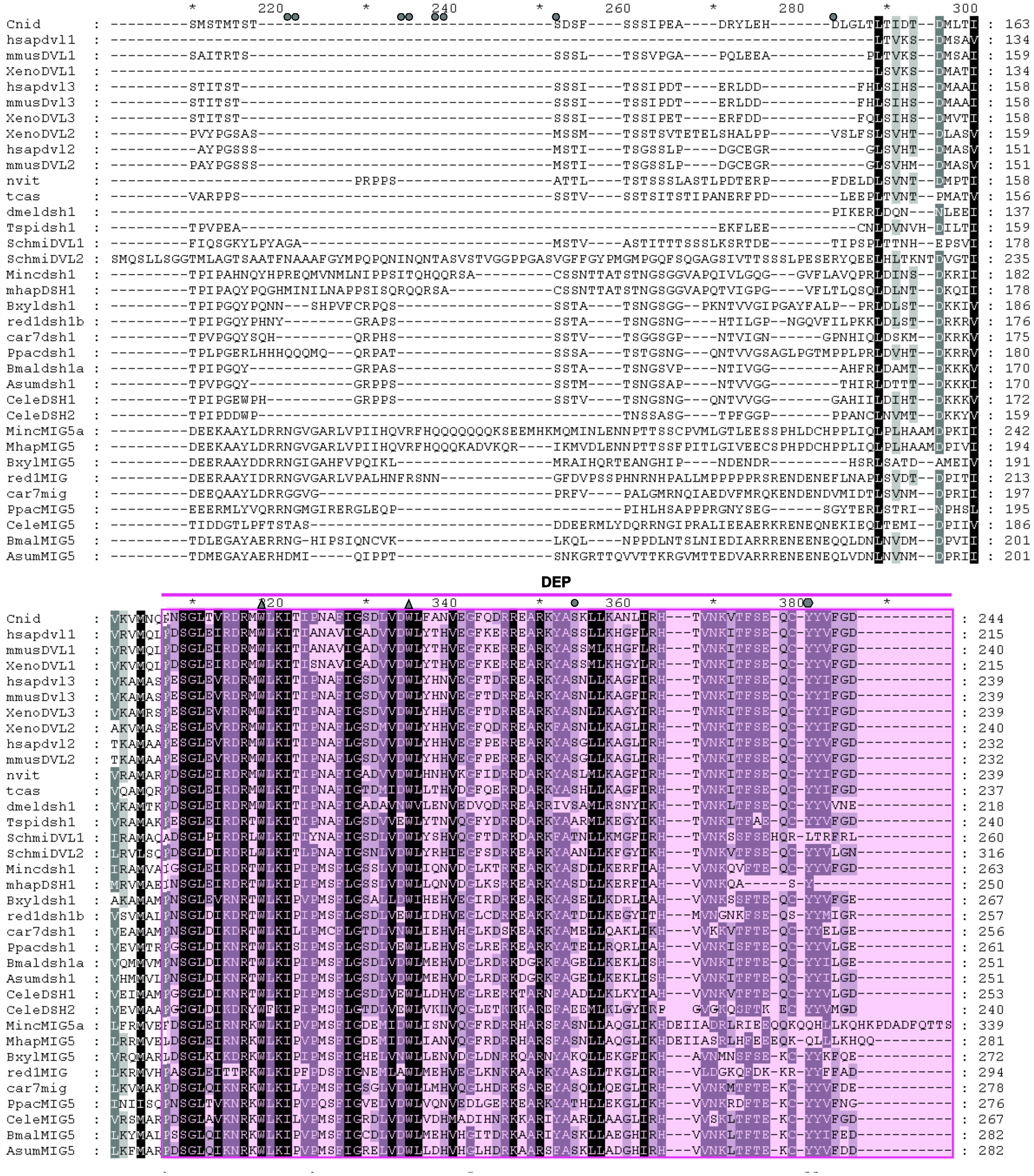

Figure S2 Protein alignment from the beginning of the PDZ domain through the end of the DEP domain across animals. (A) is the first half of the alignment and (B) is the second half. A nucleotide version of this alignment was used to generate the phylogenetic tree from figure 3. Domain features are highlighted in color and labeled, including PDZ, NLS, SH3, DLF, and DEP. The conserved tyrosine 473 (Y473) is labeled with a polygon (⬣). Codons identified to be under negative selection are labeled with a triangle (▲) while codons identified as experiencing diversifying selection are labeled with a circle (●).

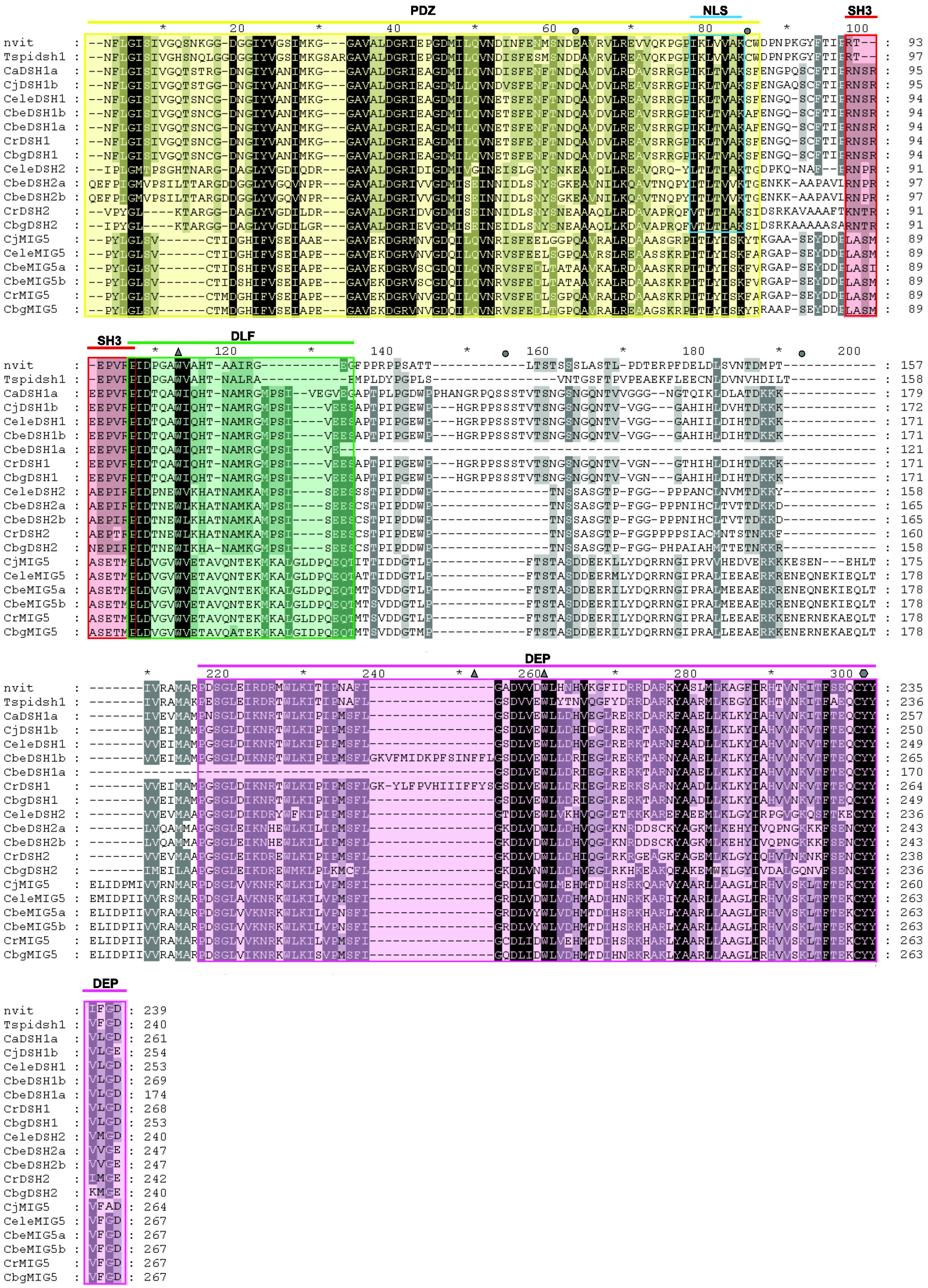

Figure S3 Protein alignment from the beginning of the PDZ domain through the end of the DEP domain from all caenorhabditids in this analysis, plus *N. vitripennis* and *T. spiralis* as outgoups. A nucleotide version of this alignment was used to generate the phylogenetic tree from figure S1. Domain features are highlighted in color and labeled, including PDZ, NLS, SH3, DLF, and DEP. The conserved tyrosine 473 (Y473) is labeled with a polygon (⬣). Codons identified to be under negative selection are labeled with a triangle (▲) while codons identified as experiencing diversifying selection are labeled with a circle (●).

File S1

All Dsh proteins identified and used in this analysis

File S1 is available for download at http://www.g3journal.org/lookup/suppl/doi:10.1534/g3.112.005314/-/DC1.
