# Supplementary material for: Origin and Evolution of Dishevelled
Source: G3 (Bethesda). 2013 Feb 1;3(2):251–62. doi: 10.1534/g3.112.005314 (PMC3564985; doi:10.1534/g3.112.005314)
Supplement: Supporting Information [file supp_3.2.251_FigureS1.pdf]

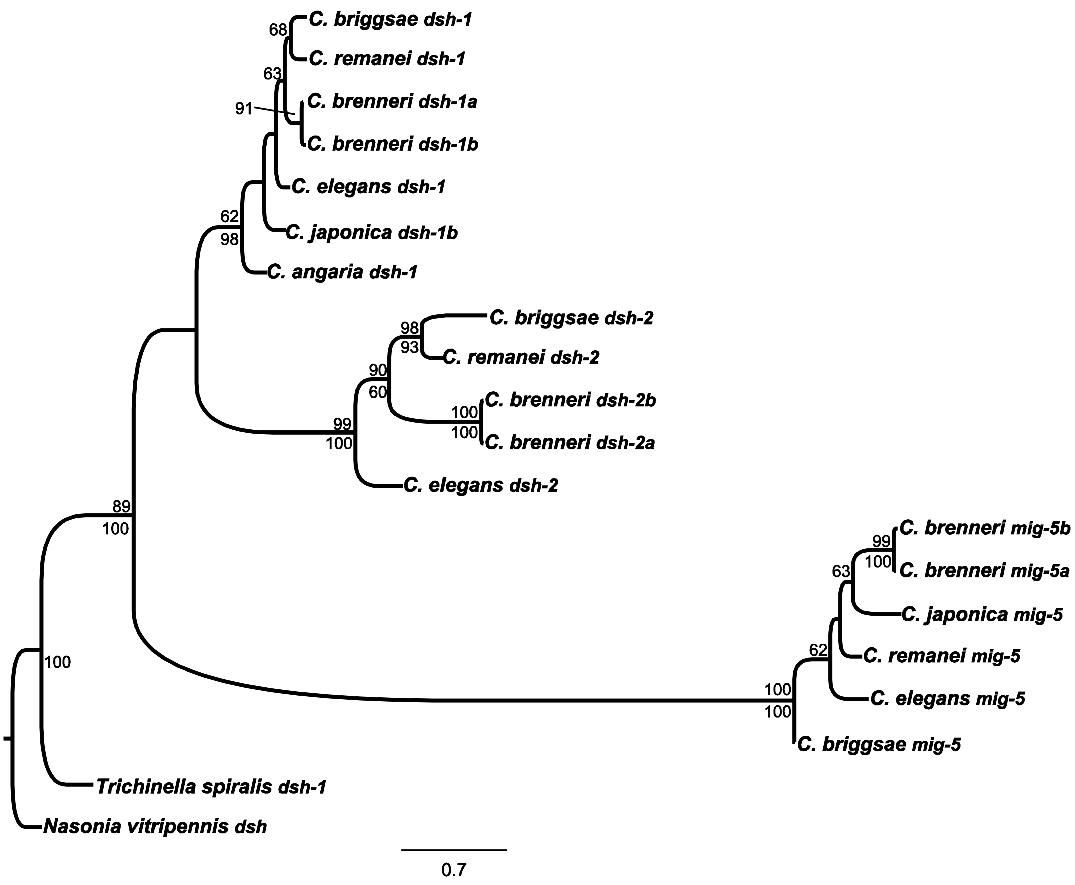

Figure S1 Phylogenetic analysis of Dsh orthologs across caenorhabditids based on the protein coding nucleotide alignment from the N-terminus of the PDZ domain through the C-terminus of the DEP domain. The ML tree (rooted with the outgroup taxon *N. vitripennis*) is shown. For each node, ML bootstrap support values (1,000 replicates) are above the nodes while parsimony bootstrap values (1,000 replicates) are written below. Support values $\leq$70 are not shown.
