# Supplementary material for: Origin and Evolution of Dishevelled
Source: G3 (Bethesda). 2013 Feb 1;3(2):251–62. doi: 10.1534/g3.112.005314 (PMC3564985; doi:10.1534/g3.112.005314)
Supplement: Supporting Information [file supp_3.2.251_FigureS2.pdf]

Figure S2A

```
                                                       PDZ                                          NLS
                  o    *        20         *        40         *        60         *        80         *        100
Cnid       : NFLGISIVGQA--SK-KGDGGIYVGSVMKG---GAVDADGRVEPGDMILAVGDVNFENMSNDDAVRVLRECV---------HKPGPIQLTVAKC------ :  79
hsapdvl1   : HFLGISIVGQS--ND-RGDGGIYIGSIMKG---GAVAADGRIEPGDMLLQVNDVNFENMSNDDAVRVLREIV---------SQTGPISLTVAKC------ :  79
mmusDVL1   : HFLGISIVGQS--ND-RGDGGIYIGSIMKG---GAVAADGRIEPGDMLLQVNDVNFENMSNDDAVRVLREIV---------SQTGPISLTVAKC------ :  79
XenoDVL1   : NFLGISIVGQS--ND-RGDGGIYIGSIMKG---GAVAADGRIEPGDMLLQVNDVNFENMSNDDAVRVLREIV---------SKPGPISLTVAKC------ :  79
hsapdvl3   : NFLGISIVGQS--NE-RGDGGIYIGSIMKG---GAVAADGRIEPGDMLLQVNEINFENMSNDDAVRVLREIV---------HKPGPITLTVAKC------ :  79
mmusDvl3   : NFLGISIVGQS--NE-RGDGGIYIGSIMKG---GAVAADGRIEPGDMLLQVNEINFENMSNDDAVRVLREIV---------HKPGPITLTVAKC------ :  79
XenoDVL3   : NFLGISIVGQS--NE-RGDGGIYIGSIMKG---GAVAADGRIEPGDMLLQVNDTNFENMSNDDAVRVLREIV---------HKPGPITLTVAKC------ :  79
XenoDVL2   : NFLGISIVGQS--NE-RGDGGIYIGSIMKG---GAVAADGRIEPGDMLLQVNDINFENMSNDDAVRVLRDIV---------HKPGPIILTVAKC------ :  79
hsapdvl2   : NFLGISIVGQS--NE-RGDGGIYIGSIMKG---GAVAADGRIEPGDMLLQVNDMNFENMSNDDAVRVLRDIV---------HKPGPIVLTVAKC------ :  79
mmusDVL2   : NFLGISIVGQS--NE-RGDGGIYIGSIMKG---GAVAADGRIEPGDMLLQVNDMNFENMSNDDAVRVLRDIV---------HKPGPIVLTVAKC------ :  79
nvit       : NFLGISIVGQS--NK-GGDGGIYVGSIMKG---GAVALDGRIEPGDMILQVNDINFENMSNDEAVRVLREVV---------QKPGPIKLVVAKC------ :  79
tcas       : NFLGISIVGQS--NK-GGDGGIYVGSIMKG---GAVALDGRIEPGDMILQVNDVNFENMSNDEAVRVLREVV---------QKPGPIKLVVAKC------ :  79
dmeldsh1   : NFLGISIVGQS--NR-GGDGGIYVGSIMKG---GAVALDGRIEPGDMILQVNDVNFENMTNDEAVRVLREVV---------QKPGPIKLVVAKC------ :  79
Tspidsh1   : NFLGISIVGHS--NQLGGDGGIYVGSIMKGSARGAVALDGRIEPGDMILQVNDISFESMSNDDAVRVLREAV---------QKPGPIKLVVAKC------ :  83
SchmiDVL1  : PFLGISIVGQTNGNQENGDGGIYVGSIMKG---GAVALDGRIEPGDMILEVNGISFENVSNEEAVRTLREQV---------QKLGPVTLVVAKS------ :  82
SchmiDVL2  : KFLGISIVGQS--NK-GGDGGIYVGSIMKG---GAVAQDGRIEPGDMILEVNDISFEDMSNDDAVRTLREQV---------QKPGPINLVVAKC------ :  79
Mincdsh1   : NFLGISIVGQS--SS-RGDNGIYVANVIKG---GAVALDGRIEPGDMILQVNDVSFENFKNDKAVEVLKQAV---------NRQGPIKLTVAKS------ :  79
mhapDSH1   : NFLGISIVGQS--SS-RGDNGIYVANVIKG---GAVALDGRIEPGDMILQVNDVSFENFKNDKAVEVLKQAV---------SRRGPIKLTVAKS------ :  79
Bxyldsh1   : KFLGITVVGQS--SA-RGDNGIYVAHVMPG---GAVALDGRIEIGDMILEVNDVSLEKMTNDEAVEFLREAV---------TTKGPIKLTVAKC------ :  79
red1dsh1b  : NFLGISIVGQS--ST-GGDNGIYVANIMKG---GAVALDGRIQPGDMILQVNDTSFENFSNDQAVEVLKEAV---------VHRGPIKLTVAKS------ :  79
car7dsh1   : NFLGISIVGQS--SN-RGDNGIYVANIMKG---GAVALDGRIEAGDMILQVNDVSFENFTNDQAVDVLRDAV---------ARRGPIKLTVAKC------ :  79
Ppacdsh1   : NFLGISIVGQS--SA-RGDNGIYVANVMKG---GAVALDGRIEPGDMILQVNEVAFENFTNDQAVDVLRDAV---------ERKGTIRLTVAKS------ :  79
Bmaldsh1a  : NFLGISIVGQS--SS-RGDNGIYVANIMKG---GAVALDGRIEPGDMILQVNDISFENFTNDQAVDVLRESV---------ARRGPIKLTVAKM------ :  79
Asumdsh1   : NFLGISIVGQS--SS-RGDNGIYVANIMKG---GAVALDGRIEPGDMILQVNDISFENFTNDQAVDVLRESV---------ARRGPIKLTVAKM------ :  79
CeleDSH1   : NFLGISIVGQT--SN-CGDNGIYVANIMKG---GAVALDGRIEAGDMILQVNETSFENFTNDQAVDVLREAV---------SRRGPIKLTVAKS------ :  79
CeleDSH2   : IPLGMTPSGHT--NA-RGDAGLYVGDIQDR---GAVALDGRIDIGDMIVGINEISLGNYSNKEAVQLLREAV---------QRQY-LTLTIAKT------ :  78
MincMIG5a  : APLGISVASQC------G--SIFIYHIQHG---SAAERCCRLEVGDQIVQIDETRFEDLNEKQALEVLKKLN---------SVKKTITMYVAKRARTNGG :  80
MhapMIG5   : APLGISVASQC------G--SIFIYHIQNG---SAAERCCRLEVGDQIVQIDETRFEDLNEKQALEVLKKLS-----------------YVKNK------ :  66
BxylMIG5   : HALGIDI-GMC-------DGAVLITSIAKN---SAADRCGALGVGDQIVQVDNTSFEELSDEQVVSLLRKIS---------SQKRVVRIVVARY------ :  74
red1MIG    : QNLGFNVADHD------G--GIFISDIFDD---TAAGNCPDLSVGDQILEVNSVCFEHLTFEQALAQIKKATKTAKSEATETKPGKIKMHVARL------ :  83
car7mig    : TTLGTKVVGHD------G--GTFVSLTLPD---GAASQDGRLEVGDQTVQTNEESFENLNDQQAVSTLKKAS---------KSKRSVTLYVSKR------ :  74
PpacMIG5   : DLLGISVVSVE------G--SILISDVFPV---GVVARDGRIDVGDQIVQVNTRSFENLSDQQAIMILRKVA---------AAKKPLTLYVAKR------ :  74
CeleMIG5   : PYLGLSVCTID------G--HIFVSEIAPE---GAVEKDGRVNVGDQILQVNRVSFEELSGPQAVRSLREAA---------SSKRPITLYISK------- :  73
BmalMIG5   : VFLGISVLSHD------G--GIFVSDVHSG---GIVDLDGRIEVGDQIVQVNRSSFENLSDVEAVDLLRKAA---------ASRKPITLYVAKR------ :  74
AsumMIG5   : AFLGISVLSND------G--GIFVSDIIKG---GAVALDGRIEVGDQIVQVNKNSFENLTDAQAVQLLRQAA---------VSRRPITLYVVKR------ :  74

                    PDZ                            SH3            DLF
                   *        120         *        140         *        160         *        180         *        200
Cnid       : ------------------------WDPNPKGYFTVP---KDDVTRPIDPAAWVQH-SEAMRA-------------------GGGLMGRG-SP-------- : 123
hsapdvl1   : ------------------------WDPTPRSYFTVP---RADPVRPIDPAAWLSH-TAALTG------------------ALPRYELEE-AP-------- : 124
mmusDVL1   : ------------------------WDPTPRSYFTIP---RADPVRPIDPAAWLSH-TAALTG------------------ALPRYGTSP-CS-------- : 124
XenoDVL1   : ------------------------WDPTPRSYFTIP---RAEPVRPIDPAAWITH-TSALTG------------------AYPRYEQED-SP-------- : 124
hsapdvl3   : ------------------------WDPSPRGCFTLP---RSEPIRPIDPAAWVSH-TAAMTG------------------TFPAYGMSP-SL-------- : 124
mmusDvl3   : ------------------------WDPSPRGCFTLP---RSEPIRPIDPAAWVSH-TAAMTG------------------TFPAYGMSP-SL-------- : 124
XenoDVL3   : ------------------------WDPSPRNCFTLP---RSEPIRPIDPAAWVSH-TAAMTG------------------SYPAYGMSP-SM-------- : 124
XenoDVL2   : ------------------------WDPSPQGYFTLP---RNEPIQPIDPAAWVSH-SAALSG----------------------------SF-------- : 115
hsapdvl2   : ------------------------WDPSPQAYFTLP---RNEPIQPIDPAAWVSH-SAALTG--------------------------T-FP-------- : 116
mmusDVL2   : ------------------------WDPSPQAYFTLP---RNEPIQPIDPAAWVSH-SAALTG----------------------------AF-------- : 115
nvit       : ------------------------WDPNPKGYFTIP---RTEPVRPIDPGAWVAH-TAAIRG-------------------------EG-FP-------- : 117
tcas       : ------------------------WDPNPKGYFTIP---RTEPVRPIDPGAWVAH-TAAVRG----------------------------DP-------- : 115
dmeldsh1   : ------------------------WDPNPKGYFTIP---RTEPVRPIDPGAWVAH-TQALTS-------------------HDSIIADI-AE-------- : 123
Tspidsh1   : ------------------------WDPNPKGYFTIP---RTEPVRPIDPGAWVAH-TNALRA------------EMPLDYPGPLSVNTG-SF-------- : 134
SchmiDVL1  : ------------------------WDPNPTGYM-LP---QQDPVRPIDPRAWVLH-TQAMGN---------------MAPPNQPPVASG-DQ-------- : 129
SchmiDVL2  : ------------------------WDPNPKGYFTIP---RQEPVRPIDPRAWVLH-TNAMTA----------------GASEPPSSVNGVHPQVSNLVAP : 135
Mincdsh1   : ------------------------FDSGRANYFSVP---VREPVRPI--------------------------------------GSEG-AP-------- : 105
mhapDSH1   : ------------------------FDSGRANYFSVP---AREPVRPI--------------------------------------GSEG-AP-------- : 105
Bxyldsh1   : ------------------------VDSN-RANFLVS---SREPVRPIDTRAWVQH-TNAMIG---------------MPMNSIPESAEE-AP-------- : 126
red1dsh1b  : -------------------------DTGRFDAFNVP----SEPVRPIDMRAWIQH-TNAANN-----------------LPHIPEGSEG-AP-------- : 123
car7dsh1   : ------------------------WESAQKNCFPVPRP-RDEPVRPIDTQAWIQH-TNAMRG-----------------MPSIMEGSEG-AP-------- : 127
Ppacdsh1   : ------------------------IDSNQR----------SDEPVRPIDTGAWIQH-TNAMRG-----------------MPSILEGSEG-AP-------- : 119
Bmaldsh1a  : ------------------------WDSGPRSAFTVPRH-RDEPVRPIDTQAWIQH-TNAMRG-----------------MPSILEGSEG-AP-------- : 127
Asumdsh1   : ------------------------WDGGPRSAFTVPRH-RDEPVRPIDTQAWIQH-TNAMRG-----------------MPSILEGSEG-AP-------- : 127
CeleDSH1   : ------------------------FENG-QSCFTIPRNSREEPVRPIDTQAWIQH-TNAMRG-------------------MPSIVEES-AP-------- : 125
CeleDSH2   :                         GDPKQNAFPRNP   RAEPIRPIDPNEWVKHATNAMKA                   MPSISEES-SS-------- : 123
MincMIG5a  : ESSEDHKSDPLSLLCETQQLDISQWVESTTNKNCVEQVRPFAEIPPV---AKVERGTLPIDG-----------------CKVKNQTVDE-TS-------- : 151
MhapMIG5   : -------------------------NCVEPFAEIP---------PVESRIGINQITGALPI---------------DEFKVKNQTIDE-TS-------- : 107
BxylMIG5   : -----------------------KRDSDQRSDALSA---LCETA-EIDVSLWVEGTKQANPD-----APFDDFHQNPNTSAHINEAAEE-TS-------- : 133
red1MIG    : ------------------------RVSDQHSESGLSAG-LGDTI-PFEVSEWVMATTAENVD------------RFDDPLNNSRFDDGGVTS-------- : 137
car7mig    : ------------------------PRAHDDGSSDVLTGMTANETMPLNISSWVKSTMHRKVE--KHVPFQSVVGESTLDPSESMTIADE-TS-------- : 139
PpacMIG5   : --------------------------TMSTAESDPLCTLASETLPLDISLWVENAVHCTERQRFGVDGSVDGTILSEGVGRAASICTE-DE-------- : 138
CeleMIG5   : ------------------------FARGAPSEYDDPLASMASETMPLDVGVWVETAVQNTEK---------------MKALGLDPQEQT-AT-------- : 125
BmalMIG5   : ------------------------TCNNSDKRADILSGIASETM-PIDISLWVESTKHNIVR---P--PKGLEEMVSVNDGDATLVAEE-AE-------- : 135
AsumMIG5   : -------------------------PCNTDSRSDVLSGLASETL-PIDISLWIESAKQNSVK----PLKPFAIDETNSIMVENTLGEEE-HE-------- : 135
```

Figure S2B

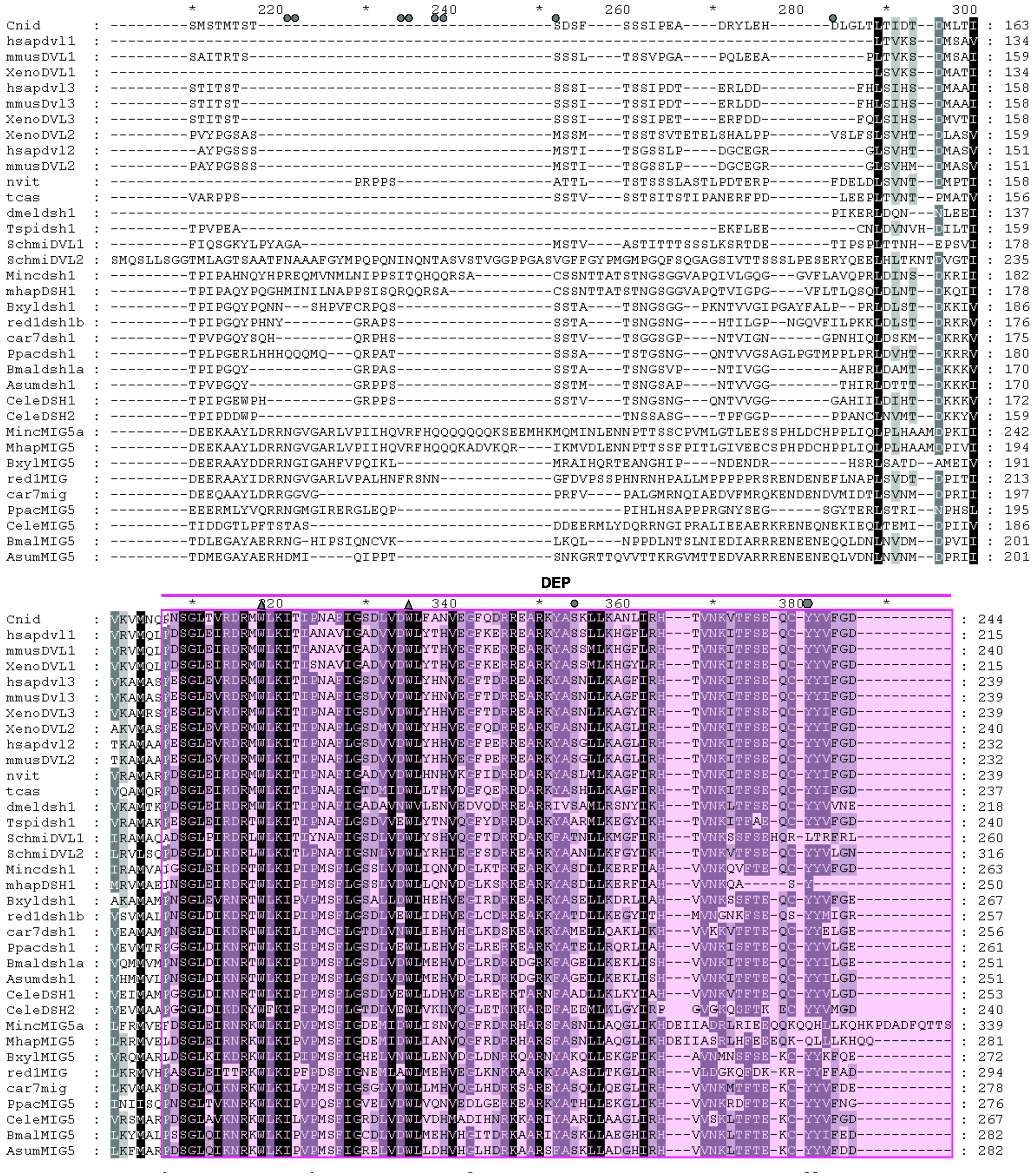

Figure S2 Protein alignment from the beginning of the PDZ domain through the end of the DEP domain across animals. (A) is the first half of the alignment and (B) is the second half. A nucleotide version of this alignment was used to generate the phylogenetic tree from figure 3. Domain features are highlighted in color and labeled, including PDZ, NLS, SH3, DLF, and DEP. The conserved tyrosine 473 (Y473) is labeled with a polygon (⬣). Codons identified to be under negative selection are labeled with a triangle (▲) while codons identified as experiencing diversifying selection are labeled with a circle (●).
