# Supplementary material for: Origin and Evolution of Dishevelled
Source: G3 (Bethesda). 2013 Feb 1;3(2):251–62. doi: 10.1534/g3.112.005314 (PMC3564985; doi:10.1534/g3.112.005314)
Supplement: Supporting Information [file supp_3.2.251_FigureS3.pdf]

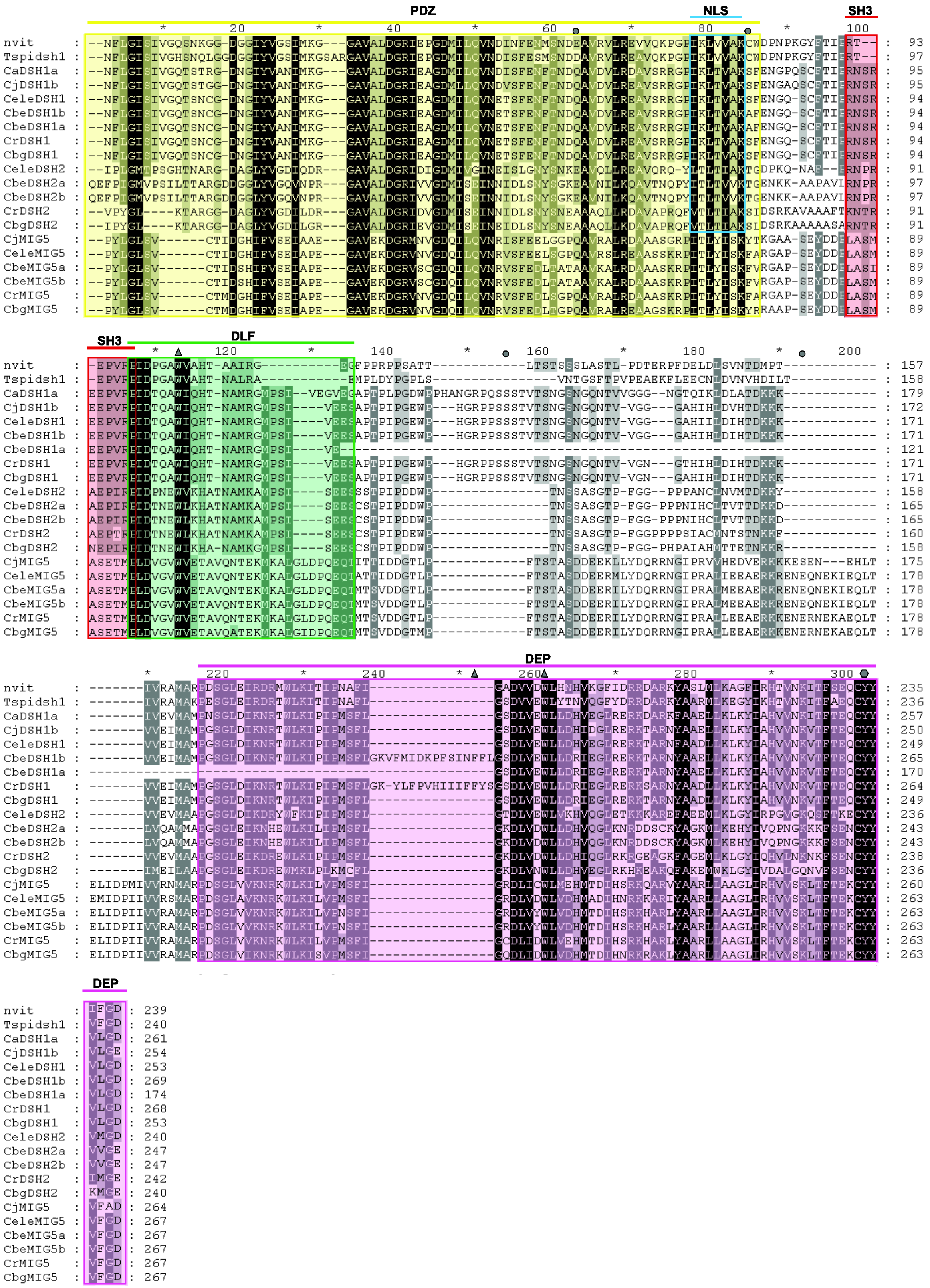

Figure S3 Protein alignment from the beginning of the PDZ domain through the end of the DEP domain from all caenorhabditids in this analysis, plus *N. vitripennis* and *T. spiralis* as outgoups. A nucleotide version of this alignment was used to generate the phylogenetic tree from figure S1. Domain features are highlighted in color and labeled, including PDZ, NLS, SH3, DLF, and DEP. The conserved tyrosine 473 (Y473) is labeled with a polygon (⬣). Codons identified to be under negative selection are labeled with a triangle (▲) while codons identified as experiencing diversifying selection are labeled with a circle (●).
