# Supplementary material for: Origin and Evolution of Dishevelled
Source: G3 (Bethesda). 2013 Feb 1;3(2):251–62. doi: 10.1534/g3.112.005314 (PMC3564985; doi:10.1534/g3.112.005314)
Supplement: Supporting Information [file supp_3.2.251_FileS1.pdf]

Supporting Information

All Dsh proteins identified and used in this analysis

The supporting information included all of the protein sequences used in this analysis and has the various protein domains, as identified using the SMART database (http://smart.embl-heidelberg.de/) highlighted, according the the key below.

Domain Highlight Key:
Green – DIX
Yellow – PDZ
Pink – DEP
Blue – Basic Region
Red lettering – SH3 binding domain in the proline-rich region
Dark Green – DEP-like fragment
Red – Dishevelled specific domain
Teal – Dishevelled C domain (mammalian specific)
Black & White – Signal sequence
Maroon – Coil domain
Grey – internal repeat

>Cele-DSH-1
MAESPPPVDSSLNAPNVGSPTTMMERLRLRDQTEENGKEDDFDNKSVSSAQYSQTSEATTAVK
QQPFLHTMTKVYCHIDDETDPYMLEVHVPPDLITLGDLKRVLMRTNFKYYRKALDPDSGYEVKAE
IRDDSQRLTPSPNNLFELFLLTIEGSTHSDGSSGKMRKYPSVPGPAPSNRNGPPMNYQHAAYQF
DNSMMSTDSESMISAAIPGYLKSAAYNRRFPQHYLGHRRHLEESTIGSESDARVFSDDDDRGST
TTDFTSVSRQHEKMAKKKKNKRNFRKPSRASSFSSITESSMSLDVITVNLNMDTVNFLGISIVGQT
SNCGDNGIYVANIMKGGAVALDGRIEAGDMILQVNETSFENFTNDQAVDVLREAVSRRGPIKLTV
AKSFENGQSCFTIPRNSREEPVRPIDTQAWIQHTNAMRGMPSIVEESAPTPIPGEWPHGRPPSSS
TVTSNGSNGQNTVVGGGAHIILDIHTDKKKVVEIMAMPGSGLDIKNRTWLKIPIPMSFLGSDLVEW
LLDHVEGLRERKTARNFAADLLKLKYIAHVVNKVTFTEQCYYVLGDECSDYARFRNEDGGPKYQ
WTIGMNGMSAGNGSSVMLPPPHLPGGMAGPPGAFKGMAPSMVSGYASMPASPFPPAQLQQQ
RREGSTTSGSSGGGIRKQRVVVLPRKPSSSANVPFDDSSTIYEESNNSFLMATGQRYEY
>Cele-DSH-2
MTDSPSPIDSSFDASDVATPCTVIAAKISLRNRNGLEEDQENLDSFDAFTETHETQESKNIAHGEH
EEDVSNIYVDDFSKEFGDTVSSVMEPLPKPLTFARTITKVYCHLDDQEHPYMVEVHVPPDCITLR
DVKRKLMRTNFKYYCIALDPDTGLEVKAEVRDDSRRLYPLKNGRFELYLLTVEGSVHSDTSSGRH
RRKQDGSSKGSSGSREYLRAAHHYDNPTPFSDDESQASSLPTYVKKAHAYNRKHAPQAYERHL
PHMKHNNRHNHHRQNHYEESTFDVTTESDDHYRDGVTYYDEDEDDSRSINTDLTSVSQVHLKQ
KWRQQQKEMRNKWKRMPSISTASSSFSSITESSMGLEVITVRLNLETIPLGMTPSGHTNARGDA
GLYVGDIQDRGAVALDGRIDIGDMIVGINEISLGNYSNKEAVQLLREAVQRQYLTLTIAKTGDPKQ
NAFPRNPRAEPIRPIDPNEWVKHATNAMKAMPSISEESSSTPIPDDWPTNSSASGTPFGGPPPAN
CLNVMTDKKYVVEVMAAPGSGLDIKDRYWFKIPIPMSFLGTDLVEWLVKHVQGLETKKKAREFA
EEMLKLGYIRPGVGKQSFTKECYYVMGDECADYTQLRGPDGGYKYPQSHASSASGHSSNNLIFP
PSMYPPQPPTAGAVQSSKFGHSTFFNDWRIRITTGVSISSRKGICQ
>Cele-MIG-5
MEPPCTSDCSQIKVFYYLDDETTPYVSVIEAREGVATLGNFKNSFTKRGYKYYAKELDPDIQREV
KVELTTDSDRLRKSQNGFYEIFLVSTPGYGTLPRNSGTMTRPQRTALDKRRRRSADFDATPYSD
ASLAPSTIVSRRAGEHLAELYTSNSEDPYQYDEHTRRTGDDSSLYEPLAARDMNKIYDDDRRRKK
QKKERFRRPYVPSTISSATESSVNSGLPRILEIYLPMKNVPYLGLSVCTIDGHIFVSEIAPEGAVEK
DGRVNVGDQILQVNRVSFEELSGPQAVRSLREAASSKRPITLYISKFARGAPSEYDDPLASMASE

TMPLDVGVWVETAVQNTEKMKALGLDPQEQTATTIDDGTLPFTSTASDDEERMLYDQRRNGIPR
ALIEEAERKRENEQNEKIEQLTEMIDPIIVVRSMARPDSGLAVKNRKWLKILVPMSFIGRDLVDWLV
DHMADIHNRKKARIYAARLLAAGLIRHVVSKLTFTEKCYYVFGDGILGNDRNSTDTTGTSGTTMRV
EATTEVTYVGSPAPHALAARVGRNIPHRLETTTLSPVAHDQTWLRRRRDCESPMTNDYASMVGE
SQIGMNPVGNYHVFGTKNNHRQVPAPSQVTSSSLTNGSGGLGGPPPTPLSSTMVLAASPIQSQN
AVNHDFDGENSSNSRTRILRT
>Bmal-DSH-1a
MDVSDTLDTSNTTDTIIEKTNKLKISSAVESKDESSKNSSCTSKAPLAKGTSCLEHGIGVPSQQTS
TKVYYHIDDEMVPYCTDVMVPPDKITLGDFKRVLTRSNFKYYCKAPAPDSGVFPEVKVEIRDDNE
CLHRSANGQFELFLLTSEGSSHSDGSSGLPLKSARLMFSKIIFRPIIVHCEKNFLVASTDSDSFISD
MRALPMQVKGISRRPFPQQYISQGHRGGRRFEDSTLGSESDARLFSDDDDRSRVSTSTDITSVS
RQHHAPAYRKRRNRRRFRQPSRASSFSSITESSMSLDVITVTLNMDTVNFLGISIVGQSSSRGDN
GIYVANIMKGGAVALDGRIEPGDMILQVNDISFENFTNDQAVDVLRESVARRGPIKLTVAKMWDS
GPRSAFTVPRHRDEPVRPIDTQAWIQHTNAMRGMPSILEGSEGAPTPIPGQYGRPASSSTATSN
GSVPNTIVGGAHFRLDAMTDKKKVVQMMVMPNSGLDIKNRTWLKIPIPMSFLGSDLVDWLMEHV
DGLRDRKDGRKFAGELLKEKLISHVVNKITFTEQCYYILGEECADYARLRQNPGGDDPGVRSEVG
SVLPPPPPGLVAAAAAQQSGRAWPQPTMIPQSAPSMVSGIENPANADGTTRFYLTNL
>Bmal-DSH-1b [SPLICE ISOFORM]
MLFSVPGPAPTMYPVGSMAYRQAVQQFDQSMASTDSDSFISDMRALPMQVKGISRRPFPQQYI
SQGHRGGRRFEDSTLGSESDARLFSDDDDRSRVSTSTDITSVSRQHHAPAYRKRRNRRRFRQP
SRASSFSSITESSMSLDVITVTLNMDTVNFLGISIVGQSSSRGDNGIYVANIMKGGAVALDGRIEPG
DMILQVNDISFENFTNDQAVDVLRESVARRGPIKLTVAKMWDSGPRSAFTVPRHRDEPVRPIDTQ
AWIQHTNAMRGMPSILEGSEGAPTPIPGQYGRPASSSTATSNGSVPNTIVGGAHFRLDAMTDKK
KVVQMMVMPNSGLDIKNRTWLKIPIPMSFLGSDLVDWLMEHVDGLRDRKDGRKFAGELLKEKLI
SHVVNKITFTEQCYYILGEECADYARLRQNPGGDDPGVRSEVGSVLPPPPPGLVAAAAAQQSGR
AWPQPTMIPQSAPSMVSGIENPANADGTTRFYLTNL
>Bmal-MIG-5
MREEAAAISTKVYYYLDDSTPYLSVVPVADDAITLGDFKKVFNKKGYKYFCKQLDEAVGCEVKVEI
RDDSTKLVKSANGLIELVLLSSSDNVHCSGTLPRVSNKTNKGQLTGAKLKDFNLRKRRSLHDLAS
ENRDLLRIHRNKSDENSITASSLSTVISKRAGEGLAELYASNSEDPYHLEDVNSRYFKHPGACXPV
FPASPLASPSGVLPCPRRQRRPRKERYRKAYVPSTISSVTESSMTSLSLPRIDVITLPMKNGVFLG
ISVLSHDGGIFVSDVHSGGIVDLDGRIEVGDQIVQVNRSSFENLSDVEAVDLLRKAAASRKPITLYV
AKRTCNNSDKRADILSGIASETMPIDISLWVESTKHNIVRPPKGLEEMVSVNDGDATLVAEEAETD
LEGAYAERRNGHIPSIQNCVKLKQLNPPDLNTSLNIEDIARRRENEENEQQLDNLNVDMDPVIILKY
MALPSSGLQIKNRKWLKIPVPMSFIGCDLVDWLMEHVHGITDRKAARIYASKLLAEGHIRHVVNKL
TFTEKCYYIFEDSILSVRNKNKSDSSLGKAGAEVTTEVTYVGSPAPAHLSRTSARNTLGGKAIFDQ
SWPHLTITSSEQRKSFCGSSTNDYASVMGPDMIDSTLLTEAPTLKLSHRTLPNRLDMEQRINGCE
VAQPPNTPNSLLHEQRNADSETEFETVEDNKFLVIQK
>Bxyl-DSH-1
MSSMTETSMSLQVMHVRLNMDTVKFLGITVVGQSSARGDNGIYVAHVMPGGAVALDGRIEIGDM
ILEVNDVSLEKMTNDEAVEFLREAVTTKGPIKLTVAKCVDSNRANFLVSSREPVRPIDTRAWVQHT
NAMIGMPMNSIPESAEEAPTPIPGQYPQNNSHPVFCRPQSSSTATSNGSGGPKNTVVGIPGAYF
ALPPRLDLSTDKKIVAKAMAMPNSGLEVRNRTWLKIPVPMSFLGSALLDWIHEHVEGIRDRKEAR
KYASELLKDRLIAHVVNKSSFTEQCYYVFGEDCQEILKLRNEDGTPRTDLMPQPPMHPPKPIPGH
SAFGWTQARSTGDYASMPVSPYPGNGPFIPTNSLNQPLNKHGDIHSQASGNSNDGSSSSDQRR
KPLLPAAPPLPMGLNPLYQQGPQPPPSQPPPLPPFEARDPSSLKLDDLGSNQQLQALVSQGFTV
DHL
>Bxyl-MIG-5
MASTKVYSYLDDDNTPFLTSVPVSASQITLGDFKKALPKRNLTYSQKVFDENVKKHVKRTIHDDS
EPLQLNENGVVELFLTSNGPIGTLKTNGRHYKNLPPHLANYGVGDLDVCGGQRVSMVTAEPSIS
GAGSYLSKRAGEQLASIDNTSASEDPYVFEQSSIANQSSDYLRVMNTTDYSRRRKKRERYRRPY
VPSTISSASGMSSVPQVTEIMLDLREHALGIDIGMCDGAVLITSIAKNSAADRCGALGVGDQIVQV
DNTSFEELSDEQVVSLLRKISSQKRVVRIVVARYKRDSDQRSDALSALCETAEIDVSLWVEGTKQ

ANPDAPFDDFHQNPNTSAHINEAAEETSDEERAAYDDRRNGIGAHFVPQIKLMRAIHQRTEANGH
IPNDENDRHSRLSATDAMEIVVRQMARLDSGLKIKDRKWLKIPIPMSFIGHELVNWLLENVDGLDN
RKQARNYAKQLLEKGFIKHAVNMNSFSEKCYYKFQEKICEERLLMEQQAKLNTVDSPTEITYMSG
PSSPHQPHRQIPNNYHTGPLDVLQQRQINYSGTQPPPPRINPQMTSKSQPALPNTLPMNSTQVQ
RPMNSTAVDLSKWEFSPIPARNYRDCESTSGIYDVVKQ
>Tspi-DSH
MEIRKDSSRTSVIGKGNYNGENDKKVVKEEISDDAVKLPTFNGCVESWLVTSEGSTHSDANGPT
TQHGHSSPRHPPTRSSGLGESRPPSFHGGRADSRDNLVPSGCSSETESTLSGLPKFPRYGTKLD
RRSFHPQFHERVRYPKGMRARCFDVGHGLTSDLDSTSFMDSEDDQASRISSSTDITSVSRQYLK
RKKRRRLPRTLLSRASSVSSITESSMSLNIITVTLNMDTVNFLGISIVGHSNQLGGDGGIYVGSIMK
GSARGAVALDGRIEPGDMILQVNDISFESMSNDDAVRVLREAVQKPGPIKLVVAKCWDPNPKGY
FTIPRTEPVRPIDPGAWVAHTNALRAEMPLDYPGPLSVNTGSFTPVPEAEKFLEECNLDVNVHDIL
TIVRAMAKPESGLEIRDRTWLKITIPNAFLGSDVVEWLYTNVQGFYDRRDARKYAARMLKEGYIK
HTVNKITFAEQCYYVFGDICDNFAGLRLEPHREEPPQLEHDSISALPPPPMTPSTLWSSNVGHPY
YPHQACPSFVQTSSVASGYVPIPYQYNNEAGSFPSLVVGGAPPTSRSDANSAGSAGSNGSNES
ERVRKGVVGGLLPMTSTPGVPLDPNIQLKGYGPPVGAMTSKGMIGPLSPTSRNVNDLQSDISGS
RQSFRMAMGNPCEFFVDVM
>Asuu-DSH-1
MGEGIGMSLLLDCPISQAACYLNFVKHPNVSMYMKLFVEVTSSLQMPTDDLVDVDENRRVVKLS
QSFELKGRKSGRSRSLPHTDKKGCRCHRAGRRFEDSTIGSESDARLFSDDDDRSRVSTSTDITS
VSRQHQANAYRKRRNRRKFRQPSRASSFSSITESSMSLDVITVTLNMDTVNFLGISIVGQSSSRG
DNGIYVANIMKGGAVALDGRIEPGDMILQVNDISFENFTNDQAVDVLRESVARRGPIKLTVAKMW
DGGPRSAFTVPRHRDEPVRPIDTQAWIQHTNAMRGMPSILEGSEGAPTPVPGQYGRPPSSSTM
TSNGSAPNTVVGGTHIRLDTTTDKKKIVHMMVLPNSGLDIKNRTWLKIPIPMSFLGSDLVDWLME
HVDGLRDRKDGRKFAGELLKEKLISHVVNKITFTEQCYYILGDECAEFARLRQNPSAGDEQGVRS
DVGSVLPPPPPGLMAAAAAQQTATRAWPQTTLLQNAPPSMVSGYASMPISPYPGQPPTQPFAP
SIMHGVGNPVSIYKGGCAPDVHSQTSSNDGSSGSEHIRASQLAHSIIEKGTACPHLRLRDTDVVIY
QDNVVTQSHGTRARSSLLEIRFGKSEQAAHVRIKCGLYFPTCCGLCFPFYRQKYAGAAFTRERK
CEFL
>Asuu-MIG-5
MPLDENAAVASTKVYYYLDDNTPYLSVIPVPENKVTLGDFKKIFTRKGYKYFCKQLDKAIGCEVKV
EIRDDSSRLEKSANGLIELVLLSTVTPSGTLPRAITNKTEHNGVGNRDPVVNFESDLKVRKRRSLH
ELADNVVVNADPLANGCRASMRNSNEDSITASSLSTVISSYSILERAGEGLAELYTSNSEDPYRFD
GSNSRFTLNSEACSSAYGGIRMAASSAASCLKAHRQRRPRKERYRKAYMPSTISSITESSMASLS
LPRIEIVKLLMTNGAFLGISVLSNDGGIFVSDIIKGGAVALDGRIEVGDQIVQVNKNSFENLTDAQAV
QLLRQAAVSRRPITLYVVKRPCNTDSRSDVLSGLASETLPIDISLWIESAKQNSVKPLKPFAIDETN
SIMVENTLGEEEHETDMEGAYAERHDMIQIPPTSNKGRTTQVVTTKRGVMTTEDVARRRENEEN
EQLVDNLNVNMDPRIILKFMARPDSGLQIKNRKWLKIPVPMSFIGRELVDWLLDHVHGLHDRKAA
RSFASKLLADGHIRHVVNKLTFTEKCYYVFDDSILSVRSINHSDGSNGKTGAEATTEVTYVGSPAP
QAAARLAVRNCNDSGMPPPPINNKMPAEIDQTWPVSPITIYGPTQRRKDCDSPVTNDYASVIGPD
VVTSTMLGTSLTEAPTLKLPRHGFIGRGNEMRRRMDDEIVVAQPPNTPSSLSAAPNIGVDLGADF
DSLEQDRRNMLKENR
>Ppac-DSH-1
MSGYQVDTDDVSSTSGSGGGYYGYGGSTSGGSVYYGMSGATVRNRGTMTIREADEDGGERDD
GEGSMSTDLTSVSRQHEKMLARRRKAQRTYHRRPSRASSFSSITESSMSLHVETVTLNMDTVNF
LGISIVGQSSARGDNGIYVANVMKGGAVALDGRIEPGDMILQVNEVAFENFTNDQAVDVLRDAVE
RKGTIRLTVAKSIDSNQRSDEPVRPIDTGAWIQHTNAMRGMPSILEGSEGAPTPLPGERLHHHQQ
QMQQRPATSSSATSTGSNGQNTVVGSAGLPGTMPPLPRLDVHTDKRRVVEVMTRPGSGLDIKN
RTWLKISIPMSFLGSDLVEWLLEHVSGLRERKEARKYATELLRQRLIAHVVNKISFTEQCYYVLGE
QCADFARYRTVQVEEGGVNGGGGTQPTWQWGGGGPRPTGITPGGVGGSMSTLPAESHLFFLV
KCNFINCASQVSGYASMPMSPHPPLFGGASAPGVRTFGGEGSQISSTDGSGSSDAHKRGSI
>Ppac-MIG-5

MQVHDIASILKKKEMGVESELMTNQLPLRIYESEGLVFEWMWLRSVPVMGLDSNTYGKVFEDFD
LHELKEMLTVNYTSWRKYVWMVLDGMHVLSFPLPIPPQELIIYRLEDMKMKYTSIQSESPNNLLR
YFELRSFDFRPADEELKGFIESELRNGSMAIDGVDWREWLCESDVLNGMSDTVAEDRQLCWIED
VNGKEKKRLMAATEAMRVDTPYLWKDVMELAMEKLQLMALPTMKFNVMKYGETKRQRILMKEE
KSGEIPPRYFKEIHCMSRENIVPPGVNETTKVFYYLNDEPTPYVEIIHVGAESVTLGHFKREVKVEL
IGDETKLTRNGENGLFELFLLSTGTQNGGGGGTLQRKTNGTLTRRVGKERNDRDIYNTHRVHSD
DYDSSSRQDTLMSRRAGEILAESITSASENLYEYDDSRQYNAVMGDEASRIDGPRRRKARKNRV
RKSYVPSTVGSQPESRYGGIPGTSLSLPRILEVNLQIGPNDLLGISVVSVEGSILISDVFPVGVVAR
DGRIDVGDQIVQVNTRSFENLSDQQAIMILRKVAAAKKPLTLYVAKRTMSTAESDPLCTLASETLP
LDISLWVENAVHCTERQRFGVDGSVDGTILSEGVGRAASICTEDEEEERMLYVQRRNGMGIRER
GLEQPPIHLHSAPPPRGNYSEGSGYTERLSTRINPHSLINIISQPNSGLTVKNRKWLKIPVPQSFIG
VELVDWLVQNVEDLGERKEARKYATHLLEKGLIKHVVNKRDFTEKCYYVFNGE
>Scar-DSH-1
MYFSLAIWPTVATRGFVLVLVMFDLLSFHRGLCNVRVSSPVLLPLIVKPNMSDSAVSNSTLEPIVA
VTARMTIKDDSALVGPSSDVSDRQSDVSGSQSLQPIGGPESDQCVANANVPSSKQTTKVYYNID
DETTPYCTEVPVPPDRITLRDFKNAFNRQQNFKYYCKRIDKELGKEVKSELRDDSQPIERSQNDIF
ELFLLTAEGSTHSDGSSGAHTRPSLRGTHMVPAPAPSGAHFDAYNAQYMHGRNFDPSCKITSLQ
EKTDLYHFSGEHRLRIDVRKRTPVLPAGQHEPKAAVLPASPSADSSCLRSFQSNEGYGKRPPCR
LSLFANTLLLLNPNLAGLGCCSSPSSFYDFQSWLPLLIYCAAFDNLSFIPRSIIPHRKKIHMSGADR
NSAERIDRLRERAKRLQELITDRRRSCRYVSEKCERFDVSAEPETSLKANKPTTAPEPMVKMREP
KPYVKSRTRELVESLINILPREPRDVRRSESLAVIVPSSTSTTAPMVIRHSSVSASNSKRQKDSDA
PPGKQEQYAVIKKKSKRLSLVEKLPFLSAYSERQRPKSTSVAFERDTESLLPNRSEPPEAESESK
APPKAESESKAPPNAEELVEPAEKIHRRSLPNPVRYRPKSIYQQSQMLPSGSTPKTLTRAMTMVD
SPRRLPPLIEVTAPEPSEAGESAAQSQSEAAAEKTSRKPRRYTGSHTTSTYQGGRDAAPDASPR
RHLIGFLHRTSHLSLTSELSADASFYLIGHRGRGRFEESTIASESDARIFSDDDDQTRVSTSTDITS
VSRQQHHNIYRKRKNRRKYRQPSRASSFTSITESSMSLDVITVTLNMDTVNFLGISIVGQSSNRGD
NGIYVANIMKGGAVALDGRIEAGDMILQVNDVSFENFTNDQAVDVLRDAVARRGPIKLTVAKCWE
SAQKNCFPVPRPRDEPVRPIDTQAWIQHTNAMRGMPSIMEGSEGAPTPVPGQYSQHQRPHSSS
TVTSGGSGPNTVIGNGPNHIQLDSKMDKRKVVEAMAMPNSGLDIKDRTWLKILIPMCFLGTDLVN
WLIEHVHGLKDSKEAKKYAMELLQAKLIKHVVKKVTFTEQCYYELGEEFARHRLMTGECSSGRS
DVGSLPPPPPGFLQSQPQQAARWQVMPGSNGVPSAPSMVGNHAPSSHMVPPRHGGVPSSSE
YGIVSGGHPCARVTIENGTTSHFILDI
>Scar-MIG-5
MEVKVFYYLNEGDVPYVSVLNPASGAAPTLGDFKKVFNRAGFKYYCKELDTDIGREVKVELTDD
KAPLLRAGSGLIELFLVQQQQQNFNSGTLPRTGKQNHEAAAGAAFEPGGRLKKRRSLYGLSSVD
GFDIGGGQRVSMVTNEQSLAESSQGTVLSRRAGEHLADMYATNSEDPYNMEDPSASSFSAASS
AYGGIPIGNNGRLPQNRHRKPRKERYRKAYVPSTISSAAESSLTSQSLPRIDVIKVSMKNAITLGIK
VVGHDGGIFVSLILPDGAASQDGRLEVGDQIVQINEESFENLNDQQAVSILKKASKSKRSVTLYVS
KRPRAHDDGSSDVLTGMTANETMPLNISSWVKSTMHRKVEKHVPFQSVVGESTLDPSESMTIAD
ETSDEEQAAYLDRRGGVGPRFVPALGMRNQIAEDVFMRQKENDENDVMIDTLSVNMDPRIILKV
MAKPDSGLQIKNRKWLKILVPMSFIGSGLVDWLLMHVQGLHDRKSAREYASQLLQEGLIRHVVNK
MTFTEKCYYVFDESIMQMANRGSRGGAHSSGGEVTTEVTYVGSPAPGTDKTQPRPLDSNLTVQ
NANVNATWPISPITLYGNQTARRCESPAVTNDYASMIGTEFVQQQHQMPVMMPSEAPTLKIGTQ
SPSTRSPFFPPPPPSLQRSMSPPPNTPNTLLSSVLLSGATGSSRLTHQQSSHM
>Pred-DSH-1a [SPLICE ISOFORM]
ARKKRAPRRYIHSHTTSTYDAAANEKVPDASERRKLIGFLHRTSHLSPLVPEGIDTVTTVASVTNV
VGNRRRYLEDSTVGTESDARVFSDDESRVSTSTDNITSVSRQHNNYRVRQRRMRRHLRQPSRA
SSMSSMTETSLALEVMTVTLNMDSVNFLGISIVGQSSTGGDNGIYVANIMKGGAVALDGRIQPGD
MILQVNDTSFENFSNDQAVEVLKEAVVHRGPIKLTVAKSDTGRFDAFNVPSEPVRPIDMRAWIQH
TNAANNLPHIPEGSEGAPTPIPGQYPHNYGRAPSSSTATSNGSNGHTILGPNGQVFILPKKLDLST
DRKRVVSVMALPNSGLDIKDRTWLKIPIPMSFLGSDLVEWLIDHVEGLCDRKEAKKYATDLLKEG
YITHMVNGNKFSEQSYYMIGRECSDAVRLRLSEEGASRSDVASSLPAPPPHLAPQPWTANSAMT

SDYVPISPFPQQHQMFYRGHDMFSQVSGQSNGDSSGSEARRARPILPPAPSSVTNVPYNAGAN
FFRQQTARLGDFPKNLAASRQSFRIACGNQTSEEFFIDHL
>Pred-DSH-1b
TFCVAVFIGQPMLSHLMSESRDTSATTAHSSSVSVDDAVTAIDKLALTSNGCNGALPGPSSSDAD
AKSDAASSEARSVPRTNGSGSTVSANVTKIVYHVDDESMPYVTEVPVAPDRVTLLDFKKMLNKL
NYKYYCKSNDPEVGGEVKAEIRDDNQQLFRSLNGQFELFLLTTDGSNNSDGGASSGFSRNVTQS
VPGPAPPSAYPPFGLPQHMRQYSGYDNGNRRRYLEDSTVGTESDARVFSDDESRVSTSTDNITS
VSRQHNNYRVRQRRMRRHLRQPSRASSMSSMTETSLALEVMTVTLNMDSVNFLGISIVGQSSTG
GDNGIYVANIMKGGAVALDGRIQPGDMILQVNDTSFENFSNDQAVEVLKEAVVHRGPIKLTVAKS
DTGRFDAFNVPSEPVRPIDMRAWIQHTNAANNLPHIPEGSEGAPTPIPGQYPHNYGRAPSSSTAT
SNGSNGHTILGPNGQVFILPKKLDLSTDRKRVVSVMALPNSGLDIKDRTWLKIPIPMSFLGSDLVE
WLIDHVEGLCDRKEAKKYATDLLKEGYITHMVNGNKFSEQSYYMIGRECSDAVRLRLSEEGASR
SDVASSLPAPPPHLAPQPWTANSAMVGHPHHPPQMPQQGGRLLVATPNSTRKSPVLNGVPNG
GGNGAYPTSYAADNSSYGVIGGVNGVAAPPPTWRLTEESLGSLRHMADV
>Pred-MIG-5
MSDAGSSSFTTVYYYLDGATPFKSEVPVPPEKITLGDFKRVFDRQNYKYFCKEFDPMLKKDVKV
ELTKDSQKLNKSESGVIQLVLLPVYPASARASDSSGGTLPRASRSDTGRNPPRRGFAQPKRALSI
TDETDYANNRYSLATDPASTAYSRRAGEQMAETLSSSDFDDDNEREDTSDEEANRESGSSTSC
RRPHGNRRNRQPRAYVPSSRGSKSATSQSDSLPGIAEICIQIYPHQNLGFNVADHDGGIFISDIFD
DTAAGNCPDLSVGDQILEVNSVCFEHLTFEQALAQIKKATKTAKSEATETKPGKIKMHVARLRVS
DQHSESGLSAGLGDTIPFEVSEWVMATTAENVDRFDDPLNNSRFDDGGVTSDEERAAYIDRRNG
VGARLVPALHNFRSNNGFDVPSSPHNRNHPALLMPPPPPRSRENDENEFLNAPLSVDTDPITILK
RMVHPASGLEITTRKWLKIPFPDSFIGNEMLAWLMEHVEGLKNKKAARKYAASLLTKGLIRHVLD
GKQFDKKRYYFFADNIISYRRQIEHARATASASRLPTSATAATEVTFLGSPSYPPPAGPTANGGLL
KSIPASCGALPSSSNNFFSSQNTYLTGSRAPTRSMHSQQRLPQATRSPYYPAPIGPAMYMPQPQ
ASPGLPQWPISPLIGSNERRPSCTSPVTTNEYASMINAEVNSNYMSVPTLPRGYGNARLMNNGT
PPPNTPNTALQGTASRSQVPIQHR
>Minc-DSH-1
METTKLDNDESNEVRMLEKETVQLDINKAMNTNNDLNSQAEKIDNVSDASSHLTHSSMSICSSAA
GRGATKVYYHIDDEQTPYCTELPVPNNRVTLGDFKRVLNRTNFKYYCKAIDSEVGGEVKAEIRDD
AQQLTRSSNGHFELFLLTAENSNSDGNSSGVSKLASMKKVPLGPTTIYPFTSSQHYRPRNRCDS
NYVSGNHTRQPFDDSTLYTNETDHRLYSDDESRLSTSTDNITSVSRQRYNLYRRRRRQAPRNRR
KPSRASSLSSMTETSMALEVITVTLNMDSAVNFLGISIVGQSSSRGDNGIYVANVIKGGAVALDGRI
EPGDMILQVNDVSFENFKNDKAVEVLKQAVNRQGPIKLTVAKSFDSGRANYFSVPVREPVRPIGS
EGAPTPIPAHNQYHPREQMVNMLNIPPSITQHQQRSACSSNTTATSTNGSGGVAPQIVLGQGGV
FLAVQPRLDINSDKRIIIRAMVAIGSGLEIRDRTWLKIPIPMSFLGSSLVDWLIQNVDGLKTRKEARK
YASDLLKERFIAHVVNKQVFTEQCYYVFGDNCSDLLLLRNGIEHGSALVRNPQNQAVLANQRLFH
DRDCGTQVPYMLHTNFGGSEYAMPSVSPYPPPVIPMQTTLEYSNQQRQLHKLVPQNPVHTQTR
FDQFGANSQASNNSNEESSGSEHRRKAMLPPAPSLTSIGQPQYFQPHQFFPGQIHRTSQPTGPP
PTTPDDVNSMMLNRFCGINALLAMDVYANYMIQQNGIRQEQQPTLSQMKQEFAPYSGSSSQAT
GTGKFVAMPLLQQTGKVFVGPGQHREAQLIGLGLRKLRTFEREDIERAKRYALDQSVKFVMLKQ
REAHQQQVNFIWDFLCFKLLFSRIRRMTILFCRGHMYSID
>Minc-MIG-5a
MSEQPKGSTKVYYHLDDSTPYMSELMTSPDRLTLGDFKRAFNRKGYSYFCKEWDPNLKREVKV
EIVNDRQLLRKSVNGLFELFLLSQQNSTQPFSNTTSNMGADQESDLQTLISKRAGENLAEMYNST
SEDPYNRTSVSSSSIFQRRPLPGAPMPSAESTAESAAERTLLVNTSAARQKRLRKQRYVPSTISS
ESESTYSLPRIEEVKLRLQDAPLGISVASQCGSIFIYHIQHGSAAERCCRLEVGDQIVQIDETRFED
LNEKQALEVLKKLNSVKKTITMYVAKRARTNGGESSEDHKSDPLSLLCETQQLDISQWVESTTNK
NCVEQVRPFAEIPPVAKVERGTLPIDGCKVKNQTVDETSDEEKAAYLDRRNGVGARLVPIIHQVR
FHQQQQQQKSEEMHKMQMINLENNPTTSSCPVMLGTLEESSPHLDCHPPLIQLPLHAAMDPKI
ILFRMVEFDSGLEIRNRKWLKIPVPMSFIGDEMIDWLISNVQGFRDRRHARSFASNLLAQGLIKHD
EIIADRLRIEEQQKQQHLLKQHKPDADFQTTSIKKPPALAPESNTEITYMSNPPVTSTCLGSSGAP
QNVSSHTHPTLTDQQQRTQYVIANNPRYAPGPMSNNFCGQKINKNTAALITDAANLNKIDKNAPK

NAVATNIPLRTLNPPKFGLKLPVWPISPILSFYRPQNQEVSQQQRGRCDSPEASKTNDDYASMIQ
GEINCGAQFDDGLNTFQNQQQ
>Minc-MIG-5b
MSELSITPDRLTLGDFKRAFNRKGYSYFCKEWDPNLKREVKVEIVNDRQLLRKSRGQAKIWQKC
TIQHRKILTTELQSSSSIFQRRPLPGASMPSAESTAESAAERTLLVNTSAARQKRLRKQRYVPSTIS
SESESTYSLPRIEEVKLRLQDAPLGISVASQCGSIFIYHIQHGSAAERCCRLEVGDQIVQIDETRFE
DLNEKQALEVLKKLNSVKKTITMYVAKRARTNGGESSEDHKSDPLSLLCETQQLDISQWVESTTN
KNCVEQVRPFAEIPPVAKVERGTLPVDGSKVKNQTVEETSDEEKAAYLDRRNGVGARLVPIIHQV
RFHQQQQQQKSEEMHKMQMINLENNPTVSSCPVMLGTLEESSPHLDCHPPLIQLPLHAAMDPKI
ILSRMVEFDSGLEIRNRKWLKIPVPMSFIGDEMIDWLISNVQGFRDRRHARSFASNLLAQGWIKHV
VNISSFNEKCYYVFNDEIIADRLRVEEQQKQQHLLDQKPDADFQTASIKKPPAPAPESNTEITYMS
NPPVTSTCLGSSGAPQNVSSHLHPTLTDQQQRTQYVIANNPRYAPGPMSNNFCGQRINKNTAAL
ITDAAHLNKIDKNAPKNAAATNIPLRTLNPPKFGLKLPVWPISPILSFYRPQNREVSQQQQRGRCD
SPEASKTNDDYASMIQGEINCGAQFDDGLTFQNQHQQQHLLKSKVTSPLGTGKYSKDLKNDVN
MSNKLSQSLATTNTTKPPPLPKHQFPSEFLTLQ
>Minc-MIG-5c
MSEQPKASIKVYYHLDDSTPYMSELPIPPDRLTLGDFKHAFNRKGYSYFCKEWDPNLKREVKVEI
VNDRQLLRKSVNGLIELFLLSQQNSTQPFSNTTTNLGADQESDLQTLISKGRRKFGRNVQFNIRG
SLQQNFNPPLAFFKEGLYLVLLCLQLKAQQRTLLVNTSAARQKRLRKQRYVPSTISSESESTYSLP
RIEEVKLRLQDAPLGISVASQCGSIFIYHIQHGSAAERCCRLEVGDQIVQIDETRFEDLNEKQALEV
LKKLNSVKKTITMYVAKRARTNGGESSEDHKSDPLSLLCETQQLDISQWVESTTNKNCVEQVRPF
AEIPPVAKVERGTLPVDGSKVKNQTVEETSDEEKAAYLDRRNGVGARLVPIIHQVRFHQQQQQQ
KSEEMNKMQMINLENNPTTSSCPVMLGTLEESSPHLDCHPPLIQLPLHAAMDPKIILSRMVEFDS
GLEIRNRKWLKIPVPMSFIGDEMIDWLISNVQGFRDRRHARSFASNLLAQGLIKHVVNISSFNEKC
YYVFNDEIIADRLRIEEEQKQQHLLKQHKPDADFQTASIKKPPAPAPESNTEITYMSNPPVTSTCL
GSSGAPQNVTSSHPHPTLTDQQQRTQYVIANNPRYAPGPMSNNFCGQKINKNTAALITDAANLN
KIDKNAPKNAAATNIPLRTLNPPKFGLKLPVWPISPILSFYRPQNQEVSQQQQRGRCDSPDASKT
NDDYASMIQGEINCGAQFDDGLTFQNQHQQQHLLKSKVVS
>Mhap-DSH-1
MEATKLEFNEMQVLEKETVQLNINEANDTSKKDLNSKAENVDRTSDVSSHLTDSSMSVCSSAAA
RGATKVYYHIDDEQTPYCTELPVPNDIVTLGFLFANQGHQFEIIGDFKRVLNRTNFKYYCKAIDSE
VGGEVKAEMRDDAQQLIRSSNGHFELFLLTAESSNSDGNSSGVSKLASMKKRQTFEDSTIYTTET
DHRLYSDDESRLSTSTDITSVSRQRYNLYRRRRRQEPRNRRQPSRASSLSSMTETSMALEVITVT
LNMDSTVNFLGISIVGQSSSRGDNGIYVANVIKGGAVALDGRIEPGDMILQVNDVSFENFKNDKAV
EVLKQAVSRRGPIKLTVAKSFDSGRANYFSVPAREPVRPIGSEGAPTPIPAQYPQGHMINILNAPP
SISQRQQRSACSSNTTATSTNGSGGVAPQTVIGPGVFLTLQSQLDLNTDKQIIMRVMAEINSGLEI
RDRTWLKIPIPMSFLGSSLVDWLLQNVDGLKSRKEARKYASDLLKERFIAHVVNKQASYL
>Mhap-MIG-1
MSEVQIPLDRLTLGDFKRVFNRKGYSYFCKEWDPNLKRSSSSIFRRGPLPGAPMPSAESMTESA
AERTLLVNTSTARQKRLRKQRYVPSTISSESESTYSLPRIEEVKLRLQDAPLGISVASQCGSIFIYHI
QNGSAAERCCRLEVGDQIVQIDETRFEDLNEKQALEVLKKLSYVKNKNCVEPFAEIPPVESRIGIN
QITGALPIDEFKVKNQTIDETSDEEKAAYLDRRNGVGARLVPIIHQVRFHQQQKADVKQRIKMVDL
ENNPTTSSFPITLGIVEECSPHPDCHPPLIQLPLHAAMDPIVILRRMVELDSGLEIRNRKWLKIPVP
MSFIGDEMIDWLIANVQGFRDRRHARSFASNLLAQGLIKHDEIIASRLHFEEEQKQLLLKHQQNID
CQNTSIKKAPAPPPESNTEIT
>Dmel-DSH
MDADRGGGQETKVIYHIDDETTPYLVKIPIPSAQVTLRDFKLVLNKQNNNYKYFFKSMDADFGVV
KEEIADDSTILPCFNGRVVSWLVSADGTNQSDNCSELPTSECELGMGLTNRKLQQQQQHQQQ
QQQQQQQHQQQQQQQQQQVQPVQLAQQQQQQVLHHQKMMGNPLLQPPPLTYQSASVLSSD
LDSTSLFGTESELTLDRDMTDYSSVQRLQVRKKPQRRKKRAPSMSRTSSYSSITDSTMSLNIITVS
INMEAVNFLGISIVGQSNRGGDGGIYVGSIMKGGAVALDGRIEPGDMILQVNDVNFENMTNDEAV
RVLREVVQKPGPIKLVVAKCWDPNPKGYFTIPRTEPVRPIDPGAWVAHTQALTSHDSIIADIAEPIK
ERLDQNNLEEIVKAMTKPDSGLEIRDRMWLKITIPNAFIGADAVNWVLENVEDVQDRREARRIVSA

MLRSNYIKHTVNKLTFSEQCYYVVNEERNPNLLGRGHLHPHQLPHGHGGHALSHADTESITSDIG
PLPNPPIYMPYSATYNPSHGYQPIQYGIAERHISSGSSSSDVLTSKDISASQSDITSVIHQANQLTIA
AHGSNKSSGSSNRGGGGGGGGGGNNTNDQDVSVFNYVL
>Hsap-Dvl1
MAETKIIYHMDEEETPYLVKLPVAPERVTLADFKNVLSNRPVHAYKFFFKSMDQDFGVVKEEIFD
DNAKLPCFNGRVVSWLVLAEGAHSDAGSQGTDSHTDLPPPLERTGGIGDSRPPSFHPNVASSR
DGMDNETGTESMVSHRRERARRRNREEAARTNGHPRGDRRRDVGLPPDSASTALSSELESSSF
VDSDEDGSTSRLSSSTEQSTSSRLIRKHKRRRRKQRLRQADRASSFSSITDSTMSLNIVTVTLNM
ERHHFLGISIVGQSNDRGDGGIYIGSIMKGGAVAADGRIEPGDMLLQVNDVNFENMSNDDAVRVL
REIVSQTGPISLTVAKCWDPTPRSYFTVPRADPVRPIDPAAWLSHTAALTGALPRYELEEAPLTVK
SDMSAVVRVMQLPDSGLEIRDRMWLKITIANAVIGADVVDWLYTHVEGFKERREARKYASSLLKH
GFLRHTVNKITFSEQCYYVFGDLCSNLATLNLNSGSSGTSDQDTLAPLPHPAAPWPLGQGYPYQ
YPGPPPCFPPAYQDPGFSYGSGSTGSQQSEGSKSSGSTRSSRRAPGREKERRAAGAGGSGSE
SDHTAPSGVGSSWRERPAGQLSRGSSPRSQASATAPGLPPPHPTTKAYTVVGGPPGGPPVREL
AAVPPELTGSRQSFQKAMGNPCEFFVDIM
>Hsap-Dvl2(>hsap_g0044131)
MAGSSTGGGGVGETKVIYHLDEEETPYLVKIPVPAERITLGDFKSVLQRPAGAKYFFKSMDQDFG
VVKEEISDDNARLPCFNGRVVSWLVSSDNPQPEMAPPVHEPRAELAPPAPPLPPLPPERTSGIG
DSRPPSFHPNVSSSHENLEPETETESVVSLRRERPRRRDSSEHGAGGHRTGGPSRLERHLAGY
ESSSTLMTSELESTSLGDSDEEDTMSRFSSSTEQSSASRLLKRHRRRRKQRPPRLERTSSFSSV
TDSTMSLNIITVTLNMEKYNFLGISIVGQSNERGDGGIYIGSIMKGGAVAADGRIEPGDMLLQVND
MNFENMSNDDAVRVLRDIVHKPGPIVLTVAKCWDPSPQAYFTLPRNEPIQPIDPAAWVSHSAALT
GTFPAYPGSSSMSTITSGSSLPDGCEGRGLSVHTDMASVTKAMAAPESGLEVRDRMWLKITIPN
AFLGSDVVDWLYHHVEGFPERREARKYASGLLKAGLIRHTVNKITFSEQCYYVFGDLSGGCESYL
VNLSLNDNDGSSGASDQDTLAPLPGATPWPLLPTFSYQYPAPHPYSPQPPPYHELSSYTYGGGS
ASSQHSEGSRSSGSTRSDGGAGRTGRPEERAPESKSGSGSESEPSSRGGSLRRGGEASGTSD
GGPPPSRGSTGGAPNLRAHPGLHPYGPPPGMALPYNPMMVVMMPPPPPPVPPAVQPPGAPPV
RDLGSVPPELTASRQSFHMAMGNPSEFFVDVM
>Hsap-Dvl3
MGETKIIYHLDGQETPYLVKLPLPAERVTLADFKGVLQRPSYKFFFKSMDDDFGVVKEEISDDNA
KLPCFNGRVVSWLVSAEGSHPDPAPFCADNPSELPPPMERTGGIGDSRPPSFHPHAGGGSQEN
LDNDTETDSLVSAQRERPRRRDGPEHATRLNGTAKGERRREPGGYDSSSTLMSSELETTSFFDS
DEDDSTSRFSSSTEQSSASRLMRRHKRRRRKQKVSRIERSSSFSSITDSTMSLNIITVTLNMEKYN
FLGISIVGQSNERGDGGIYIGSIMKGGAVAADGRIEPGDMLLQVNEINFENMSNDDAVRVLREIVH
KPGPITLTVAKCWDPSPRGCFTLPRSEPIRPIDPAAWVSHTAAMTGTFPAYGMSPSLSTITSTSSS
ITSSIPDTERLDDFHLSIHSDMAAIVKAMASPESGLEVRDRMWLKITIPNAFIGSDVVDWLYHNVEG
FTDRREARKYASNLLKAGFIRHTVNKITFSEQCYYIFGDLCGNMANLSLHDHDGSSGASDQDTLA
PLPHPGAAPWPMAFPYQYPPPPHPYNPHPGFPELGYSYGGGSASSQHSEGSRSSGSNRSGSD
RRKEKDPKAGDSKSGGSGSESDHTTRSSLRGPRERAPSERSGPAASEHSHRSHHSLASSLRSH
HTHPSYGPPGVPPLYGPPMLMMPPPPAAMGPPGAPPGRDLASVPPELTASRQSFRMAMGNPS
EFFVDVM
>Mmus-Dvl3
MGETKIIYHLDGQETPYLVKLPLPAERVTLADFKGVLQRPSYKFFFKSMDDDFGVVKEEISDDNA
KLPCFNGRVVSWLVSAEGSHPEPAPFCADNPSELPPSMERTGGIGDSRPPSFHPHASGGSQEN
LDNDTETDSLVSAQRERPRRRDGPEHAARLNGTTKGERRREPGGYDSSSTLMSSELETTSFFDS
DEDDSTSRFSSSTEQSSASRLMRRHKRRRRKQKVSRIERSSSFSSITDSTMSLNIITVTLNMEKYN
FLGISIVGQSNERGDGGIYIGSIMKGGAVAADGRIEPGDMLLQVNEINFENMSNDDAVRVLREIVH
KPGPITLTVAKCWDPSPRGCFTLPRSEPIRPIDPAAWVSHTAAMTGTFPAYGMSPSLSTITSTSSS
ITSSIPDTERLDDFHLSIHSDMAAIVKAMASPESGLEVRDRMWLKITIPNAFIGSDVVDWLYHNVEG
FTDRREARKYASNLLKAGFIRHTVNKITFSEQCYYIFGDLCGNMANLSLHDHDGSSGASDQDTLA
PLPHPGAAPWPMAFPYQYPPPPHPYNPHPGFPELGYSYGGGSASSQHSEGSRSSGSNRSGSD
RRKEKDPKAGDSKSGGSGSESDHTTRSSLRGPRERAPSERSGPAASEHSHRSHHSLTSSLRSH

HTHPSYGPPGVPPLYGPPMLMMTPPPAAMGPPGAPPGRDLASVPPELTASRQSFRMAMGNPS
EFFVDVM
>Mmus-Dvl2
MAGSSAGGGGVGETKVIYHLDEEETPYLVKIPVPAERITLGDFKSVLQRPAGAKYFFKSMDQDFG
VVKEEISDDNARLPCFNGRVVSWLVSSDTPQPEVAPPAHESRTELVPPPPPLPPLPPERTSGIGD
SRPPSFHPNVSSSHENLEPETETESVVSLRRDRPRRRDSSEHGAGGHRPGGPSRLERHLAGYE
SSSTLMTSELESTSLGDSDEDDTMSRFSSSTEQSSASRLLKRHRRRRKQRPPRMERTSSFSSVT
DSTMSLNIITVTLNMEKYNFLGISIVGQSNERGDGGIYIGSIMKGGAVAADGRIEPGDMLLQVNDM
NFENMSNDDAVRVLRDIVHKPGPIVLTVAKCWDPSPQAYFTLPRNEPIQPIDPAAWVSHSAALTG
AFPAYPGSSSMSTITSGSSLPDGCEGRGLSVHMDMASVTKAMAAPESGLEVRDRMWLKITIPNA
FLGSDVVDWLYHHVEGFPERREARKYASGLLKAGLIRHTVNKITFSEQCYYVFGDLSGGCESYLV
NLSLNDNDGSSGASDQDTLAPLPGATPWPLLPTFSYQYPAPHPYSPQPPPYHELSSYTYGGGSA
SSQHSEGSRSSGSTRSDGGAGRTGRPEERAPESKSGSGSESELSSRGGSLRRGGEPGGTGDG
GPPPSRGSTGAPPNLRALPGLHPYGAPSGMALPYNPMMVVMMPPPPPPVSTAVQPPGAPPVR
DLGSVPPELTASRQSFHMAMGNPSEFFVDVM
>Mmus-Dvl1
MAETKIIYHMDEEETPYLVKLPVAPERVTLADFKNVLSNRPVHAYKFFFKSMDQDFGVVKEEIFD
DNAKLPCFNGRVVSWLVLAEGAHSDAGSQGTDSHTDLPPPLERTGGIGDSRPPSFHPNVASSR
DGMDNETGTESMVSHRRERARRRNRDEAARTNGHPRGDRRRDLGLPPDSASTVLSSELESSSF
IDSDEEDNTSRLSSSTEQSTSSRLVRKHKCRRRKQRLRQTDRASSFSSITDSTMSLNIITVTLNME
RHHFLGISIVGQSNDRGDGGIYIGSIMKGGAVAADGRIEPGDMLLQVNDVNFENMSNDDAVRVLR
EIVSQTGPISLTVAKCWDPTPRSYFTIPRADPVRPIDPAAWLSHTAALTGALPRYGTSPCSSAITRT
SSSSLTSSVPGAPQLEEAPLTVKSDMSAIVRVMQLPDSGLEIRDRMWLKITIANAVIGADVVDWLY
THVEGFKERREARKYASSMLKHGFLRHTVNKITFSEQCYYVFGDLCSNLASLNLNSGSSGASDQ
DTLAPLPHPSVPWPLGQGYPYQYPGPPPCFPPAYQDPGFSCGSGSAGSQQSEGSKSSGSTRS
SHRTPGREERRATGAGGSGSESDHTVPSGSGSTGWWERPVSQLSRGSSPRSQASAVAPGLPP
LHPLTKAYAVVGGPPGGPPVRELAAVPPELTGSRQSFQKAMGNPCEFFVDIM
>Nvit-DSH
MEETKIIYHIDDEETPYLVKLNISPERVTLADFKNVLNRPNYKYFFKSMDDDFGVVKEEIVDDDAHL
PCFNGRVVSWLVSAEGSNVSDGASQCTDTVPHQDPKHDRVDHVTPGHTNRAQLSLSHEDTLTE
TESIISSRQGHHLHKSSRHHTDKYEKYNKYNGLRINGHSKHRSGMGYESASILSSDLETTTFLESD
DDASSRITSTTGRHTNMSSAVDRATLDRRRPQRRRRHRLPPMSRTSSFSSITDSTMSLNIITVSLN
MDTVNFLGISIVGQSNKGGDGGIYVGSIMKGGAVALDGRIEPGDMILQVNDINFENMSNDEAVRV
LREVVQKPGPIKLVVAKCWDPNPKGYFTIPRTEPVRPIDPGAWVAHTAAIRGEGFPPRPPSATTL
TSTSSSLASTLPDTERPFDELDLSVNTDMPTIVRAMARPDSGLEIRDRMWLKITIPNAFIGADVVD
WLHNHVKGFIDRRDARKYASLMLKAGFIRHTVNKITFSEQCYYIFGDLCSAMSNMKLDCDTVGPL
PPPSAWDMPYSGTYAPHSATGYSPMPFNFTNEPTVYGYHREESLHSGSGGSSAGSELMFKAP
MHDVKSCCSASESELQMPVVPAMPKSTATTVTTGGSGNGNGSGSNGKRSNGSRSRSSGSEQS
VQTGTGSGGGGGGSSVGPQQQQQQQDLSGSRQSFRIAMGNPCEFFVDVM
>Tcas-DSH
MDETKVIYHIDDEETPYLVKIPISPEKVTLSDFKNVLNRPNYKFFFKSMDDDFGVVKEEIIDDSAHL
PCFNGRVVSWLVSADGSNQSDGGSQCTDSVTHQSERVPPQAPESICTDTESIISSRQGRRHHKY
SSRINGHLPRVYETASIVSSDLETTSFQSETTGRHTALSECSSVSRLHVAGRKRPQRRRKQRMQ
AMSRTSSYSSITDSTMSLNIITVTLNMDTVNFLGISIVGQSNKGGDGGIYVGSIMKGGAVALDGRIE
PGDMILQVNDVNFENMSNDEAVRVLREVVQKPGPIKLVVAKCWDPNPKGYFTIPRTEPVRPIDPG
AWVAHTAAVRGDPVARPPSSSTVSSTSITSTIPANERFPDLEEPLTVNTPMATVVQAMQRPDSGL
EIRDRMWLKITIPNAFIGTDMIDWLLTHVDGFQERRDARKYASHLLKAGFIRHTVNKITFSEQCYYI
FGDLCSAMNNLKIQGDTDSVGPLPNVPNYMPYSGTYNPLEYMPMPFYTASENTVYGYNREESVL
SGSGGSSNGSDHLKDAAAGHSSASDSDLTSLGPRSALPMATGNGNGSSNGSDQSSGTQVAAQ
SKDIAGSRQSFKIAMGNPCEMFVDVM
>Chem-Dvl
MAEKETKIIYHVDDEETPYLVKIPKPPDQVTLGDFKSVINRPNFKFFFKSMDDDFGVVKEEIIDDDA
PLPCFNGRVVSWVVPPEDGSCDGQSQHSGDGIFVPVQSSNSNISRSSTMRSKERVQDSDAESIV

SRRSSRSRSSRKYESDADRRSRRSHREHRNYDQYDSASMMSSDLETTSFVDSEEESQMSSATE
SSRYVGGNKRRRRRRQRMPRVERCSSFSTITESTMSLNIITVTLNMDKINFLGISIVGQASKKGDG
GIYVGSVMKGGAVDADGRVEPGDMILAVGDVNFENMSNDDAVRVLRECVHKPGPIQLTVAKCW
DPNPKGYFTVPKDDVTRPIDPAAWVQHSEAMRAGGGLMGRGSPSMSTMTSTSDSFSSSIPEAD
RYLEHDLGLTLTIDTDMLTIVKVMNQENSGLTVRDRMWLKITIPNAFIGSDLVDWLFANVEGFQDR
REARKYASKLLKANLIRHTVNKVTFSEQCYYVFGDLSDRRRGMHMQDIEGSEEDTLAPLASTSAQ
AQMGGYIQQPYGMAMIPGGMMVTGAPPAYQQVVQPGMYAQYPYVAGSIAPSHLSGGSGGSGS
QKSQKIHDDQATVRSSGSSDRSHSSKGSRSRKPMLDDRSSLSSFHSDTISLRSEIIAPDNRSISSH
HSIPPMMVMPPGVAYSAAPVGLTPEQQQHIQVQMHQQQQMHHQIQQQLQQLQLQSPAQTPQP
PPAGSPQPPQDGTRTPAEQHRELGRLDTLPASLSASRQSFRMAMGNSSNEFFVDVM
>Aque-Dvl
MEETKVIYYVDDEETPYMTKIPLSPSKVKLSDLKEQLSRPGPFKKFFFKSIDDDIGVVKEEIIEDSAL
LPTAKGRIVCWVVSEGSASGSDVASKDLETIKEKDDDEASIVSGVSKSSRHSTSSRRHHHHHHH
RHHRRRHDRHKQPGSVTDYETATELTATDLEASCYETEDTASRLSVETTSTVTSHKHPRKKVLT
KKKVPRSMSMSTMTTSTMSDASMDILTVTLNMDAYNFLGISIVGHANDDGVGGIYVGTVMKGGA
VAADGRIETGDMLLQVNDISFENMSNDDAVRTLREIVQQPGPIILTVAKCLEPEAYAPMFEPRLEPI
RPLDPSAWVMHTNAQRAGDYGRPFTSSPTMSTMTSNSSPSLASSIPESERDLVKLNLTSPLYRV
AKAMAAPDSGLEVKDRMWLKMPIPKSFIGSDMVEWLHNNVEGFIDRRHARKYAAIMLKQGFVKH
AVNKYTFSEQCYYIFGDFKKAAANNLSTELTQLHLSDNSGGETDTLGPLPSDRNNGQGWSSSDD
QISLPTIEGSFYPHLMHSHSNSPEGQMQHHTGSSASSSSAAGSGYAHYPPRQHSGSPSDPRQY
PSPRPSHSHMELPPQMRMRHSNSISSASEFTSVSQQQYHHPGNGGAMGGASLPPDRMSLRSL
NIGGQVPPGVSASRASFQQALDNPCEYFIDVM
>Cint-Dvl
MSDETKIVYYLGDEQTPYVSKINLPPDSITLGDFKAAIKKINYKFFFKSTDADFGVVKEEVTNDKSIL
PLCDNRIVAWLKAPDMDSVSQCSLNSNTLPMQDKNEDMQLRPNSTNEGELSIENKRVSPHYPLY
NRYGSQSTLMSSDIETTSWDSRDDMSEYSTCTDMTASSRRKPRPKRQRYKKKRPRPGSDTTST
YSSSITDSSMSLNILTVTLNMEKYNFLGISIVGQTNDKGDGGIYIGSIMKGGAVAADNRIEPGDMLL
QVNEVNFENMSNEDAVRVLRNIVHKPGPITLTVAKCWDPNPDNYFTIPKDEPVRPIDPAAWANHI
MTVKGDMHGPSPPYSLNPESSSASSLPESERYDMPLSTCTDMGAVVKSLKMPDSGLDVKTRM
WLKITIPNAFIGSDLVDWLQQKVHGLTERRDARKYASSLLKAGYIRHTVNKITFSEQCYYVFGDYT
GTLDKDMSNLSLVESNSDRDSDTLGPLHGHHNTQPWMHQPSTSNHGYASYSQYPYTQSGQLS
VGPPIPLNNGISSMVDYSSPPPTYPGLASHHSDGDNLSVHSAHSLHRAASEASTYRQHLATFGAN
GNNSAGSESESRYSSRSGKSGSGGSAKPRSRSGSEKSSDVLHQQMAYGGSTSYHNSLQRSNH
PNQRTNQHSRPLSAIPPNLSNSQQSFQQAMGNPCDYFVDVM
>Smed-DVL-1
MEETRIIYYVDDEETPYLIKFHSPPEQITLGDFKNALNRPNYKFFFKSLDDDFGVVKEEITDDDAKL
PYVNGRVVSWLVVSEGSTQSDNHSSSGKEVLLVDSDKSKDKGTVSDSSDPKSPSFRNYNKIPTK
HSSSSKKQEESNKIHRQNHKFTGPEKITLDETDDAFDEIDSIYNEDKVPPLRKFSDFKHSVKLKKL
RNAQGSHGNSNSSSNNNNSNSNNASNNAPAKQQPIYESSSSMMSSDLDTTSFFDSEDDSSRFS
SATETTMSSKYGKQRRQLRRRRKMPHLSRASSFSSMTDSTVSLNIITVTLNMDTVPFLGISIVGQT
NGNQENGDGGIYVGSIMKGGAVALDGRIEPGDMILEVNGISFENVSNEEAVRTLREQVQKLGPVT
LVVAKSWDPNPTGYMLPQQDPVRPIDPRAWVLHTQAMGNMAPPNQPPVASGDQFIQSGKYLPY
AGAMSTVASTITTTSSSLKSRTDETIPSPLTTNHEPSVIIRAMAQADSGLPIRDRLWLKITIYNAFIGS
DLVDWLYSHVQGFTDRKDARKFATNLLKMGFIRHTVNKSSFSEHQRLTRFRLLPANSNPKRDWC
IKILAVRSRQFSHRDSSLLAVTNGQVTISSRILVQKCIHKHKS
>SmedDVL-2
MTNCATSGNVISDETRIIYHIDEEETPYLIKLSISPDKVTLGDLKNTLNRPHYKYFFKSMDDDFGVV
KEEITDDEAKLPCFKGRVISWLVTAEGSTVSDNVDSNGILDKNESRMLPFQESHFPLINNIKASGG
TTTNESDTICDTCTDTDSVYSAAQDRVGPPRSFHDYKQAGRVAAHANRVNTNTPNGQNPIYETN
SSMMSSDLESTSFFDSEDESSRFSTTTCTTMSSRYGRQKQQRRRRRPPAISRASSFSSITDSTM
SLNIVTVRLNMDTVKFLGISIVGQSNKGGDGGIYVGSIMKGGAVAQDGRIEPGDMILEVNDISFED
MSNDDAVRTLREQVQKPGPINLVVAKCWDPNPKGYFTIPRQEPVRPIDPRAWVLHTNAMTAGAS
EPPSSVNGVHPQVSNLVAPSMQSLLSGGTMLAGTSAATFNAAAFGYMPQPQNINQNTASVSTV

GGPPGASVGFFGYPMGMPGQFSQGAGSIVTTSSSLPESERYQEELHLTKNTDVGTILRVLSQPD
SGLDIRDRLWLKITLPNAFIGSNLVDWLYRHIEGFSDRKEARKYAANLLKFGYIKHTVNKVTFSEQ
CYYVLGNTTLNMSRLSLDQVESVSEVGVNGPHHLAALPPPNFSSNKQPISSCINQPPLNINPQLT
ATSEPLPSNNANVATATASSNSQYSVVGPLPCSQPSQHASSNASASAIKKSGSCNSLSGSSSST
SSSSSSNRSNTRINGNASSVSNMISKNPPPKIPPRTIASVSTNSTNPIISGFQNRGQSSVSQ
>Xtro-Dvl1
MAETRIIYHIDEEETPYLVKLPVPPEKVTLADFKNVLSNRPVHHYKFFFKSMDQDFGVVKEEISDD
NAKLPCFNGRVVSWLVLAESSHSDGGSQSTESRTDLPLPIERTGGIGDSRPPSFHPNASSSRDG
LDNETGTDSVVSHRRDRHRRKNRETHDDVPRINGHPKLDRIRDPGGYDSASTVMSSELESSSFV
DSDEDENTSRLSSSTEQSTSSRLIRKHKRRRRKQKMRQIDRSSSFSSITDSTMSLNIITVTLNMEK
YNFLGISIVGQSNDRGDGGIYIGSIMKGGAVAADGRIEPGDMLLQVNDVNFENMSNDDAVRVLRE
IVSKPGPISLTVAKCWDPTPRSYFTIPRAEPVRPIDPAAWITHTSALTGAYPRYEQEDSPLSVKSD
MATIVKVMQLPDSGLEIRDRMWLKITISNAVIGADVVDWLYTHVEGFKERREARKYASSMLKHGY
LRHTVNKITFSEQCYYVFGDLCGNVAALNLNEGSSGTSDQDTLAPLPHPAAPWPLGQGYSYQYP
LAPPCFPPTYQEPGFSYGSGSAGSQHSEGKSTYSGVFLP
>Xtro-Dvl2
MAETKVIYHLDEEETPYLVKVPVPANEIRLRDFKAALGRGHAKYFFKAMDQDFGVVKEEISDDNA
KLPCFNGRVVSWLVSSETSQTDSAPPAAEVRPDPPPVPPPVPPPPAERTSGIGDSRPPSFHPNV
SGSTEQLDQDNESVISMRRDRVRRRDSTEQGVARGVNGRAERHLSGYESSSTLLTSEIETSICDS
EEDDAMSRFSSSTEQSSASRLLKRHRRRRKQRPPRLERTSSFSSVTDSTMSLNIITVTLNMEKYN
FLGISIVGQSNERGDGGIYIGSIMKGGAVAADGRIEPGDMLLQVNDINFENMSNDDAVRVLRDIVH
KPGPIILTVAKCWDPSPQGYFTLPRNEPIQPIDPAAWVSHSAALSGSFPVYPGSASMSSMTSSTS
VTETELSHALPPVSLFSLSVHTDLASVAKVMASPESGLEVRDRMWLKITIPNAFLGSDMVDWLYH
HVEGFQDRREARKFASNLLKAGLIRHTVNKITFSEQCYYIFGDLTGCENYMANLSLNDNDGSSGA
SDQDTLAPLPLPGASPWPLLPTFSYQYPAPHPYSTQPPAYHELSSYSYGMGSAGSQHSEGSRS
SGSNRSDGGRGTQKDERSGVVGVGGGESKSGSGSESEYSTRSSIRRIGGGEAGPPSERSTSSR
PPLHHPPSVHSYAAPGVPLSYNPMMLMMMPPPPLPPPGACPPSSSVPPGAPPLVRDLASVPPE
LTASRQSFHMAMGNPSEFFVDVM
>Xtro-Dvl3
MGETKVIYHLDEQETPYLVKLPVPAEKVTLGDFKNVLNKPNYKFFFKSMDDDFGVVKEEISDDNA
KLPCFNGRVVCWLVSADGSQSDAGSVCADNQSDLPPPIERTGGIGDSRPPSFHPNTRGSQENL
DNETETDSVVSAHRERPRRKETPEHATRLNGTSKMERRRDTGGYESSSTLMSSELDSTSFFDSD
EDDSTSRFSNSTEQSSASRLMRRHKRRRRKPKAPRIERSSSFSSITDSTMSLNIITVTLNMEKYNF
LGISIVGQSNERGDGGIYIGSIMKGGAVAADGRIEPGDMLLQVNDTNFENMSNDDAVRVLREIVHK
PGPITLTVAKCWDPSPRNCFTLPRSEPIRPIDPAAWVSHTAAMTGSYPAYGMSPSMSTITSTSSSI
TSSIPETERFDDFQLSIHSDMVTIVKAMRSPESGLEVRDRMWLKITIPNAFIGSDVVDWLYHHVEG
FTDRREARKYASNLLKAGYIRHTVNKITFSEQCYYIFGDLCGNMANLSLNDHDGSSGTSDQDTLA
PLPHPGAAPWPIAFQYQYPLPHPYSPHPGFPDPAYSYGGGSAGSQHSEGSRSSGSNRSSTEKR
KEREAKGGDTKSGGSGSESDHTTRSSVRRERAASERSVPASEHSHRSHHSIAHSIRSHHTHHSF
GPPGIPPLYGAPMMMMPAPASVIGPPGAPPSRDLASVPPELTASRQSFRMAMGNPTKNSGVFD
FL
>Cang-DSH-1a(CAN07318)
MDTVNFLGISIVGQTSTRGDNGIYVANIMKGGAVALDGRIEAGDMILQVNDISFENFTNDQAVDVL
RDAVSRRGPIKLTVAKSFENGPQSCFTIPRNSREEPVRPIDTQAWIQHTNAMRGMPSIVEGVEGA
PTPLPGDWPPHANGRPQSSSTVTSNGSNGQNTVVGGGNGTQIKLDLATDKKKIVEVMAMPNSG
LDIKNRTWLKIPIPMSFLGSDLVEWLLDHVEGLRERKDARKFAAELLKLKYIAHVVNKITFTEQCYY
VLGDECSALLMLNWNFCWKIHEN
>Cang-DSH-1b
MPERKIGGGGSLVTTIVSRFESKNLSGGPATKKSPQPPIAKLDDDFDAAPPPKSKVLHQLKRTYT
NLPTEEILSKKQLKKRQSESALQHLGGNKNKEGGAGQMFIGTLMRLASLKSLVASVEDIQIGEGK
KKDQKKKRKRKRADAKEALKNRLLNWNSVEEPAPSWVNRSPSPSKIHIPTPVFKRSPEVEEEPE
GLKKSSSRSLFSFSRSESKESLLSQKTSPRSLSPARNLAENIKKKIWGRSDSLGSQASLGSAKSP
RSLSPARNLVNRFLGKSASQTSIRSQETLKERSSSLKSKQEGSEVEGRSGSLRSLSKKILGSNLKL

EARPSEPNISSPVATSAPTSAPAATPAPKRRLVRSDATIGARLKKKLSVEEDEPTEPEPLPKSPLR
SPLPKLVIEHLPDRPQVTQRSLSEIVSTDRLRPLSLPAGRRKLPPMARTMSLIPPPVLPPLYEEESL
AETSHNSLGTTTHTKRRYGGSNTTSTYQGVKDAAPDASPRRHLIGFLHRTSHLSLTSELSGNASF
YLIDLGTELEPEVGSETFGAQHQLSRRDLSSYFCSSGHPGSKKKTCPIGRHDRSQAQEEAVGHR
RQHLEDSTIASESDARIFSDEDDR
>Cang-MIG-5
MEPQGSSDQIKVFYFLDDESTPYVSVIESRGGIVTLDDFKNSFTKRGYKYFCKELDPDIQCEVKVE
LTEGTDRLRKSQNGFYEIFLVSTPGYGTLPRNSGTLTRKNVKGTLDRRRRRSADFDAVPYSDASL
APSTIVSRRAGEHLAELYTSNSEDPYQYDDSTRLTGESSLYEPLATRDMNKMYEDDRKQKRKIKK
ERHHRRPYVPSTISSATESSAGSGLPRILEVFLPMKNVPYLGLNVCTIDGHIFVSEIAQNGAVERD
GRIDVGDQILQVNRISFEELSVPQAVRALREAAASRKPMTLYVSKFSKAAGSEXXXXSSYIANIAV
RALREASRKPMTLYTYLL
>Cbri-DSH-1
MAESPPRVDSSAPDVGDPTSMMERLRLGDDGGKDDEFDNKSVSSAQYSQASEATTAVKQQPF
LHTMTKVYCHIDDETDPYMLEVHVPPDLITLGDLKRVLMRTNFKYYRKALDPDSGYEVKAEIRDD
SQRLTPSSNNLFELFLLTIEGSTHSDGSSGKMRKYPSVPGPAPSNRSGPPMNYQHAAYQFDNSM
MSTDSESMISAAIPGYLKSAAYNRRFPQHYLAMSIKLGVARERAIPASSNTPSTSSMTTSSDVLRS
LLFTAKERKVLSTYTAPPSHRNHSPGVVSQLINKIEQKASPPTTSSRTKKKKKAPPLAQIDDLDLIE
DEKTVVEVAKSPRRKKYTGNPLGNLKILNGFLPSSKTTDDKENDSVPSTGKKLKKSKSKSRVEPE
TDPPFRLKRTLTDLTNNHTPSGSTWRQMVSNALGGPLKKRLSEGALFFPGTSKAADDCVDEEG
GNTKTKTKSPFLQRRKQNEKSAGGANRKESGGSASSSFFGALIRLSHSAASLTSLTSLGGSRSNS
ASPSSSRSNTKEFKEELKPPQPPPPDLISPAAALSKPIFSLAPPSSPVSLTPPSRELRKSKTCQITT
DRPPIIPSITISESRSLNRIDRCRPVTVDGSNLTPDRRPLVSRRSTMSRTMSLIPTSPSLPPLYEEEM
ASTAAMVDEEREEKTQKRRMRRYGGSNTTSTYQGRKDAAPDASPRRHLIGFLHRTSHLSLTSEL
SADASFYLIGHRRHLEESTIGSESDARVFSDDDDRGSTTTDFTSVSRQHEKMAKKKKNKRNFRK
PSRASSFSSITESSMSLDVITVNLNMDTVNFLGISIVGQTSNCGDNGIYVANIMKGGAVALDGRIEA
GDMILQVNETSFENFTNDQAVDVLREAVSRRGPIKLTVAKSFENGQSCFTIPRNSREEPVRPIDT
QAWIQHTNAMRGMPSIVEESAPTPIPGEWPHGRPPSSSTVTSNGSNGQNTVVGNGAHIHLDIHT
DKKKVVEIMAMPGSGLDIKNRTWLKIPIPMSFLGSDLVEWLLDRIEGLRERKTARNYAADLLKLKYI
AHVVNKVTFTEQCYYVLGDECADYARFRNEDGGPKYQWTMGMNGMSAGNGSSVMLPPPQFP
GAVPFKGMAPSMVSDGESRMYVMHI
>Cbri-DSH-2
MTDSPSPIDSSFDASDVATPCTVIAAKSSIRNFRDLEEGDDFDDDDDGIEDQTDYTESFPQPEDR
ALDGEDPSNIYVDDLSKEFSGCASSVMEPLPKPLTFARTMTKVFYHLDNDPVPYTIDVHVPPDCM
TLRDVKRKLPRTNYQYYCIALDPDSGKEVRAEIRDDSQRLYPLRSGEFRLYMLTIEGSVHSDTSS
GRHRRKNKGGSSNGSSSSRDYLRAARDYDNQSESHAVLSRYFTFILSAPFTDDESQVSETPVYV
KKANAFNRRQASQAYDQYQPRHMLHARHHQNHYEDSTFDVTSESESQFRSGALYDEDVDDAQ
SINTDLTSVSQVQLKKRWRQQQKEMRNKWKRMPSMSTASSSLSSITESSMGLEVITVRLNLQSM
PLGMIPYGLKTARGGDAGLYVGDILGRGAVALDGRIEVGDMISEINEIDLSNYSNEAAAQLLKDAV
APRQFVTLTIAKSLDSRKAAAAASARNTRNEPIRPIDTNEWIKHANAMKGMPSISEESCSTPIPDD
WPTNSSASGTPFGGPHPAIAHMTTETNKKRIMEILAAPGSGLEIKDREWMKLPLKMCFLGKDLVN
WLLDHVEGLRKHKEAKQFAKEMWKLGYIVDALGQNVFSENCYYKMGEECADYTQLRAPDGGFK
YAQSHTSSASGHSSNNNIFPPSMYPPQMKPMPSTAGLNAHHRNSAVLNSMVSGYASMPSSPFP
NVKPSAVDCGRTRDDVRSQTSGSSQGSSRRYVELPRKPSSLGSGSGISDQMNIDRVASRSSFR
AAMSGSLRHFSID
>Cbri-MIG-5
MEPPCSSDSNQIKSKQDGFQVFYYLDDETTPYVSVIDAREGVATLGNFKNSFTKRGYKYYAKEL
DPDIQREVKVELISDTDRLRRSQNGFYEVFLVSTPGYGTLPRNTGTMTRPQRAAIDKRRRRSADF
DATPYSDASLAPSTIVSRRAGEHLAEMYTSNSEDPYQYDESTRRTTDDSSMYEPLTARDMNRYH
DDDRRKKKQKKDRFRRPYVPSTISSATESSVNSGLPRILEIYLSMKNVPYLGLSVCTMDGHIFVSE
IAPEGAVEKDGRVNVGDQILQVNRVSFEDLTGPQAVRALREAAGSKRPITLYISKYRRAAPSEYD
DPLASMASETMPLDVGVWVETAVQATEKMKALGIDPQEQTMTSVDDGTMPFTSTASDDEERILY
DQRRNGIPRALLEEAERKKENERNEKAEQLTELIDPIIVVRAMARPDSGLVIKNRKWLKISVPMSFI

GQDLIDWLVDHMTDIHNRKRAKLYAARLLAAGLIRHVVSKLTFTEKCYYVFGDGILPTDRTSADNS
GTSGTTTTRVEATTEVTYVGSPAPHALANRMGRTIPPHRLETTTLSPVAHDQTWLRRRRDCESP
MTNDYASMVGESQIGMNPAGNYYGAKNSRQMMVPASSQVTSSSLTNGSGGIGGPPPTPLSSTM
VLAASPIQS
>Cbre-DSH-1a
MAEPPPQVDSTNAPDIGNPTSMMERLRLRDQTEDGGENDDFDNKSVSSAQYSQASEATTAVKQ
QPFLQTMTKVYCHIDDEPDPYMLEVHVPPDLITLGDLKRVLMRTNFKYYRKALDPDSGYEVKAEI
RDDSQRLTPSSNNLFELFLLTIEGSTHSDGSSGKMRKYPSVPGPAPSNRNGPPMNYQHAAYQFD
NSMMSTDSESMISAAIPGYLKSAAYNRRFPPQQHYLGHRRHLEESTIGSESDARVFSDDDDRGS
TTTDFTSVSRQHEKMTKKKKNKRNFRKPSRASSFSSITESSMSLDVITVNLNMDTVNFLGISIVGQ
TSNCGDNGIYVANIMKGGAVALDGRIEAGDMILQVNETSFENFTNDQAVDVLREAVSRRGPIKLT
VAKAFENGQSCFTIPRNSREEPVRPIDTQAWIQHTNAMRGMPSIVEGSDLVEWLLDRIEGLRERK
TARNYAAELLKLKYIAHVVNKVTFTEQCYYVLGDECADYARFRNEDGGPKYQWTIGMNGMSAG
NGSSIMLPPPHLPGPPGAFKGMAPSMVSGYASMPSSPFPAQLQQQMQNHRREGSTTSGSSGG
GIRKQRVVLPRQPPPSANGNVHYDDSSTIYEGSNNSFLAATGQRYEFN
>Cbre-DSH-1b
MSIKLEARKTRALPTDSSSPSTSMSTSSDVLRSLLFTAKERKVLSTYTAPPSNRHHSPGVVSQLIN
KIEQKTSPPSTSSRPKKKKKAPPLAQIDDLDIIEDEKVEEVVKSPRRKKYTGNPLGNLKILNNILPKA
TSSEDKIDKEDKENSSHPTTGKKLKKSKSKSRVEPEIEPPFRLKRTLTDLTNNHTPAGSTWRQMV
SNALGGPLKKRLSEGALFLPGPSNCGDEEGEKTTTTKQRRFLQRRKQSEKSAGDGKESSGGRG
GSASSSFFGALIRLSHSAASLTSLTSLGGSKSRSTSASPSSSRSNTKEFKEEIKPPQPPPPDLISPA
AALSKPIFSLASPPSPVELAPPSRELRKSKTCQITTDRPPLIPSITISESRSLNRIDRCRPVTVDGSG
LTPDRRPLVSRRSTMSRTMSLIPTSPSLPPLYEEETASTAAMVEEERLDKVQKRRMRRYGGSHT
TSTYQGRKDAAPDASPRRHLIGFLHRTSHLSLTSELSADASFYLIGHRRHLEESTIGSESDARVFS
DDDDRGSTTTDFTSVSRQHEKMTKKKKNKRNFRKPSRASSFSSITESSMSLDVITVNLNMDTVNF
LGISIVGQTSNCGDNGIYVANIMKGGAVALDGRIEAGDMILQVNETSFENFTNDQAVDVLREAVSR
RGPIKLTVAKAFENGQSCFTIPRNSREEPVRPIDTQAWIQHTNAMRGMPSIVEESAPTPIPGEWP
HGRPPSSSTVTSNGSNGQNTVVGGGAHIHLDIHTDKKKVVEIMAMPGSGLDIKNRTWLKIPIPMS
FLGKVFMIDKPFSINFFLGSDLVEWLLDRIEGLRERKTARNYAAELLKLKYIAHVVNKVTFTEQCYY
VLGDECADYARFRNEDGGPKYQWTIGMNGMSAGNGSSIMLPPPHLPGPPGAFKGMAPSMVSD
GESRMYVMHI
>Cbre-DSH-2a
MTDSPSPIDSSFDISDVGTPATVIHKSIFRKEAEEEDFDLETDEDYTEQYREHDGVDQSELSSSFL
VDDYSKDCDSSISAPIPKPSFFRTITKVYYHVDDENIPYTADIHVPPDCITLGDVKRKLPRTNFKYY
CIALDPESGLEVKAEVRDDSQRLYPLRDGRFVLYLLTIEGSVHSDTSSGRHRKNKLSSKGSNSSR
EYLKAAHHFDNPASYSDSESQASSIPAYFKKAKAFNKRQAFQAHDRHHHHQLPRHKPHGRHHH
NHYDEESTFDITTESDDHYRDGITYYDEDEDDSRSINTDLTSVSQVALKAKWRQQQREMRNKYK
RMPSTASSTLSSITESSMGVEVITVRLNIQEFPIGMVPSILTTARGDDGGLYVGQVNPRGAVALDG
RIVVGDMISEINNIDLSNYSGKEAVNILKQAVTNQPYITLTVVKTGENKKAAPAVLRNPRAEPIRPID
TNEWLKHATNAMKAMPSISEESCSTPIPDDWPTNSSASGTPFGGPPPNIHCLTVTTDKKDLVQA
MMAPGSGLEIKNHEWLKILIPMSFLGKDLVDWLLDHVQGLKNRDDSCKYAGKMLKEHYIVQPNG
KKKFSENCYYVVGEKCGDYTSLRGNDGEYKYAQSQTSSASGHSSNNNVFPPSMYPPPLPPSAL
GAHHRNSAVLNSIGSGYASMTSSPLPSEKPSNCGRTRDDQRSQTSGSSRGSSRRYVELPRKPS
SLGSGSGVSDQINLDRVASRSSFRAAMSGSLRQFNIDS
>Cbre-DSH-2b
MTDSPSPIDSSFDISDVGTPATVIHKSIFRKEAEEEDFDLETDEDYTEQYREHDGVDQSELSSSFL
VDDYSKDCDSSISAPIPKPSFFRTITKVIIFVKVEFFNWVLGCITKSDENTAKTHLSAKEPTFFCVEL
GPFLTELFSWSIETSKKKVVTENSKSSNKTFFPRIELFYLNFQVYYHVDDENIPYTADIHVPPDCITL
GDVKRKLPRTNFKYYCIALDPESGLEVKAEVRDDSQRLYPLRDGRFVLYLLTIEGSVHSDTSSGR
HRKNKLSSKGSNSSREYLKAAHHFDNPASYSDSESQASSIPAYFKKAKAFNKRQAFQAHGTQPL
SLFSSFVSFSDRHHHHQLPRHKPHGRHHHNHYDEESTFDITTESDDHYRDGITYYDEDEDDSRSI
NTDLTSVSQVALKAKWRQQQREMRNKYKRMPSTASSTLSSITESSMGVEVITVRLNIQEFPIGMV
PSILTTARGDDGGLYVGQVNPRGAVALDGRIVVGDMISEINNIDLSNYSGKEAVNILKQAVTNQPYI

TLTVVKTGENKKAAPAVLRNPRAEPIRPIDTNEWLKHATNAMKAMPSISEESCSTPIPDDWPTNS
SASGTPFGGPPPNIHCLTVTTDKKDLVQAMMAPGSGLEIKNHEWLKILIPMSFLGKDLVDWLLDH
VQGLKNRDDSCKYAGKMLKEHYIVQPNGKKKFSENCYYVVGEKCGDYTSLRGNDGDYKYAQS
QTSSASGHSSNNNVFPPSMYPPPLPPSALGAHHRNSAVLNSIGSGYASMTSSPLPTVLNSIGSGY
ASMTSSPLPSEKPSNCGRTRDDQRSQTSGSSRGSSRRYVELPRKPSSLGSGSGISDQINLDRVA
SRSSFRAAMSGSLRQFNIDS
>Cbre-MIG-5a
MDAPCTSDTHQIKVFYYLDDETTPYVSVIDTREGVATLGNFKNSFTKRGYKYYGKELDPDIQREV
KVELISDSDRLRKSQNGFYEVFLVSTPGYGTLPRNTGTMTRPQRAALDKRRRRSADFDATPYSD
ASLAPSTIVSRRAGEHLAELYTSNSEDPYQYDEHTRRTTTDDSSMYEPLAARDMNRYHEEERRK
KKQKKDHRFRRPYVPSTISSATESSVNSGLPRILEIYLPMKNVPYLGLSVCTIDSHIFVSEIAPEGAV
EKDGRVSCGDQILQVNRVSFEDLTATAAVKALRDAAASKRPITLYISKFVRGAPSEYDDPLASIAS
ETMPLDVGVWVETAVQNTEKMKALGLDPQEQTMTSVDDGTLPFTSTASDDEERILYDQRRNGIP
RALMEEAERKRENEQNEKIEQLTELIDPIIVVRAMARPDSGLVVKNRKWLKILVPNSFIGRDLVYW
LVDHMTDIHSRKHARLYAARLLAAGLIRHVVSKLTFTEKCYYVFGDGILPPTATVNDRNSTDTSGT
SATTMRVEATTEVTYVGSPAPHALATRIGRNIPPHRLETTTLSPVAHDQTWLRRRRDCESPMTND
YASMVGESQLGMGMNTGNYHGYVAKNPRVVPAPSQVTSSSLTNGSGGIGGPPPTPLSSTMVLA
AVPSPIQSSPNVALLMHDFDAENNSGNSKSSRILRA
>Cbre-MIG-5b
MTRPQRAALDKRRRRSADFDATPYSDASLAPSTIVSRRAGEHLAELYTSNSEDPYQYDEHTRRT
TTDDSSMYEPLAARDMNRYHEEERRKKKQKKDHRFRRPYVPSTISSATESSVNSGLPRILEIYLP
MKNVPYLGLSVCTIDSHIFVSEIAPEGAVEKDGRVSCGDQILQVNRVSFEDLTATAAVKALRDAAA
SKRPITLYISKFVRGAPSEYDDPLASMASETMPLDVGVWVETAVQNTEKMKALGLDPQEQTMTS
VDDGTLPFTSTASDDEERILYDQRRNGIPRALMEEAERKRENEQNEKIEQLTELIDPIIVVRAMARP
DSGLVVKNRKWLKILVPNSFIGRDLVYWLVDHMTDIHSRKHARLYAARLLAAGLIRHVVSKLTFTE
KCYYVFGDGILPPTATINDRNSTDTSGTSATTMRVEATTEVTYVGSPAPHGLATRIGRNIPPHRLE
TTTLSPVAHDQTWLRRRRDCESPMTNDYASMVGESQLGMGMNTGNYHGYVAKNPRVVPAPSQ
VTSSSLTNGSGGIGGPPPTPLSSTMVLAAVPSPIQSSPNVALLMRDFDAENNSGNSKSSRILR
>Cjap-DSH-1a
MADPPVDELEKLENLRIADDPDSKEDDFDTKSGSSAQYSQASEATTAVKQQPFLQQTMTKVYCH
IDDETDPYMLEVHVPPDMITLGDLKRVLMRTNFKYYRKALDPDSGYEVKAEIRDDSQRLAPSPNN
LFELFLLTIEGSTHSDGSSGKLRKYPSVPGPAPSNRNGPPMNYQHAAYQFDNSMMSTDSESMIS
AAVPGYLKNAYNRRFPAQYLELKVQFFDLITKYRTTRQNRVISRVSQRCSL
>Cjap-DSH-1b1
MSNHGSTRHSDATLRALKFTAKERKVLSNYTAPPPNRHHSPGVVSQLVNKIEKKSSPPLASSKR
KKIPPLAEIDDLEMAVEDVVEVVKSPRRKKYTGNPLGNLKILNHLLPGTSKDPKDEVPIKKLPENK
ENTATQTPTPSTGKRLKKSKSKGRVEPEDERFFRLKRTMTDNTNNHSKSSPTGSGGWRQIVSSA
LGGPLKKRLSEGALLPSTSSGSMYPGGEKKSKRGRLQRMKSAEGSASSSFFGALIRLSSSAVSLT
SLTSLGSLGSNKSKPHSPSNTKEFVESLRRSPPPPTLVSESDSVQLPTQPPPPLRKSKTCQNTPD
RPPIIPSITITESRSLNRIDRCRPITVDGSGLTPRSSRRSAMSRTMSLIPTSPSLPPLYEEETASTAA
MVDEERIAEKKHRMRRYGGSNTTSTYQGRKDVAPDASPRRHLIGFLHRTSHLSLTSELSGNASF
YLIGHRRHLEESTIGSESDARVFSDDDDRGSTTTDFTSVSRQHEKMAKKKKRNIKNFRKPSRASS
FSSITESSMSLDVITVNLNMDTVNFLGISIVGQTSTGGDNGIYVANIMKGGAVALDGRIEAGDMLLQ
VNDVSFENFTNDQAVDVLRGSTTTDFTSVSRQHEKMAKKKKRNIKNFRKPSRASSFSSITESSMS
LDVITVNLNMDTVNFLGISIVGQTSTGGDNGIYVANIMKGGAVALDGRIEAGDMLLQVNDVSFENF
TNDQAVDVLREAVSRRGPIKLTVAKSFENGAQSCFTIPRNSREEPVRPIDTQAWIQHTNAMRGM
PSIVEESAPTPIPGEWPHGRPPSSSTVTSNGSNGQNTVVGGGAHIHLDVHTDKKKVVEIMAMPG
SGLDIKNRTWLKIPIPMSFLGSDLVEWLLDHIDGLRERKTARNYAAELLKLKYIAHVVNKVTFTEQC
YYVLGEECADYARFRNEDGGPKYQWTIGMNNGVAASNGGSVMLPPPHLSGPGAHFKGMAPSM
VSDGESRMYVMHI
>Cjap-DSH-2a
MTDSPSPIDSSFDASDIATPCTVIAAKTRNLRDLKIEEEDGEDSSHGDEEEVSAIYVDDFSKTDFSE
GESSVMEPLPRPPSFARTITKVYCHMDNEEVPYMVEVHVPPDCITLRDVKRKLTRTNYKFFCIAL

DPDSGLEVKAEIRDDSNKLYPLKDGRFQLFLLTIEGSVHSDTSSGRHRKQKTSSKGSSSSREFRA
GYEHASVMSEVSSDASSLPTYVKKAHAYNRRHGAPPQYGDLRQHLLQKQRHRNIPYQQQQNPY
EESSFDVTESDVYGGGHHHHHQHRDGETFYDEDDDSRSINTDLTSVSQQHLKKMYREQQARAQ
NKWKSAMSTTSSSFTDITESSMGVEIITVRLNLETLPLGMIPCGDTDSRGDSGLFVGSITDRGAVA
LDGRIDIGDMILEINGVSLQNHTNQQAANLLKLRPLPCYFLL

>Cjap-DSH-2b
MHLESAVQRQFLTLTIAKTDKKKTAFLRNTRNEPVRPIDTNEWIKHATQHMKAMPSISEESSSTPI
PDEWPSHSSASGTPFGGPTPTINQLSVITDKKYVVEVMAAPGSGLEIKDREWLKIPIPMSFLGRDL
VDWLLDHIKGLTKREEACNFAGEMLKMGYIQHVVNKKHFSEKCYYVMGEECADYTQLRAPDGG
FKYPQSRESSTSNSTTNNNNNNVFPAHMYPPNQNEVQSANSSQNHQRNSVVLPNGIPMQQSVS
GYASMPSSPFPPKNGGDCGRTRDDQRSQTSGSSRGSSRRYVELPRKPSSQGSGSAHENSLMD
RVASRSSFRAAMSGSLRQFNIDG

>Cjap-MIG-5
MEQPCTSESSQIKVFYYLDDETTPYVSVIDTQDGVATLGNFKNSFTKRGYKYYGKELDPDIQREV
KVELTLDSDRLRRSQNGFYEVFLVSTPGYGTLPYSTMTRTQRTALDKRRRRSADFDAQPYSDAS
LAPSTIVSKEIMIIYLLSARENDQTRSAELSAXXXXXXXXXXXXXXXXXXXXXXXXXXXXXXXXXXXX
XXXXXXXXXXXXXXXXXXXXXXXXXXXXXXXXXXXXXNSEDPYDDSAHRTGDTMYEPLATRDMNQ
IYEDDRRQKRKPKKERFRRPYVPSTISSATESSVGSGLPRILEIYLPMKNVPYLGLSVCTIDGHIFV
SEIAAEGAVEKDGRMNVGDQILQVNRISFEELGGPQAVRALRDAAASGRPITLYISKYTKGAASEY
DDPLASMASETMPLDVGVWVETAVQNTEKMKALGLDPQEQTITTIDDGTLPFTSTASDDEEKLLY
DQRRNGIPRVVHEDVERKKKESENEHLTELIDPMIVVRNMARPDSGLVVKNRKWLKILVPMSFIG
RDLICWLMEHMTDIHSRKQARVYAARLLAAGLIRHVVSKLTFTEKCYYVFADGILPEGTVDRNSTG
TTSTRADATTEVTYVGSPAPRGLALGARRGIPPHRLETTTLSPVAHDQTWLRRRRDCESPMTND
YASMVGESQLGLNPTHIFGDQKKNHQRPTTTTMTAASQVTSSSLTNGSGGIGGPPPTPLSSTMV
LASPTLSHTCIADSEAGDGAKSRSKIIRS

>Crem-DSH-1
MSIKLEARERAIPTGSNTPSTSTSSDVLRSLLFTSKERKVLSTYTAPPPNRHHSPGVVSQLINKIEQ
KTSPPTTSSRPKKKKKAPPLAQIDDLDLIEDDKTAVEVVKSPRRKKYTGNPLGNLKILNNILPLSKT
SEDKENSNQPTVGKKLKKSKSKSRVEPESEPPFRLKRTLTDLTNNHSPAGSTWRQMVSNALGG
PLKKRLSEGALFFPGTSKTADDCDDEEGDNTKTTTKRRFLQRRKQSEKSAGGANGKESGGSAS
SSFFGALIRLSHSAASLTSLTSLGGSRSNSASPSSSRSNTKEFKEELKPPQPPPPDLISPVAALSKP
IFTLDSSHPVSLTPPSRELRKSKTCQITTDRPPIIPSITISESRSLNRIDRCRPVTVDGSGLTPDRRPL
VSRRSTMSRTMSLIPTSPSLPPLYEEETASTAAMVEEEREDKAQKRRMRRYGGSNTTSTYQGRK
DVAPDASPRRHLIGFLHRTSHLSLTSELSADASFYLIGHRRHLEESTIGSESDARVFSDDDDRGST
TTDFTSVSRQHEKMAKKKKNKRNFRKPSRASSFSSITESSMSLDVITVNLNMDTVNFLGISIVGQT
SNCGDNGIYVANIMKGGAVALDGRIEAGDMILQVNETSFENFTNDQAVDVLREAVSRRGPIKLTV
AKSFENGQSCFTIPRNSREEPVRPIDTQAWIQHTNAMRGMPSIVEESAPTPIPGEWPHGRPPSSS
TVTSNGSNGQNTVVGNGTHIHLDIHTDKKKVVEIMAMPGSGLDIKNRTWLKIPIPMSFLGKYLFPV
HIIIFFYSGSDLVEWLLDRIEGLRERKSARNYAADLLKLKYIAHVVNKVTFTEQCYYVLGDECSGKR
NEELTFIQSKYSDYARFRNEDGGPKYQWTMGMNGMSAGNGSSVMLPPPHLPGGVPPGAFKGM
APSMVSDGESRMYVMHI

>Crem-DSH-2
MTDSPSPIDSSLDYSDVATPCTVIAAKCSIRNQKDLENEEDDLENQEDYTESFQQQEGDSVEQED
LSHIYVDDLSKDFSDAASSVMEPLPKPHTFARTITKVYYHLDDETVPYMVDVHVPPDCITLRDVKR
KLPRTNFKYYCIALDPESGREVKAEIRDDSQRLYPLRCGKFELYLLTVEGSVHSDTSSGRHRKKH
QTSSKGSSSSREYQRAAHHYDNPTPYSDNESQASSIPTYVKKAHAFNRRQASQAYDRHQPRHR
LHERHHQNHYDDSTFDVTTESDDHYRDGVTYYDEDEDDSRSINTDLTSVSQIHLKQRWKQQQQ
REARNKWKRMPSMSTASSSLSSITESSMGLELLTVRLNLQTMPLGMVPYGLKTARGGDAGLYVG
DILDRGAVALDGRIDVGDMISEINNIDLSNYSNEAAAQLLRDAVAPRQFVTLTIAKSIDSRKAVAAAF
TKNTRAEPTRPIDTNEWLKHATNAMKAMPSISEESCSTPIPDEWPTNSSASGTPFGGPPPPSIAC
MNTSTNKKFVVEVMAAPGSGLEIKDREWLKIPIPMSFLGKDLVDWLLDHIQGLRKRGEAGKFAGE
MLKLGYIQHVLNKNKFSENCYYIMGEECADYTQLRAPDGGFKYAQSQTSSASAHSSNNNIFPPS

MYPSQTQPSSAAGVNAHHRNSAILNSMVSGYASMPSSPFPNSKPAVGDCGRTRDDQRSQTSG
SSQGSSRR
>Crem-MIG-5
MEPPCTSDSNQIKVFYYLDDETTPYVSVIDTREGVATLGNFKNSFTKRGYKYYGKELDPDIQREV
KVELTSDSDRLRKSQNGFFEVFLVSTPGYGTLPRNTGTMTRTQRTALDKRRRRSADFDATPYSD
ASLAPSTIVSRRAGEHLAELYTSNSEDPYQYDEHTRRTIDDSSIYEPLGTRDMNKFHDDDRRKRK
QKKERFRRPYVPSTISSATESSVNSGLPRILEIYLPMKNVPYLGLSVCTMDGHIFVSEIAPEGAVEK
DGRVNVGDQILQVNRVSFEDLSGPQAVRALRDAAASKRPITLYISKFARGAPSEYDDPLASMASE
TMPLDVGVWVETAVQNTEKMKALGLDPQEQTMTSVDDGTLPFTSTASDDEERILYDQRRNGIPR
ALLEEAERKKENERNEKAEQLTELIDPIIVVRAMARPDSGLVVKNRKWLKILVPMSFIGCDLIDWLV
EHMTDIHSRKHARLYAARLLAAGLIRHVVSKLTFTEKCYYVFGDGILSTDRNSTDTSGTSGTTMRV
EATTEVTYVGSPAPHAVATRIGRNIPPHRLETTTLSPVAHDQTWLRRRRDCESPMTNDYASMVG
ESQIGMNPAGHYNPYATKNNRQVPAPSQVTTSSLTNGEKLNPLAVLFSTNSVTNSRKRWYWRA
PTDTSVQYYGSSSVSDPITEHHQPRFRRGE
